# BMP Signaling Pathway in Dentin Development and Diseases

**DOI:** 10.3390/cells11142216

**Published:** 2022-07-16

**Authors:** Mengmeng Liu, Graham Goldman, Mary MacDougall, Shuo Chen

**Affiliations:** 1 Department of Developmental Dentistry, School of Dentistry, University of Texas Health Science Center at San Antonio, San Antonio, TX 78229, USA; lium1@uthscsa.edu (M.L.); goldmang@uthscsa.edu (G.G.); 2 UBC Faculty of Dentistry, University of British Columbia, Vancouver, BC V6T 1Z3, Canada; macdougall@dentistry.ubc.ca

**Keywords:** bone morphogenetic proteins (BMPs), BMP receptors, dentin, odontoblasts, Smads, canonical Smad signaling, non-canonical Smad signaling, downstream genes, dentin defects

## Abstract

BMP signaling plays an important role in dentin development. BMPs and antagonists regulate odontoblast differentiation and downstream gene expression via canonical Smad and non-canonical Smad signaling pathways. The interaction of BMPs with their receptors leads to the formation of complexes and the transduction of signals to the canonical Smad signaling pathway (for example, BMP ligands, receptors, and Smads) and the non-canonical Smad signaling pathway (for example, MAPKs, p38, Erk, JNK, and PI3K/Akt) to regulate dental mesenchymal stem cell/progenitor proliferation and differentiation during dentin development and homeostasis. Both the canonical Smad and non-canonical Smad signaling pathways converge at transcription factors, such as Dlx3, Osx, Runx2, and others, to promote the differentiation of dental pulp mesenchymal cells into odontoblasts and downregulated gene expressions, such as those of DSPP and DMP1. Dysregulated BMP signaling causes a number of tooth disorders in humans. Mutation or knockout of BMP signaling-associated genes in mice results in dentin defects which enable a better understanding of the BMP signaling networks underlying odontoblast differentiation and dentin formation. This review summarizes the recent advances in our understanding of BMP signaling in odontoblast differentiation and dentin formation. It includes discussion of the expression of BMPs, their receptors, and the implicated downstream genes during dentinogenesis. In addition, the structures of BMPs, BMP receptors, antagonists, and dysregulation of BMP signaling pathways associated with dentin defects are described.

## 1. Introduction

**Figure 1:**
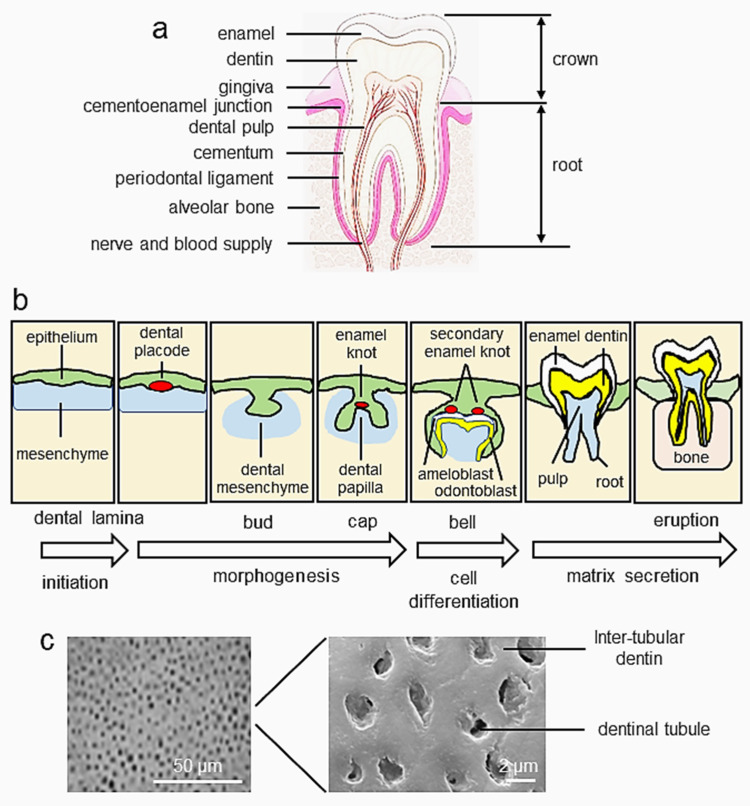
Tooth development. (**a**) The crown of the tooth is covered with enamel, while the root is covered with cementum and periodontal ligament. The cementoenamel junction is located between the enamel and root. The root is surrounded by the alveolar bone through periodontal ligaments. The dentin surrounds the dental pulp. Nerves, lymph, and blood vessels enter the dental pulp from the apical foramen of the tooth and provide nutrition and innervation to odontoblasts and dental pulp. (**b**) Teeth form from the surface epithelium and underlying mesenchyme, and their development is regulated by interactions between the dental epithelium and mesenchyme. The early stages are morphologically similar as the ectodermal placodes develop, form buds, and induce the formation of the dental papilla. In the tooth germ, epithelial folding is regulated by the enamel knot signaling center and it determines the shape of the tooth. During tooth development, it goes through various stages of tooth initiation: bud, cap, bell, and eruption. (**c**) Dentin consists of inter-tubular dentin and dentinal tubules. The figure was partially adapted with permission from Thesleff, 2003, ref. [4].

The tooth is an amazing sensory organ. Teeth perform basic physiological functions in our daily life, playing essential roles in mastication and speech. The tooth is composed of enamel, dental pulp, dentin, and periodontium, consisting of cementum, the periodontal ligament (PDL), alveolar bone, and gingival tissue (Figure 1a) [1,2]. Tooth development and formation are characterized as resulting from sequential and reciprocal interactions between dental oral epithelial and ectomesenchymal cells. The mouse is widely used as an animal model for studying tooth development and signaling pathways. At embryonic day 10 (E10) of mouse tooth development, oral epithelial cells invade, interact with mesenchymal cells, and initiate the mesenchyme directing the process. At E11.5, a local thickening of the dental epithelium becomes evident as the presumptive dental epithelial cells elongate along the apical–basal axis and cell shape changes from cuboidal to columnar, forming the dental placode (the lamina stage) (Figure 1b). At E12.5, the thickened dental epithelial cells derived from the ectoderm of the first branchial arch proliferate and invaginate into the subjacent ectomesenchyme of the neural crest and continue until E13.5, forming a definite tooth bud (the bud stage). The tooth bud is organized into three major parts, including the enamel (dental) organ, the dental papilla, and the dental follicle. Subsequently, the epithelial bud progresses to the cap stage at E14.5 (the cap stage) and goes through specific folding, with enamel knot formation in the dental epithelium to control the patterning of tooth cusps. A small group of ectomesenchymal cells below the dental epithelium proliferate and produce extracellular substances, resulting in an aggregation of these cells termed the dental papilla. On the other hand, ectomesenchymal cells surrounding the enamel organ become dense and form what is known as the dental follicle. Eventually, the epithelial cells from the enamel organ differentiate into ameloblasts, which produce enamel, while mesenchymal cells from the dental papilla differentiate into odontoblasts and produce dentin and dental pulp. The dental follicle produces all the supporting structures of the tooth, such as cementum, alveolar bone, and PDL. When the dental epithelial and mesenchymal cells proceed to histodifferentiation and morpho-differentiation and the enamel organ forms a bell-shaped morphology at E16.5 (the bell stage), these cells begin to differentiate into ameloblasts and odontoblasts, respectively [3]. During the late bell stage of tooth development, odontoblasts synthesize and produce dentin extracellular matrix (ECM), including collagen and non-collagenous proteins (NCPs) required for dentin formation.

During human tooth development, the initiation stage begins in the fetus at about the sixth and seventh weeks of age [2]. The bud stage occurs at about the eighth week, and the cap stage occurs around the twelfth week as a small group of ectomesenchymal cells proliferate, resulting in an aggregation termed the dental papilla. At this point, the dental epithelial cells at the tooth bud grow around the group of ectomesenchymal cells, taking on the appearance of a cap, and become the enamel organ covering the dental papilla. A condensation of ectomesenchymal cells is called the dental follicle surrounding the enamel organ. Finally, the enamel organ will produce enamel, the dental papilla will produce dentin and pulp, while the dental follicle will produce all the supporting structures of the tooth, the periodontium consisting of the cementum, PDL, gingiva, and alveolar bone [2]. At the bell stage, which occurs around 15th week of fetal development, histological and morphological differentiation take place. The bell stage is divided into the early bell stage and the late bell stage [2]. The cells in the enamel organ differentiate into the stellate reticulum because of their star-shaped appearance. Cells on the periphery of the enamel organ separate into four principal layers. Cuboidal cells on the periphery of the enamel organ are called the outer enamel epithelium (OEE). The columnar cells of the enamel organ adjacent to the enamel papilla are known as the inner enamel epithelium (IEE). The cells between the IEE and the stellate reticulum form a layer known as the stratum intermedium. The rim of the enamel organ where the OEE and IEE join is called the cervical loop [2]. At the maturation stage, individual tooth development varies. 

Dentin formation, termed dentinogenesis, is the first identifiable feature in the crown stage of tooth development. Dentin formation starts at the onset of odontoblast differentiation. Odontoblasts, the dentin-forming cells, deviate from neural crest-derived mesenchymal cells, which differentiate to form odontoblasts in specific spatial–temporal patterns, originating at the principal cusp tip and advancing toward the base of the teeth [4,5,6]. Odontoblasts are mitotic cells organized as a single layer of barrier cells along the peripheral part of the dental pulp from the predentin and attach to each other by junctional complexes. Additionally, odontoblasts retain dentin metabolism in vital teeth throughout their life, as they synthesize and secrete the organic ECM proteins. Dentin is composed of hydroxyapatite crystals (HA, 70% by weight), water (approximately 12%), and organic matter, including collagens and NCPs (about 20%) [7,8].

Dentin is a thick, highly mineralized tissue layer subjacent to the enamel and consists of inter-tubular dentin and dentinal tubules (Figure 1c), which act as a secondary barrier against infections of the dental pulp cavity [9]. Dentin is divided into two main anatomical parts: the crown and root of dentin (Figure 1a). The crown of the dentin is covered by enamel, while the root of the dentin is surrounded by cementum and PDL, a fibrous connective tissue structure that connects the root cementum to the alveolar bone. Importantly, dentin is a critical element in the function of the tooth because it anchors the tooth to the maxilla or mandible. Consequently, the loss of dentin leads to lessening bone support and thus disturbs tooth function. Additionally, during resting and mastication states, the root dentin assists balance and transmits occlusal forces through PDL to the alveolar bones and serves as an ingress for blood and lymph vessels with abundant capillaries, as well as nervous bundles to supply nutrition and sensation to the tooth [10,11]. Furthermore, dentin is a stronger substance with respect to tension than enamel. When the tensile stress of enamel is exceeded, the fracturing of enamel rods can occur and create an environment for the potential formation of wedge-shaped lesions. However, dentin not only supplies nutrition to enamel but also supports enamel and is responsible for the alleviation of enamel pressure.

Different types of dentin are classified as either primary dentin, secondary dentin, or tertiary dentin. Primary dentin forms through a unique process. Odontoblasts increase in size, eliminating the availability of any extracellular resources to contribute to an organic matrix for mineralization. In addition, the larger odontoblasts cause collagens and NCPs to be secreted in smaller amounts, which results in more tightly arranged, heterogeneous nucleation that plays a role in mineralization. Other materials, such as lipids, phosphoproteins, and phospholipids, are also secreted. Secondary dentin is formed after the root formation is complete and occurs at a much slower rate. Secondary dentin is not formed at a uniform rate along with the tooth but instead forms faster along sections closer to the crown of a tooth. This development continues throughout life and accounts for the smaller areas of dental pulp found in older individuals. Tertiary dentin, also known as reparative dentin, forms in reaction to stimuli, such as attrition or dental caries. Tertiary dentin formation represents an important defense mechanism and a regeneration property of the dentin–pulp complex. When dentin is destroyed, as in dental caries and attrition, dental mesenchymal progenitors/stem cells from the dental pulp can differentiate into odontoblast-like cells, forming tertiary dentin.

The molecular regulation of tooth development and morphogenesis leading to dentin formation has been studied. Indeed, a lot of work has shown that the network controlling dentin development encompasses the action of numerous signaling pathways, including growth factors, transcriptional factors, and other components, which execute various stages of tooth development and formation [4,12,13]. However, the systematic molecular network of bone morphogenetic proteins (BMPs) and their antagonists controlling the early and late stages of dentin development and formation has not been described in detail. In this review, we explore some of the unique transcriptional regulatory and signaling networks involving BMPs and their antagonists, as well as accurate balances of BMPs and their antagonists during dentinogenesis that may play critical roles in regulating dentin formation and regeneration. Based on recent advances in our understanding of how these networks function during tooth morphogenesis, we review what is currently known about the cellular and molecular mechanisms involved in the formation of dentin and the potential implication of progenitor-/stem cell-mediated dentin development and regeneration, as well as their relevance in human diseases. Finally, we suggest future directions for investigating the molecular and cellular regulatory mechanism of dentin development and regeneration.

## 2. BMP Family

**Figure 2:**
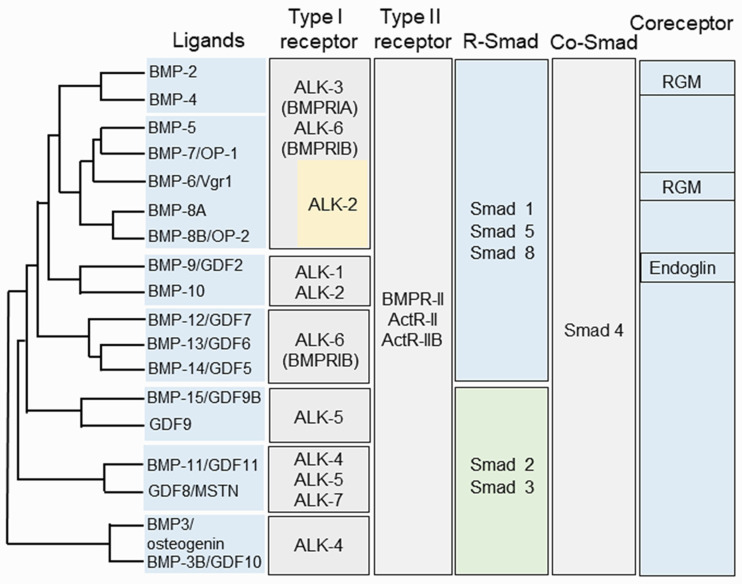
Phylogenetic analysis of the BMP family. Relationship between BMP/GDF ligands, type I receptors, type II receptors, Smad proteins, and RGM in signal transduction. ActRIIA/B, activin type II receptor A or B; ALK, activin receptor-like kinase; BMP, bone morphogenic protein; BMPRI, BMP type receptor I; BMPRII, BMP type receptor II; GDF, growth differentiation factor; MSTN, myostatin; OP, osteogenic protein; RGM, repulsive guidance molecules; Vgr, Vg-related protein. The figure was partially adapted with permission from Katagiri and Watabe, 2016, ref. [22].

In 1965, Urist first reported that demineralized bone matrices from rabbits were able to induce the ectopic formation of cartilage and bone when these matrices were implanted into muscular tissues and thus named them “bone morphogenetic proteins (BMPs)” [14]. However, the proteins responsible for cartilage and bone formation remained elusive until the purification and identification of bovine BMP-3 protein sequences by Wang et al. [15]. Subsequently, Wozney and colleagues cloned and characterized cDNAs for human BMP-1 through BMP-4 [16]. They concluded that BMP-2, -3, and -4 belong to novel members of the transforming growth factor beta (TGF-β) superfamily [16]. Later, the additional BMP genes were cloned and identified based on amino acid sequence homology [17,18,19]. It has been shown that BMPs impact other biological activities, although the bone-inducing bioactivity is unique to BMPs among the TGF-β family members [20]. Currently, more than 20 BMPs have been discovered and the BMP family is categorized into several subgroups based on sequence homology and biological functions, i.e., (1) the BMP-2 and BMP-4; (2) the BMP-3B/GDF10 and BMP-3/ostegenin; (3) the BMP-5, BMP-6/Vgr1, BMP-7/OP1 and BMP-8; (4) the BMP-9/GDF2 and BMP-10; (5) the BMP-11/GDF11 and GDF8/MSTN; (6) the BMP-12/GDF7, BMP-13/GDF6, and BMP-14/GDF5; and (7) the BMP-15/GCF9B and GDF9 subgroups [21,22,23,24]. The BMPs interact with their receptors and form a complex which regulates downstream gene expression via canonical Smad and/or non-canonical Smad signaling pathways (Figure 2).

### 2.1. BMP Ligand Expression during Tooth Development and Mutations of BMPs Related to Dentin Defects

#### 2.1.1. BMP-2

**Figure 3:**
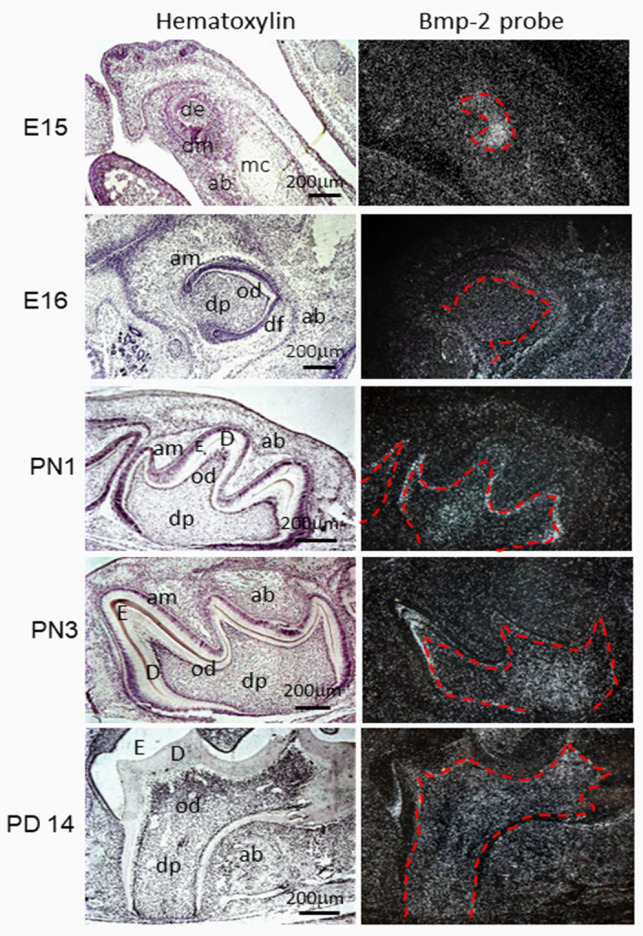
Bmp-2 expression during mouse tooth development. At the cap stage (E15) of mouse tooth development, Bmp-2 mRNA was highly expressed in the dental epithelium and moderately detectable in dental mesenchymal cells, osteogenetic mesenchyme, and Meckel’s cartilage. At the early bell stage (E16), the Bmp-2 transcript was present in ameloblasts, odontoblasts, dental pulp cells, dental follicles, and mesenchyme in the alveolar bone. At postnatal days ranging from 1 to 14, an in situ hybridization signal was apparent in odontoblasts, dental pulp cells, and osteoblasts in the alveolar bone and was weakly detected in ameloblasts. The figure is adapted with permission from Chen et al., 2008, ref. [5]. ab, alveolar bone; am, ameloblasts; mc, Meckel’s cartilage; D, dentin; de, dental epithelium; df, dental follicle; dm, dental mesenchyme; dp, dental papilla; E, enamel; E, embryonic day; od, odontoblasts.

At the initiation stage of mouse tooth development (E10–12), the dental lamina is formed as an epithelial clustering, distinguishing the dental epithelium and mesenchyme. Bmp-2 gene transcription is seen in those areas of dental lamina where buds have started to form [5,25,26,27]. At the bud stage (E12–13), Bmp-2 expression is detectable in the dental epithelium and mesenchyme throughout the budding period. At the cap stage, Bmp-2 expression is prominent at E14 and mainly localized at the epithelial enamel knot during the late cap stage. Bmp-2 expression then expands to the neighboring inner dental epithelium, where the secondary enamel knots will be formed, and its signal can be seen in dental mesenchyme using in situ hybridization and immunohistochemistry analyses. At the bell stage, in situ hybridization and immunostaining showed that Bmp-2 expression is detected in the dental mesenchymal cells [28,29]. Later, Bmp-2 expression spreads to the dental papilla and is intense in the pre-odontoblasts. In postnatal days (PNs), Bmp-2 is continually expressed in odontoblasts and ameloblasts and is detected in the dental papilla as well as adjacent tissues, including the dental follicle, PDL cells, cementoenamel junction, Hertwig’s epithelial root sheath (HERS), and osteoblasts in alveolar bones analyzed by in situ hybridization and immunohistochemistry assays (Figure 3 and Table 1) [30,31]. Gao et al. reported that in miniature pigs Bmp-2 was expressed in both the enamel organ and in the dental mesenchyme at E40 and E50 and was expressed mainly in pre-odontoblasts at E60 by in situ hybridization and immunohistochemistry assays [29]. In the human fetus, BMP-2 expression was detected in the enamel organ and dental papilla, with a slightly stronger intensive signal in the inner epithelium of the incisor and molar in the developing human primary tooth germ at the cap stage (12th embryo week). At the bell stage (14th week of fetal development), BMP-2 expression pattern was similar to that at the cap stage in the incisor but was mainly restricted to the inner enamel epithelium in the molars, as determined by in situ hybridization assay [28]. 

BMP-2 is involved in specifying the fate of dental pulp and dental pulp stem cell (DSPC) differentiation into odontoblast-like cells and stimulates tooth-related gene expression [5,32]. Genetic variants of the BMP-2 gene in humans are associated with susceptibility to non-syndromic tooth agenesis, and the most commonly missing teeth were found to be the mandibular incisors, followed by the mandibular premolars and maxillary incisors [33,34]. Homozygous mutant embryos for Bmp-2 showed developmental abnormalities and died at embryo day 9.5 due to defects in amnion/chorion and cardiac development [35]. Recently, Bmp-2 conditional knockout (cKO) mice have been generated and revealed important roles of Bmp-2 in dentinogenesis. Mice lacking Bmp-2 (Osx-Cre;Bmp-2fx/fx, Collα1-Cre;Bmp-2fx/fx, Wnt1-Cre-;Bmp-2) exhibit dentinogenesis imperfecta type II and type III (DGI-II, DGI-III), and dentin dysplasia type II (DD-II) [32,36,37,38,39]. Dentin defects in Bmp-2 KO mice occurred in the late stages of tooth development, including a wide dental pulp cavity, wide pre-dentin, thin dentin, tooth attrition, delayed root eruption, and impairment of odontoblast differentiation and biomineralization. Additionally, the downstream gene expression of Bmp2 was reduced, including transcriptional factors, Dlx3, Osx, phosphorylated Smad1/5/8, Runx2, and tooth-related genes, Alp, Collα1, dentin matrix protein 1 (Dmp1), and dentin sialophosphoprotein (Dspp) [32,36,37,38,39].

#### 2.1.2. BMP-3

At the initiation stage of tooth development, there is no Bmp-3 expression in tooth tissue, but Bmp-3 transcripts are visible in the lingual aspect of the dental lamina at the bud stage. At the cap stage, expression of Bmp-3 is detected in the dental papilla mesenchyme underlying the enamel knot and mesial part of the dental mesenchyme. Bmp-3 expression is also seen in the dental follicle mesenchyme and its intense expression is observed in the lingual side of the outer dental epithelium. Bmp-3 expression is strongly detected in osteoblasts of alveolar bones as well as in peripheral chondrocytes of Meckel’s cartilage. At the bell stage and PNs, expression of Bmp-3 is apparent in odontoblasts, but its expression gradually decreases with the advancement of odontoblast differentiation. Bmp-3 expression is observed in odontoblasts, dental follicles, PDL cells, cementoblasts, tooth root-lining cells, and osteoblasts on the active bone-forming surface [25,30,31,40,41,42]. BMP-3 expression was found to be evenly distributed in human incisors and molars at the cap and bell stages (12th and 14th weeks of fetal development, respectively) by in situ hybridization assay [28]. 

#### 2.1.3. BMP-4

At the tooth initiation stage, the Bmp-4 transcript is detected in the oral epithelium and dental mesenchyme and continues to the dental epithelium and the condensed dental mesenchyme around the epithelial bud [25]. At the bud stage, Bmp-4 is detected in both the dental epithelium and mesenchyme with similar intensities [25,26]. At the cap stage, Bmp-4 expression is present in the epithelium and primary enamel knot cells distant from the basement membrane in the dental papilla mesenchyme and dental sac. At the bell stage, expression of Bmp-4 becomes prominent in the dental papilla, pre-odontoblasts, inner enamel epithelium, outer enamel epithelium, and stellate reticulum, but is absent in the dental epithelium with the removal of the enamel knot. Bmp-4 expression is weak in the central cells of the dental papilla [26,28,29]. During PNs, Bmp-4 expression is observed in the odontoblasts, ameloblasts, and osteoblasts in alveolar bone [30]. In miniature pigs, in situ hybridization and immunostaining analyses showed that Bmp-4 was expressed mainly in the epithelium, with some weak expression in the mesenchyme at E40. At E50, Bmp-4 expression was stronger in the mesenchyme and weaker in the epithelium. Bmp-4 expression was mainly detected in the mesenchyme at E60 [29]. In human tooth germs, BMP-4 expression was visible in the inner enamel epithelium and dental papilla, with slightly weaker expression in outer enamel epithelium in the human primary incisor at the cap stage (12th embryo week), whereas BMP-4 expression was intense in the molar, with a higher level in the inner enamel epithelium and dental mesenchyme underneath it and a lower level in the remaining dental tissues as determined by anti-BMP-4 mRNA probe. By the bell stage (14th week of the fetus), BMP-4 transcription was more prominent located in the inner enamel epithelium and dental papilla than in the outer enamel epithelium and the stellate reticulum of the incisor and the molar. Immunohistochemistry further confirmed the BMP-4 expression patterns in the developing human tooth germs [28]. 

BMP-4 plays an important role in the signaling interactions between tooth bud epithelium and mesenchyme during tooth morphogenesis [43]. BMP-4 mutation in humans was associated with different types of dental anomalies, including tooth agenesis [43,44]. Bmp-4 mutation influences storage or transport of the mature Bmp-4 protein in the ECM of the developing teeth and significantly decreased levels of phosphorylated Smad-1/5/8 in the cells. Homozygous Bmp-4 null mice die between E6.5 and E9.5 and show little or no mesodermal differentiation [45]. Thus, Bmp-4 cKO mice (Collα1-Cre;Bmp-4fx/fx) were generated and exhibited dentin deficiency at the late stages of mouse tooth development [46]. In Bmp-4 mutant mice, dentin thickness was reduced and root eruption was delayed, while dental pulp chamber volume was increased. Odontoblastic cell differentiation was impaired in Bmp-4 cKO mice, and Bmp-4 deletion led to decreased expression of osteocalcin, Dmp1, Col1α1, Dlx5, Osx, and phosphorylated Smad1/5/8 in the mutant odontoblasts [43]. 

Bmp-2 and Bmp-4 double cKO mice (DcKO, Dmp1-Cre; Bmp-2 fx/fx/Bmp-4 fx/fx) were generated [47]. The Bmp-2/Bmp-4 DcKO mice showed reduced dentin thickness and enlarged dental pulp cavities compared to the wild-type mice. Dentin in Bmp-2/Bmp-4 DcKO mice was characterized by small, disorganized dentin fibers and a wide predentin layer; decreased expression of bone sialoprotein (Bsp), Dmp1, and Dspp genes was also observed. This indicates that the loss of Bmp signaling alters gene expression in odontoblasts and dentin and leads to improper odontoblast differentiation and odontogenesis. 

#### 2.1.4. BMP-5

Expression of Bmp-5 is not detected at the initiation, bud, and cap stages of mouse tooth development [25]. However, Bmp-5 transcripts are present in the differentiating pre-ameloblasts during the late bell stage [25]. On PNs, Bmp-5 expression is evident in ameloblasts during amelogenesis and is seen in chondrocytes and osteoblasts [48,49,50]. Mutations of BMP-5 gene are associated with short ears in humans and mice [51,52]. Additionally, genetic variants of BMP-5 are associated with susceptibility to osteoarthritis (OA) [53,54]. Bmp-5 is required for neural crest progenitor cell survival and proliferation through the Smad–Msxb and Mek–Erk–Id3 signaling pathways, respectively [55]. Although BMP-5 is expressed in dental cells during tooth development, the roles of BMP-5 in tooth formation have not been elucidated in detail.

#### 2.1.5. BMP-6

During the initiation stage of tooth development (E10–E12), the Bmp-6 transcript is not detectable, but at the bud and cap stages Bmp-6 expression is weakly observed in dental mesenchyme, dental lamina, and enamel-organ epithelial cells [25,56]. During the bell stage, Bmp-6 protein reaction in developing odontoblasts and dental papilla can be found to be positive using anti-Bmp-6 immunostaining. Bmp-6 immunoreactivity is weakly present in the outer enamel epithelium and stratum intermedium but is not visible by developing ameloblasts. The dental lamina continues to react positively and the dental follicle negatively. When dentin matrix production begins, polarized odontoblasts appear strongly Bmp-6-immunoreactive. Positive staining with Bmp-6 antibody is also observed in the dental pulp. Expression of Bmp-6 persists in secretory odontoblasts during the period of dentin formation, whereas enamel organ epithelial, secretory ameloblasts, and dental follicles react negatively [56]. On PNs, Bmp-6 protein is present in mesenchymal cells in the freshly formed periodontium and in the layer of cementoblasts along with the root, the HERS, as well as in the osteoblasts lining the alveolar bones [57]. 

Mutations of BMP-6 in humans and mice induce massive iron overload [58,59,60]. Despite their severe iron overload, Bmp-6 null mice are viable and fertile. Hepatocytes of Bmp-6 mice express low levels of phosphorylated Smad1/5/8/ proteins and hepcidin synthesis is significantly reduced. This indicates that Bmp-6 is critical for iron homeostasis. Additionally, histomorphometry of the proximal tibial metaphysis exhibited a defect in bone formation immediately adjacent to the growth plate in Bmp-6 KO mice compared to wild-type mice following estrogen treatment, and growth plate functions were decreased in Bmp-6 mutant mice after estrogen treatment, causing an impaired cancellous bone. This suggests that Bmp-6 also plays a physiological function in maintaining growth plate function [61,62]. Cleves et al. found that Bmp-6 is expressed in epithelial and mesenchymal cells during tooth development in sticklebacks and is required for viability, growth, and tooth patterning [63]. Homozygous deletion of the Bmp-6 gene in the fish resulted in reductions in both tooth number and tooth plate area at the early juvenile stage compared to wild-type or heterozygous fish. In addition, heterozygous mutations of Bmp-6 revealed a reduction in tooth number and tooth plate area in the late juvenile and adult stages compared to wild-type fish at the same ages. Gene expression of TGF-β and Smad signaling in Bmp-6 null fish was found to be decreased compared to wild-type fish [63]. 

#### 2.1.6. BMP-7

At the initiation stage of mouse tooth development (E10–E12), Bmp-7 transcription is visible throughout the oral epithelium at the time of the formation of the dental lamina; however, in other parts of the epithelium covering the facial processes, Bmp-7 mRNA is weakly expressed [25,64]. At the bud stage, Bmp-7 expression is localized at the early bud stage in the tip of the bud, and its expression patterns are seen in these areas of the dental lamina where it starts to form a bud. At the cap stage, the anti-Bmp-7 probe is apparent and restricted to the epithelial enamel knot. As the enamel knot begins to disappear during the late cap stage (E15), Bmp-7 expression spreads to the neighboring inner dental epithelium and its expression appears to localize to sites where the secondary enamel knots will be formed [25,30,65]. At the bell stage, the Bmp-7 transcript continues throughout the inner enamel epithelium to the preameloblasts. Its expression is detectable in the dental papilla and the preodontoblasts when they start to secrete predentin matrix (E-19). On PNs, during deposition of dentin and enamel, the expression patterns of Bmp-7 appear in the dental papilla, odontoblasts, and in functional ameloblasts [25]. During root development, Bmp-7 staining is positive in PDL cells, dental follicles, tooth root-lining cells, chondrocytes, osteoblasts, osteocytes, and osteoclasts in alveolar bones [31,40,41,65]. In miniature pigs, Bmp-7 gene expression patterns in developmental teeth were similar to Bmp-2 as determined by anti-Bmp-7 probe and immunohistochemistry assays. However, Bmp-7 expression was also present in the inner enamel epithelium at E60 [29]. 

In human fetuses of 12th (equal to the cap stage of mouse tissues) and 14th (equal to the bell stage of mouse tissue) week gestations, BMP-7 expression was weakly detected in both the incisors and the molars at the cap and bell stages of the human tooth germs. BMP-7 expression patterns were rather consistent in the dental epithelium and mesenchyme at the cap and bell stages and were relatively higher in the inner enamel epithelium than in the dental mesenchyme and inner enamel epithelium [28]. However, BMP-7 expression was detected in both the dental epithelium and mesenchyme of human fetuses in the 12^th^ week of gestation, in contrast to mouse tooth germs at the cap stage [25,28,29,64,65]. 

Mutations of BMP-7 in humans and mice are associated with anomalies in several organs, including the kidneys, eyes, skeleton, and genitourinary ducts [66,67,68]. Genetic variants of BMP-7 are suspected to be implicated in molar–incisor hypomineralization (MIH) [69]. Global homozygous Bmp-7-deficient mice die shortly after birth because of poor kidney development. Histochemistry analysis demonstrated that mutant embryos during organ development reveal failure of metanephric mesenchymal cell differentiation, resulting in a virtual absence of glomeruli in newborn kidneys. Additionally, Bmp-7 null mice display eye deficiencies that appear to originate during lens induction. Bmp-7 mutant mice exhibit minor defects in the skeleton [70,71]. Heterozygous Bmp-7 mice exhibited significantly increased tooth sizes in adult incisors compared to wild-type adult mice. Cell proliferation in the cervical loop at the bell stage (E15) of heterozygous Bmp-7 mice was significantly higher than in wild-type mice of the same age [72]. Bmp-7 cKO mice (Wnt1-Cre:Bmp-7 fx/fx) were generated and mutant molars were short and broad both mesiodistally and buccolingually and displayed extra cusps on the first upper and lower molars. Multiple morphotypes of the ‘extra cusp’ morphology were present in both the upper and lower first molars in the null mice [73]. In addition, Bmp-7 cKO mice showed interruption in the initial onset of mineralization and a lack of dentin/enamel formation at birth and delayed tooth eruption. Expression of Dspp and amelogenin genes in odontoblasts and ameloblasts were decreased in Bmp-7 cKO mice. Bmp-7 contributes to the regulation of initiation of tooth mineralization via a complex epithelial–mesenchymal Bmp/Wnt signaling pathway [74]. 

#### 2.1.7. Activin-like Kinase-1 (ALK1, ACVRL1, or TSR-1) and ALK2 (ACVR1, ActR-IA)

At the initiation stage of mouse tooth development (E-12), Alk-2 mRNA transcripts are not present in the tooth primordium of mouse embryos but can be seen in other tissues, such as the brain [75]. At the bud stage (E13–13.5), Alk-1 and Alk-2 proteins are detected in the dental epithelium. The expression of Alk-1 and Alk-2 proteins is apparent in the basal layer of the dental epithelial bud. Both proteins are weakly present in the dental mesenchyme [76,77]. Alk-2 mRNAs are expressed in dental pulp cells in adult rat, bovine, and human teeth, but the Alk-1 gene is only detected in rat dental pulp cells [78,79,80,81]. Additionally, the Alk-2 protein is detected in HERS, the dental follicle, osteoblasts of alveolar bone, PDL cells, and tooth root-lining cells [31,82]. An in vitro study showed that the expression level of Alk-2 is increased with PDL cell differentiation [79,82]. Mutations of ALK1 cause type 2 hereditary hemorrhagic telangiectasia (HHT2), a devastating disorder that leads to arteriovenous malformations [83,84,85]. However, mutations of ALK-1 related to tooth development and formation have not been described so far.

Alk-2 cKO mice (Osx-Cre; Alk-2 fx/fx) exhibited dentin defects in molars and osteodentin formation in incisors. Alk-2 cKO mice displayed striking tooth phenotypes characterized by enlarged dental pulp cavities, wide predentin, and thin dentin, as well as delayed odontoblast differentiation in molars compared to wild-type mice. Expression of Dspp, Dmp1, and Osx in odontoblasts of Alk-2 null mice was reduced, whereas Bsp expression was increased in the mutant odontoblastic cells, leading to an alteration of cell fate from odontoblasts to osteoblasts. In addition, the expressions of the WNT antagonists Dkk1 and Sost were downregulated, but β-catenin was upregulated in Alk-2 cKO incisors. This indicates that Alk-2 plays an important role in dentinogenesis [86]. Furthermore, mutations of the ALK-2 gene in humans and mice cause a rare genetic disorder known as fibrodysplasia ossificans progressiva (FOP) characterized by progressive heterotopic ossification (HO), where especially muscles, tendons, and ligaments are converted into bone. The mutations of ALK-2 enhance the BMP–Smad and BMP–p38 signaling pathways [87,88,89]. 

#### 2.1.8. ALK-3 (Bmpr-1A)

At the bud stage of mouse tooth development, Alk-3 peptides are detected in the dental epithelium but are mainly localized to the tip and stalk and oral epithelium. At the cap stage, immunostaining of Alk-3 is present in the dental epithelium and the stalk as well as the upper part of the enamel organ. Its signal is also seen in dental mesenchyme. At the late cap stage (E15), Alk-3 expression is localized in the cells of the primary enamel knot distant from the basement membrane, in the stellate reticulum, and in the outer dental epithelium. Additionally, at this stage, the staining of this protein appears in the dental mesenchyme. At the bell stage, Alk-3 protein is present in the stratum intermedium, the stalk, the inner dental epithelium, and ameloblasts. The immunohistochemistry of Alk-3 is positive in the dental papilla and odontoblasts [26,81]. On PNs, besides ameloblasts and odontoblasts, Alk-3 protein expression is observed in HERS, the dental follicle, osteoblasts in alveolar bones, PDL cells, and tooth root-lining cells [31,79,82]. Expression of Alk-3 is increased with PDL cell differentiation [31,82]. Alk-3 mRNA transcription is detected in dental pulp cells from adult rat, bovine, and human teeth and is increased with dental pulp cell differentiation [78,79,80]. 

Global null mutation of the Alk-3 gene causes embryonic lethality in mice and animals die at E9.5. Homozygous deleted mice with morphological defects are detected at E7.5. No mesoderm forms in the mutant embryos, suggesting that Alk-3 is required for the inductive events, leading to the formation of mesoderm during gastrulation [89]. Alk-3 cKO mice (K14-Cre;Alk-3 fx/fx; Wnt1-Cre;Alk-3 F/-) showed an arrest of tooth development at the bud/early cap stages; the tooth buds were significantly smaller and less well developed in mutant embryos than in controls. Defective tooth development is accompanied by the downregulation of BMP-responsive genes, including Bmp-4, Msx1, and Pax9, in the mesenchyme at the bud stage and reduced cell proliferation levels in the dental mesenchyme [90,91]. In Osx-Cre;Alk-3 fx/fx mice, loss of Alk-3 in odontoblasts led to impaired dentin formation and short molar roots at PN 21. Alk-3 cKO mutants exhibited thin dentin, reduced dentin matrix production, and decreased expression of Osx, Dspp, phosphorylated Smad1/5/9, and phosphorylated p38 as compared to controls [92]. Transgenic mice expressing a constitutively active form of Alk-3 (caAlk-3; Wnt1-Cre; pMescaAlk-3) were generated, and all binary transgenic mice of caAlk-3 died shortly after birth [93]. The transgenic molars showed less differentiated odontoblasts and ameloblasts and dentin deposition was lacking in molars at PN 0. The number of phosphorylated Smad1/5/8-positive cells was significantly increased in the dental mesenchyme of the molar germ at E13.5 in the transgenic mice. The expression of odontogenesis, amelogenesis differentiation markers, Dspp, and amelogenesis were remarkably reduced within odontoblasts and ameloblasts in PN 0 molars in the transgenic mice. Overall, this indicates that the accurate balance of Alk-3 expression levels is essential for tooth development and formation.

#### 2.1.9. ALK-5 (TGFβR1 or NDSTN)

At the bud stage of mouse tooth development, the dental lamina is invaginated into the condensed mesenchyme, and immunostaining of Alk-5 is detected in the dental epithelium and dental mesenchyme of the tooth bud [94,95]. At the cap stage, Alk-5 mRNA transcription is seen in the dental epithelial and mesenchymal cells and tooth germ mesenchymal cells (TGMCs) from the lower first molar tooth germs at E15.5 [94,96]. Expression of Alk-5 protein is observed in undifferentiated mesenchyme cells around Meckel’s cartilage through E12 to E16 of mouse embryogenesis [94]. At the late bell stage (E18.5), Alk-5 protein is clearly evident in the inner dental epithelium layer and weakly stained in dental mesenchymal cells [97]. During PNs, the expression level of Alk-5 was elevated in secretory ameloblasts and odontoblasts in developing mouse teeth and immortalized mouse ameloblast-lineage cells [97]. Alk-5 mRNA transcription is observed in dental pulp cells and odontoblasts from rat, bovine, and human teeth [79,80,98,99]. The expression level of Alk-5 increased with dental pulp cell differentiation [79,98,99]. 

#### 2.1.10. ALK-6 (BMPR-IB)

At the bud stage of tooth development, Alk-6 is present in the dental epithelial and mesenchymal cells surrounding the epithelial bud [26,81]. In the mesenchyme, the staining is intense on the buccal side. At the cap stage, expression of Alk-6 in the epithelium is more prominent in the inner dental enamel than in the outer dental enamel, and the inner cells of the primary enamel knot are intensely stained. The relative amount of Alk-6 expression is slightly decreased from E14 to E17 but is increased at the bell stage when cell differentiation occurs. At the bell stage, the expression of Alk-6 appears strong in ameloblasts and odontoblasts. ALK-6 expression patterns at the cap and bell stages of human tooth development are similar to those in mice. ALK-6 is present in both the whole-enamel organ and the dental mesenchyme, with the strongest expression levels in the inner dental enamel at the cap and bell stages [28,97]. During PNs, ALK-6 is strongly apparent in secretory ameloblasts and odontoblasts, as well as dental pulp cells from human, rat, and bovine molars [78,80]. With tooth root development, expression of ALK-6 is positive in HERS, tooth root-lining cells, the dental follicle, PDL cells, and osteoblasts in alveolar bones [31,82,100]. 

Homozygous Alk-6 null mice are viable and fertile. However, in Alk-6-deficient mice, proliferation of prechondrogenic cells and chondrocyte differentiation in the phalangeal region were significantly reduced. In adult null mice, the proximal interphalangeal joint was absent, the phalanges were replaced by a single rudimentary element, and the metacarpals and metatarsals were reduced, but the lengths of the radius, ulna, and tibia were not affected [101]. Recently, it has been reported that Alk-6 KO resulted in osteopenia in 8-week-old male mice, and the phenotype was transient and gender-specific. The decreased bone mass was not related to either the changes in osteoblastic bone formation activity or osteoclastic bone resorption activity in vivo. Thus, Alk-6 plays a unique role in maintaining bone mass and transducing BMP signaling [102]. In Alk-3 cKO mice, tooth development was arrested at the bud/early cap stages and the tooth buds were significantly smaller and less well developed in mutant embryos than in controls. Expression of Bmp-4, Msx1, and Pax9 at the bud stage and of Dspp and amelogenin at PN 0 was reduced in the mesenchyme; reduced cell proliferation levels in the dental mesenchyme were also observed [90,91]. However, when Alk-3 cKO mice (Wnt1-Cre;Alk-3F/-) were crossed with a conditional transgenic line containing a constitutively active form (with Gln203 to Asp change) of Alk-6 (named caAlk-6) to generate mice lacking Alk-3 but expressing caAlk-6 in the neural crest cells (Wnt1-Cre;Alk-3F/-; caIb), most Wnt1-Cre;Alk-3F/-;caIb embryos died mid-gestation. caAlk-6 only partially substitutes for the loss of Alk-3 in the cranial neural crest cells to regulate tooth development; it is unable to fully replace Alk-3 function, as evidenced by delayed odontoblast and ameloblast differentiation as well as a reduction in Dspp and amelogenin gene expression within odontoblasts and ameloblasts at PN 0 [91]. In addition, in immortalized ameloblast-lineage cells, Alk-6 and odontogenic ameloblast-associated protein (ODAM) display similar expression patterns, and Alk-6 physiologically interacts with ODAM, resulting in an increase in ODAM protein phosphorylation mediated by BMP-2. The phosphorylated ODAM induces MAPK signaling cascades, inducing ameloblast differentiation and enamel mineralization [103].

#### 2.1.11. Type II Bmp Receptor (Bmpr-II) 

Immunohistochemistry showed that BmpR-II protein is present in the dental epithelium and in the dental mesenchyme at the bud stage (E13) in mouse tooth development. BmpR-II is apparent in the internal part of the bud, in the stalk, and in the oral epithelium. At the cap stage (E14), BmpR-II is mainly localized in the mesenchyme, but weak staining is present in the oral epithelium by RT-PCR and immunoblotting assays. At the late cap stage (E15), BmpR-II is detected in the mesenchyme and in the cells of the primary enamel knot near the stratum intermedium. BmpR-II immunostaining is intense in the stalk and in the oral epithelium. BmpR-II protein is also stained in the outer dental epithelia and in the cells of the stellate reticulum, close to these outer dental epithelia. At the bell stage, expression of BmpR-II is seen in the dental papilla, odontoblasts, and ameloblasts as well as the stratum intermedium [26,81]. BmpR-II is also expressed in the dental pulp and preodontoblasts in rat, bovine, and human teeth [78,79,80]. Furthermore, during mouse root development from PNs 5 to 23, the study demonstrated that expression of BmpR-II was detected in odontoblasts, ameloblasts, HERS, dental follicles, osteoblasts in alveolar bone, PDL cells, and tooth root-lining cells [31,100].

### 2.2. Structure of BMPs and Their Partners

Cells such as odontoblasts and osteoblasts produce BMPs, which are secreted into the ECM. All BMPs, like other TGF-β family members, initially act as a single chain containing a larger amino-terminal prodomain and a smaller carboxy-terminal mature signaling domain [104,105,106]. TGF-β ligands are initially synthesized as large dimers containing a secretion signal peptide in the amino-terminal element termed the prodomain and a cystine-knot mature domain in the carboxy-terminal domain [107]. TGF-β-secreted precursors are attached to a prodomain called a latent associated peptide (LAP), which regulates the folding of the prodomain and prevents interaction with the receptor at the cell membrane [108]. By contrast, BMPs do not display such latency [106] and are generally catalyzed by cellular serine endoproteases to liberate the mature BMP polypeptides before secretion [109]. Recently, studies have shown that although BMP processing is predominantly localized intracellularly, it might also occur at the plasma membrane or ECM [105]. The active signaling molecules act as homodimers, and the mature BMP monomers contain seven cysteines, six of which form intramolecular disulfide bonds. The remaining seventh cysteine residue is involved in the dimerization with another BMP monomer through a covalent disulfide bond, resulting in a biologically active dimeric ligand for BMP receptor activation [21,110]. BMPs are dimeric molecules that display sites for N- and O-glycosylation, which increase the stability and half-life of the protein in the body as well as determine the specificity of receptor coupling [111,112,113]. In addition, heterodimers in particular experimental settings in vitro and in vivo have been shown to be more potent than the corresponding homodimers (i.e., BMP-2/-5; BMP-2/-6; BMP-2/-7) [114,115,116]. For instance, BMP-7 has been shown to function in development largely as a heterodimer with BMP-2 and BMP-4 [117]. The BMP-2/BMP-7, BMP-4/BMP-7, and BMP-2/BMP-6 heterodimers have increased signaling activity compared with homodimers [22,118,119,120,121,122,123]. This increased bioactivity has been attributed to a relatively higher affinity for type I receptors of BMP-2/BMP-4 and a relatively higher affinity for type II receptors of BMP-6/BMP-7, creating a hybrid signaling molecule with higher total receptor affinity [118].

**Figure 4:**
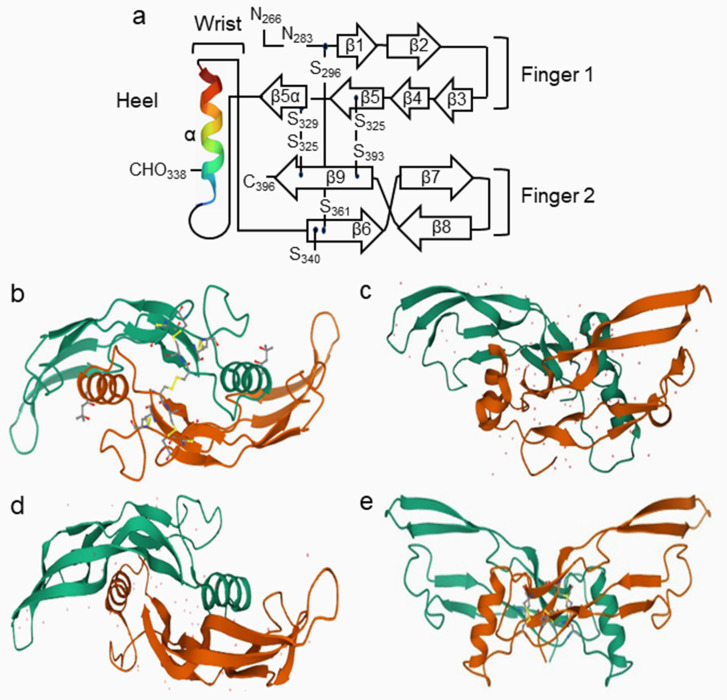
Diagram representing the structure of dimeric BMPs. (**a**) The BMP-2 monomer fold represents the α-helices and β-sheets shown by spiral and arrows, respectively. The BMP-2 cystine knot constitutes the core of the monomer and consists of three disulfide bonds (“S”); two, Cys-325-Cys-393 and Cys-329-Cys-325, form a ring through which the third, Cys-296-Cys-361, passes. The strands of antiparallel β-sheets of β1 to β9, which emit from the knot, form two fingerlike projections. An α-helix, located on the opposite end of the knot, lies perpendicular to the axis of the two fingers, thus forming the heel of the hand. The N terminus corresponds to the thumb of the hand (adapted from Carreira et al., 2014, ref. [21]). (**b**) The dimeric structure of BMP-2. Each monomer is indicated by one color (green and orange), with the disulfide bridges being denoted by projections of the cysteine (yellow) side chains (adapted with permission from Scheufler et al., 1999., ref. [125]). The color figure is available online at https://www.rcsb.org/structure/3BMP, accessed on 15 December 2021. (**c**,**d**) Crystal structure of BMP-3 and BMP-6 in pink and green (adapted with permission from Allendorph et al., 2007, ref. [128]; online at https://www.rcsb.org/structure, accessed on 17 June 2022). 2QCQ and 2QCW. (**e**) Crystal structure of BMP-7 (adapted with permission from Griffith et al., 1996, ref. [124]; online at https://www.rcsb.org/structure/1BMP, accessed on 17 June 2022).

**Figure 5:**
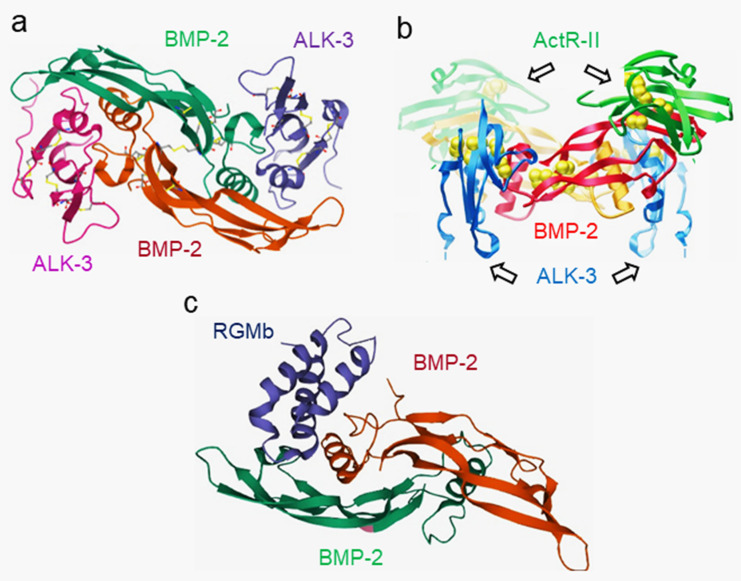
Crystal structure of BMPs and receptors. (**a**) Architecture of the interaction of BMP-2 and ALK-3. Stereoview of the structure of the BMP-2 (green and magenta) and ALK-3 (blue and pink) complex. BCSB.org; PDB ID code 1 REW (adapted with permission from Keller et al., 2004; ref. [132]). (**b**) The ternary complex of BMP-2–ALK-3 ECD–ActRII-ECD displays a butterfly-like conformation. BMP-2 dimer subunits are shown in red and orange; the two ALK-3-ECDs are indicated in light blue and blue, and the ActR-II-ECDs are shown in light green and green. BCSB.org; PDB ID code 2GOO (adapted with permission from Allendorph et al., 2006, ref. [133]). (**c**) The crystal structure of the BMP-2 and RGMb complex. Cartoon representation of the BMP-2 and RGMbND complex. BMP2 is shown in blue and magenta; RGMCND is shown in blue. RCSB.org; PDB ID code 4UHY (adapted with permission from Healey et al., 2015; ref. [134]).

Classically portrayed as a hand, each chain consists of two sets of anti-parallel β-strands forming finger-like extensions that protrude from a central stabilizing “wrist” α-helix. The structure is stabilized by a conserved cystine knot formed by three internal disulfide bonds, similar to other growth factors [22] (Figure 4). Crystal structures of BMP homodimers revealed that the core structures of BMP dimers consist of a “cystine knot” structure and that the overall structure of BMPs has a “wrist and knuckle” or “two bananas” shape [124,125,126,127,128]. Studies of the crystal structures of BMPs have also identified possible binding epitopes of BMPs to specific receptors and antagonists [126,127,128,129,130,131,132,133,134] (Figure 5). 

## 3. BMP Downstream Gene Expression during Tooth Development

### 3.1. Runt-Related Transcription Factor 2 (Runx2)

**Figure 6:**
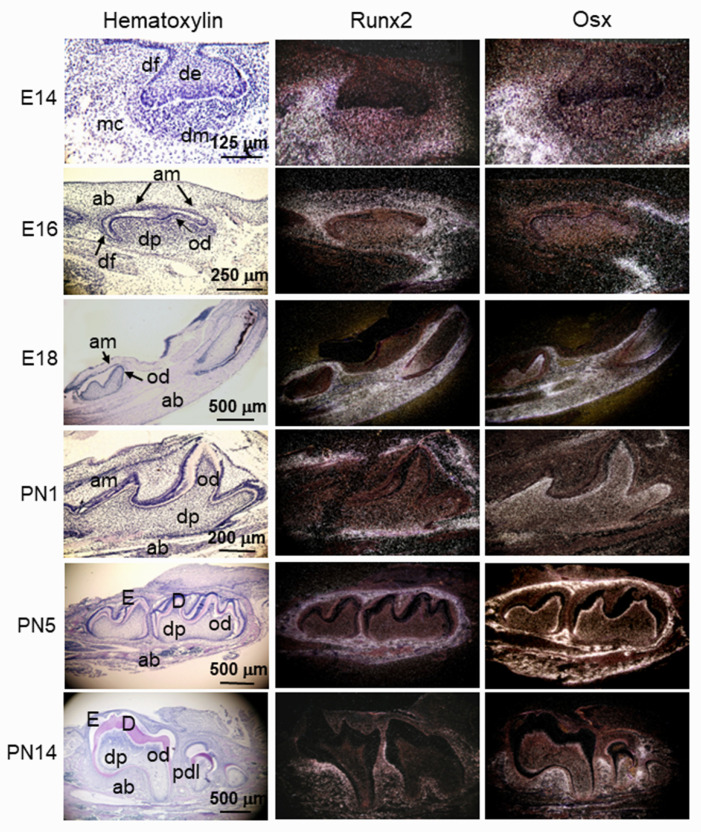
Runx2 and Osx expression patterns in developing teeth. Runx2 mRNA was clearly detected in dental and osteogenic mesenchyme from E14 to E16. Its expression was intense in osteogenic mesenchymal cells, whereas expression was downregulated in odontoblasts, ameloblasts, and dental pulp cells at E18 of the mandibular incisor and first molar. At this stage, Osx expression mostly overlapped with Runx2 expression from E14–E16. At E18, its expression was prominent in ameloblasts, odontoblasts, and dental pulp cells. From PN 1 to PN 14, Runx2 mRNA was detected in osteogenic mesenchyme and remarkedly downregulated in ameloblasts, odontoblasts, and dental pulp cells. In contrast, Osx expression continued to be present in osteogenic mesenchyme, odontoblasts, dental pulp cells, and periodontal ligament cells. ab, alveolar bone; am, ameloblasts; D, dentin; de, dental epithelium; df, dental follicle; dp, dental papilla; E, enamel; E, embryonic day; mc, mesenchymal condensates; od, odontoblasts; pdl, periodontal ligament cells. PN, postnatal day. Photos are partially adapted with permission from Chen et al., 2009, ref. [143].

Runx2 (also designated AML-3, PEBP2αA, CBFA1, Osf2) is a critical transcriptional regulator of odontoblast and osteoblast differentiation. The functions of Runx2 in bone and tooth development are dependent on gene expression at given stages during dentinogenesis and osteogenesis [135,136,137,138,139,140,141]. Runx2 has a unique expression pattern in dental mesenchymal cells from the bud to early bell stages during active epithelial morphogenesis. At the initiation stage of tooth development (E11), an in situ hybridization assay showed that there is no Runx2 expression in any craniofacial tissues. However, at the bud stage (E12–E13), the Runx2 transcript is intense in the mesenchymal condensates of forming bones and teeth in the developing maxillary and mandibular arches [142,143,144]. At the cap stage (E14), the expression of Runx2 appears prominent in the dental papilla, dental follicle, and mesenchyme in osteogenic zones. However, the Runx2 mRNA transcript is barely seen in the dental epithelium. At the bell stage (E16), the Runx2 transcript is expressed in ameloblasts, odontoblasts, dental pulp cells, dental follicles, and differentiating osteogenic mesenchyme. At the late bell stage (E18), Runx2 expression is dramatically downregulated in the ameloblasts, odontoblasts, and dental pulp cells, except for cells near the mesenchyme within the alveolar bones in the developing incisors and molars. Its signal is apparent within osteoblasts in differentiating alveolar bones (Figure 6). On PN 1, Runx2 mRNA is highly expressed within osteoblasts in alveolar bones and dental follicles, which give rise to cementoblasts and PDL cells, but its signal is weakly expressed in ameloblasts, odontoblasts, and dental pulp cells in the developing incisors and molars. On PNs 5 to 14, Runx2 expression patterns are similar to those on PN 1. Notably, Runx2 expression continues to PDL cells, cells within the cementoenamel junction, and roots during tooth root development. Following the eruption of the first molar at PN 28, expression of Runx2 is present in the PDL, cementoblasts, cementocytes, and osteoblastic cells in the alveolar bones [145,146,147]. Therefore, the developmental profiles of Runx2 expression in odontoblasts and osteoblasts appear to differ greatly, suggesting that this gene is differentially regulated in these cells and plays different biological roles in dentinogenesis and osteogenesis [148,149].

Heterozygous mutations of the Runx2 gene cause an autosomal dominant human disease termed cleidocranial dysplasia (CCD; OMIM119600). CCD patients exhibit supernumerary teeth, delayed eruption, malformed roots, and absence of cellular cementum [137,140,150]. The expression of Runx2 was upregulated within odontoblasts at the early stages of mouse tooth development but downregulated in odontoblasts at the later stages of tooth development [143,148]. This indicates that Runx2 plays a dual role in dentinogenesis and tooth-related gene expression. Runx2 upregulated Dspp expression in pre-odontoblastic cells but downregulated Dspp transcription in matured odontoblastic cells [148,151]. Overexpression of Runx2 in mice resulted in dentin defects in the late stages of tooth development and downregulated Dspp gene expression in odontoblasts [152]. This indicates that, unlike bone, the biological functions of Runx2 during dentinogenesis are performed in a spatial–temporal manner. Dlx5 competes with Msx2 and binds to homeodomain response motifs in the Runx2 promoter and Dlx5 significantly upregulates Runx2 transcription, whereas Msx2 suppresses Runx2 expression [153]. Smads are able to interact with Runx2 synergically to promote gene expression [154]. In addition, Smad ubiquitin regulatory factor 1 (Smurf1) interacts with the proline–tyrosine (PY) domain in the COOH-terminus of the Runx2 protein and induces Runx2 protein degradation [155]. Conditional Smad4 KO mice (Dmp1-Cre;Smad4fx/fx) led to dentin defects, with wide predentin, thin dentin, disorganized dentin tubules, and decreased expression of Bsp, Collα1, Dmp1, and Dspp genes exhibited [156]. Canonical Smad signaling enhanced dental mesenchymal cell differentiation and Dspp expression via the action of Dlx5, Msx2, Osx, Runx2, Smads, and other compounds [92,157,158,159,160]. 

### 3.2. Osterix (Osx or Sp7) 

Osx is a zinc finger-containing transcriptional factor distinctly expressed in all developing bones and teeth [143,161]. It is important for odontoblast and osteoblast differentiation. At the bud stage (E12) of mouse tooth development, the antisense Osx probe is intense in the mesenchymal condensates of forming bones and teeth in the developing maxillary and mandibular arches. At the cap stage (E14), Osx mRNA is expressed in mesenchymal cells in the alveolar bone, dental papilla, and dental follicle, while Osx expression is barely detected in the dental epithelium. At the bell stage (E16–E18), Osx transcripts are present in differentiating osteogenic mesenchyme, ameloblasts, odontoblasts, and dental pulp. On PNs 1 to 14, Osx expression remains abundant in odontoblasts, dental pulp cells, and osteoblasts in alveolar bone, but its expression is weakly detected in secretory ameloblasts. Furthermore, Osx expression is also seen in PDL cells, cementoenamel junction, and HERS [143,146,162,163,164] (Figure 6). During dentin regeneration and orthodontic tooth movement, expression levels of Osx are increased in odontoblasts, osteoblasts, and cementoblasts in experimental groups compared to control groups [147,165,166]. From this, it is implied that Osx participates in dentin regeneration and root formation.

Mutations of the Osx gene in humans and mice are associated with defects in both teeth and bones [161,167,168,169,170,171]. In Osx null cells, tooth roots were short with a cuboid-like odontoblast layer containing few dentin tubules [168]. Further studies showed that Osx induces dental mesenchymal differentiation and mineralization and expression of Alp, Col1, Dmp1, and Dspp genes [37,143,172], while the expression of Dspp, Dmp1, and Collα1 was also significantly declined in Osx cKO mice, which may contribute to the resultant promotion of cell proliferation and differentiation [168,173,174]. Further experiments demonstrated that Osx directly binds to the Dspp gene promoter and that it upregulated their transcriptions in dental papilla mesenchymal cells [37,175]. The effect of Osx on gene expression is regulated by either BMP–Smad–Runx2-dependent or BMP–Smad–Runx2-independent signaling pathways [174,176,177]. BMP-mediated Dlx5, Msx2, and Runx2 facilitated their downstream gene expression via Osx [178,179]. Tao et al. reported that Klf4 promotes odontoblast differentiation and Dmp1 expression through Osx [180]. Furthermore, Nfic induced expression of Dmp1 and Dspp genes, as well as promoted odontogenesis via regulating Osx expression [168,174,181]. 

### 3.3. Distal-Less (Dlx) Homeobox Gene 3 (Dlx3)

**Figure 7:**
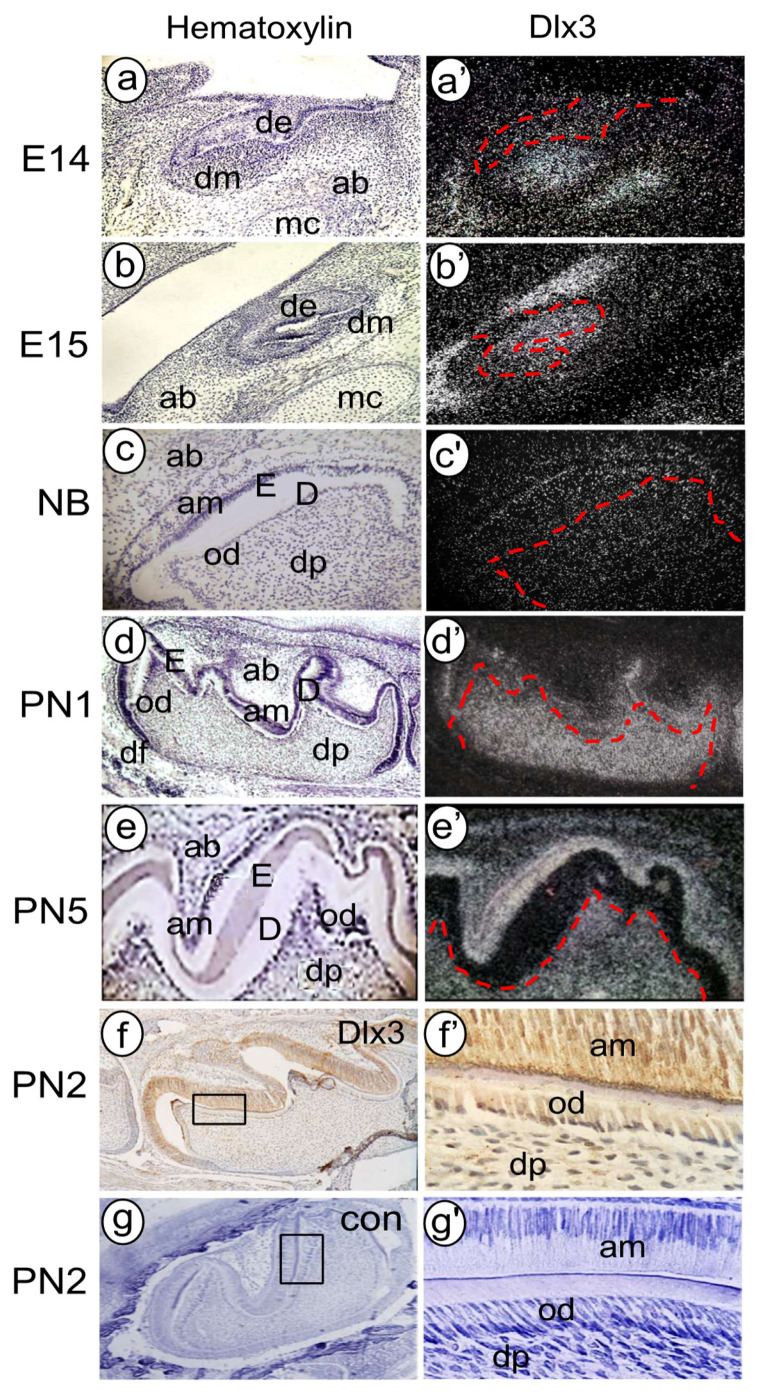
Expression of Dlx3 during mouse tooth development. (**a**–**e**)**.** Bright images. (**a’**–**e’**) In situ hybridization for Dlx3 expression during mouse tooth formation. At the cap stage (E14–E15), the anti-Dlx3 probe is strongly present in the dental mesenchyme and osteogenetic mesenchyme developing alveolar bone and is moderately visible in the dental epithelium and Meckel’s cartilage. At new birth (NB) and PNs 1 and 5, expression of Dlx3 can be detected in ameloblasts, odontoblasts, dental papilla, and dental follicles, as well as in the mesenchyme in the alveolar bone. At PN 1, Dlx3 expression is more intense in ameloblasts, odontoblasts, and dental papilla than in developing alveolar bone mesenchymal cells. (**f**,**f’**). Tooth tissues were immunostained with anti-Dlx3 antibody, showing that the Dlx3 expression is apparent in ameloblasts, dental pulp cells, and odontoblasts at PN 2 of mouse tooth development. (**f’**) shows higher a magnification of the box in (**f**). The tissue sections were stained with anti-IgG serum as the negative control. (**g’**) shows a higher magnification of the box in (**g**). ab, alveolar bone; am, ameloblasts; con, control; D, dentin; de, dental epithelium; dm, dental mesenchyme; df, dental follicle; dp, dental papilla; E, enamel; E, embryonic day; mc, Meckel’s cartilage; od, odontoblasts; PN, postnatal day. The data are unpublished.

Dlx3, one of the Dlx family members, is required for epithelium, hair, bone, tooth, and placental development [182,183]. At the tooth initiation stage (E9.5–E10.5), Dlx3 expression is visualized in the mesenchyme of the first branchial arch and the mandibular epithelium in the most mesial region of the mandibular arch [184,185]. At the bud stage (E12.5–E13.5), Dlx3 is expressed in the dental lamina of lower incisors and lower molars, and the labial enamel epithelium of upper molars. Dlx3 is also detected in mesenchyme adjacent to the dental lamina [183,185,186,187]. After the dental lamina forms, dental epithelial cells invaginate into the dental mesenchyme to form an epithelial bud and subsequently develop into the cap- and bell-stage-shaped tooth germs. The spatial–temporal expression patterns of Dlx3 are highly evident in the dental epithelium, pre-ameloblasts, ameloblasts, dental follicles, and weakly evident in dental papilla and pre-odontoblasts through the cap to bell stages [183,185,186,187,188]. Dlx3 is expressed in the dental follicle mesenchyme, which is involved in the development of PDL and the cementum. After birth, Dlx3 continues to show strong expression in ameloblasts and weak expression in odontoblasts. In addition to the expression of Dlx3 in ameloblasts and odontoblasts during dental morphogenesis and histodifferentiation, Dlx3 expression is also present in chondrocytes and osteoblasts [183,189] (Figure 7).

Dlx3 mutations are associated with a rare autosomal-dominant disease termed tricho–dento–osseous syndrome (TDO; OMIM190320), which is an autosomal-dominant disorder [190,191,192]. The clinical features of TDO are curly hair, increased thickness and density of bone, enamel hypoplasia and hypomaturation, dentin hypoplasia, and taurodontism [193,194,195]. Dlx3 is expressed in odontoblasts during tooth development and formation [37,196,197]. Dlx3 mutant mice exhibited dentin deficiency with delayed odontoblast differentiation and reduced expression of Dspp, Runx2, and Wnt10A genes [196,197]. Dlx3 binds to its responsible elements in the mouse Dspp promoter and activated Dspp gene transcription [37,197,198]. BMP-mediated Dlx3 induces Dspp expression and dental mesenchymal differentiation and mineralization [37,199].

### 3.4. Msh (Muscle Segment Homeobox) Drosophila Homolog 1 (Msx1) and Msx2

Msx1 (also designated homeobox 7 (Hox7)) is involved in tooth development and formation. Through tooth development, Msx1 transcripts are highly restricted to the dental mesenchyme [30,200,201,202]. At the tooth initiation stage (E11), expression of Msx1 is broadly localized to the mesenchyme surrounding the thickened epithelium in the maxillary processes and the mesenchyme at the tip of the mandibular arch [200]. By E12, expression of Msx1 is restricted to the mesenchyme immediately surrounding the developing tooth germs in the maxillary and mandibular processes. Msx1 expression in the mesenchyme of the dental papilla and dental follicle is maximal at the cap stage of mouse tooth development and progressively declines in the bell stage prior to the differentiation of odontoblasts and ameloblasts [200]. Through the cap stage, the Msx1 transcript is intense in the dental papilla and dental follicle and osteoblasts in future alveolar bones. However, the labeling of Msx1 is not visible in the enamel organ and other epithelia in all tooth germs [200]. At the bell stage, Msx1 expression continues in the dental papillae and dental follicle as well as osteoblasts, but its signal decreases in intensity in the dental papilla, though it is still higher than in the oral mesenchyme surrounding the tooth germ [201]. During root development, Msx1 expression is detected in the dental pulp and weakly seen within osteoblasts in the alveolar bone and PDL cells [30]. 

MSXs are homeobox genes and constitute a large family of transcription factors. Mutations of MSX genes result in cleft lip, cleft palate, and tooth agenesis. Patients with MSX1 mutations exhibited teeth with oligodontia [203,204]. Mutations of MSX1 in humans and mice cause tooth deficiency [203,205,206,207]. BMP–Smad signals induced tooth development via MSX1. MSX1 induced human DPSC proliferation and differentiation as well as expression of BSP and DSPP genes in human DPSCs [208,209]. However, Feng et al. reported that MSX1 promotes proliferation and prevents the differentiation of dental mesenchymal cells by the inhibition of BMP-2 and BMP-4 expression and downregulation of DSPP, DMP1, Runx2, OC, and Collα1 gene expression [210,211]. Of note, an atypical canonical BMP signaling pathway in a Smad1/5/8-dependent, but Smad4-independent manner in the dental mesenchyme during odontogenesis upregulated Msx1 expression via binding of pSmad1/5/8 proteins in Msx1 gene promoter [212,213]. 

At the tooth initiation stage of mouse tooth development (E9–E11), Msx2 expression is present in the neural crest-derived mesenchyme of branchial arches and maxillary and mandibular processes. Msx2 transcript in the oral epithelium is first evident at the sites of the dental placodes [214,215,216,217]. By E10.5, Msx2 mRNA is not detectable in the epithelium, but by Ell.5 Msx2 expression is visible in dental epithelial cells, immediately above its previous area of the mesenchymal expression. At the bud stage, in situ hybridization anti-Msx2 probe is evident in external enamel epithelium and the mesenchymal cells of the dental papilla and dental follicle immediately surrounding the invaginating dental epithelium. At the cap stage, Msx2 transcript is visible in the dense epithelium of the enamel knot adjacent to the internal enamel epithelium and septum and associated external enamel epithelium. Additionally, its signal is apparent in the layer of dental papilla mesenchyme cells and dental follicles. When the transitory enamel knot, septum, and navel disappear at the bell stage, Msx2 is expressed throughout the internal enamel epithelium, the external enamel epithelium, ameloblasts, and the cervical loop. Low levels of Msx2 expression are also evident in the cellular condensations of the stellate reticulum in the cervical loops. Expression of Msx2 also appears in the dental papilla mesenchyme, pre-odontoblasts, and odontoblasts as well as dental follicle mesenchyme [214,215,216,217]. In PNs, Msx2 expression is detected in the dental papillae cells, odontoblasts, and ameloblasts, but barely in osteoblasts in the alveolar bone. Msx2 expression is also seen in the cervical loop and in the stratum intermedium [218]. During mouse root development, Msx2 is expressed in HERS besides dental pulp and dental follicles, the adult periodontium, inner enamel epithelium, and outer enamel epithelium [219,220]. 

MSX2 gene mutation in humans caused Boston-type craniosynostosis characterized by the premature fusion of skull bones together with certain orofacial bone abnormalities [221,222]. Contrarily, haploinsufficiency of MSX2 causes midline cranial defects reflected in the occurrence of wide-open fontanels in the skull vault [223]. In Msx2 null mice, tooth root eruption was delayed, dentin deposition was affected, and dental pulp chambers were larger in size than in wild-type mice. The odontoblast row and odontoblastic layer were highly disorganized, and abnormal polarization ended with cellular inclusion within the pre-dentin and dentin matrix in Msx2 mutant mice. Different layers were visible in the dentin thickness of the mutant mice [224]. Deletion of Msx2 in mice reduced the expression of Alp, Col1, Dspp, Oc, Runx2, and Bmp-4 genes in odontoblasts [224,225]. Msx2 competed with Dlx5 in homeodomain response elements (ATTA) in the Dspp regulatory region and repressed Dspp gene transcription in C2C12 cells. By contrast, Dlx5 upregulated Dspp expression [157,226]. On the other hand, Msx2 interacts with CCAAT/enhancer-binding protein (C/EBP) and binds to its binding site(s) in the mouse amelogenin gene promoter, repressing amelogenin transcription [227]. MSX2 and BMP-4 have mutuality between one another [217,228,229], and BMP induces Msx2 expression through Smad signaling [228,230,231,232]. In addition, BMP-2-mediated Msx2 stimulated Osx gene expression in Runx2-deficienct mesenchymal cells [179]. 

### 3.5. Paired Box 9 (Pax9)

Pax9 is a transcription factor that plays an important role in the mechanisms of ontogeny in all classes of vertebrates. At the initiation stage of mouse tooth development, Pax9 transcript is low in prospective incisor mesenchyme and positive prospective molars as well as mesenchyme in maxillary and mandibular arches [218,233,234]. At the cap stage (E14.5), Pax9 expression is only detected in mesenchymal cells in incisors and molars but is visible in both dental epithelium and mesenchyme in the incisors and premolars in human 12-week-old embryos, which expression pattern is different from that in mice [235]. At the bell stage (E16.5), the Pax9 signal is apparent in the mesenchyme of all teeth [234]. 

Pax9 plays a role in the development of human dentition. Mutations of Pax9 in humans cause hypodontia, including abnormal incisor and canine shapes and missing premolars and permanent molars [233,236,237,238]. Pax9 is one of the most widely studied genes participating in odontogenesis [235,239,240]. Tooth development is arrested at the bud stage in Pax9-deficient mice and tooth germs are small in Pax9 null mice compared to controls, indicating that Pax9 is required for tooth development at or before this stage [233,234,241]. The arrested tooth development in Pax9 null mice was partially rescued by BMP-4, but Pax9 also upregulated BMP-4 and Msx1 gene expression [241,242,243,244]. Overexpression of Pax9 enhanced the expression of Dmp1 and Msx1 in the induced pluripotent stem cells (iPSCs) derived from neural crest-like cells (iNCLCs), but BMP-4 also upregulated the expression of Dmp1, Dspp, and Msx1 genes in iNCLCs. Co-expression of Pax9 and BMP-4 synergistically promoted the expression of Dmp1 and Pax9 genes in iNCLCs [245]. BMP–Smad signaling controls odontogenesis and Dspp expression via Pax9 [246].

### 3.6. Dentin Matrix Protein 1 (DMP1)

**Figure 8:**
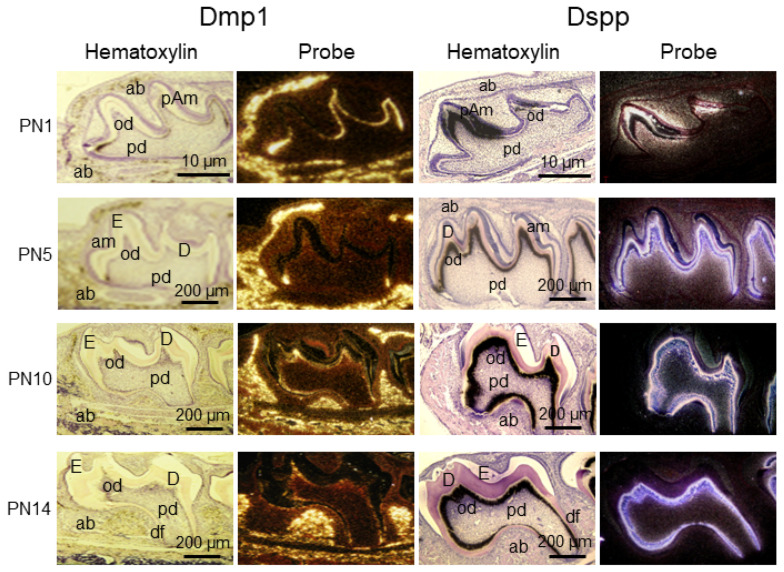
Dmp1 and Dspp expression patterns in developing teeth. By in situ hybridization assay, Dmp1 mRNA transcription was clearly detected in odontoblasts near the dental crown and root of the mouse tooth and osteoblasts in alveolar bones by PN 1, while by PN 5, Dmp1 expression was downregulated in odontoblasts and only appeared in odontoblasts located at the dental root but was highly intense in the alveolar bone. By PN 10–14, Dmp1 transcription was weakly visible in odontoblasts, but still highly expressed in osteoblasts in alveolar bones. By PN 1, Dspp transcripts were evident in odontoblasts, dental papilla, and pre-ameloblasts in the mouse mandibular molars. By PN 5, Dspp mRNA was strongly expressed in odontoblasts and weakly seen in ameloblasts and dental pulp cells. From PN 10 to 14, Dspp mRNA was mainly expressed in odontoblasts and weakly in dental pulp cells. Dspp expression was barely detected in osteoblasts in alveolar bone from PN 1 to PN 14 during mouse tooth development. ab, alveolar bone; am, ameloblasts; D, dentin; df, dental follicle; dp, dental papilla; E, enamel; od, odontoblasts; mc, Meckel’s cartilage; pAm, pre-ameloblasts; pd, dental pulp; PN, postnatal day. The photos are partially adapted with permission from Chen et al., 2009, ref. [143].

DMP1 is a non-collagenous protein originally identified in dentin and belongs to the family of small integrin-binding ligand N-linked glycoproteins (SIBLINGs), which are essential for the proper development and formation of hard tissues, such as teeth and bones [247,248]. In mouse tooth development, at the bud stage (E13), no signal for Dmp1 is detectable within either tooth ectoderm or mesenchyme, but Dmp1 transcripts are visible in the first mouse mandibular molar at E13 and E14 by RT-qPCR assay [249,250]. At the cap stage (E15), Dmp1 mRNA is intense in newly differentiated osteoblasts within the forming alveolar bone, while a weak signal of Dmp1 is seen in the enamel organ and dental papilla. At the bell stage, Dmp1 transcription is evident in the inner dental epithelium of the enamel organ and newly differentiated or young odontoblasts at the mesiobuccal cusp tip of the first mouse molar. On PNs, an antisense Dmp1 riboprobe is accumulated in polarized odontoblasts and faintly hybridized to preameloblasts at PN 1. Dmp1 mRNA is notably present in osteoblasts (Figure 8). At PN 6, Dmp1 mRNA appears clearly in differentiated odontoblasts, dentinal tubules, and predentin, and is significantly downregulated in mature odontoblasts as well as upregulated in ameloblasts at the maturated stage of amelogenesis. At the same stage, osteoblasts in the alveolar bone maintain high levels of Dmp1 mRNA expression. In erupted molars at PN 26, Dmp1 is expressed in odontoblasts, while a more intense signal is observed in osteoblasts and osteocytes of inter-radicular bone [249,250,251,252,253]. During root development, Dmp1 mRNA is detected in cementoblasts and cementocytes in the cementum, besides odontoblasts, dentinal tubules, and osteoblasts [254]. 

DMP1 mutations in humans and animals are associated with tooth and bone deficiency [255,256,257,258,259]. Patients with DMP1 mutations displayed normal tooth growth and shape, but their deciduous and permanent teeth exhibited enlarged pulp cavities, thin dentin and enamel, as well as the absence of lower third molars and hypomineralization compared to healthy controls [258]. In Dmp1 null mice, as analyzed from PN 3 to 1 year of age, tooth morphology is similar to dentinogenesis imperfecta type III (DGI-III) in humans. Teeth in the mutant mice displayed enlarged dental pulp chambers, wide predentin, thin dentin and altered dentin matrix structure, and hypomineralization. Expression of Dspp, one of the important non-collagenous proteins in dentin, was decreased in odontoblasts, whereas biglycan, one of the small leucine-rich proteoglycan family members, was increased in odontoblasts of the null mice compared to the controls [255]. 

### 3.7. Dentin Sialophosphoprotein (DSPP)

Both DMP1 and DSPP are phosphorylated matrix proteins [247,248], but DSPP has distinct expression patterns and is highly distributed within pre-ameloblasts and odontoblasts during tooth development. At the bud stage (E13) of molar development, an in situ hybridization assay showed that no notable signal of both Dmp1 and Dspp was detected in either the ectodermal or mesenchymal components of the mouse tooth organ and surrounding tissues. However, while the Dmp1 transcript was amplified from the first mandibular molar by RT-qPCR analysis, there was no Dspp signal [143,249]. At the cap stage (E15), a high level of Dmp1 mRNA appears in newly differentiated osteoblasts in the alveolar bone, whereas no signal of Dspp is evident in osteoblasts within the same serial section. At the bell stage (E16–E17), Dspp transcription is first visible in young and mature odontoblasts and pre-ameloblasts and is weakly seen in dental pulp cells [143,249,260,261]. On PN 1, a high level of Dspp expression is mainly restricted to the tooth organ and progresses along a developmental gradient in odontoblasts, dental pulp cells, and preameloblasts [262,263] (Figure 8). By PN 6 and later stages of tooth formation, Dspp expression retains in odontoblasts through all phases of primary dentinogenesis, and transient Dspp transcripts are present in early secretory ameloblasts at the growing end of the molar organ [264]. Besides Dspp expression in ameloblasts, odontoblasts, dental pulp cells, and dentin matrix, low expression levels of Dspp are found in osteoblasts, osteocytes, cementoblasts, PDL cells, and non-mineralized tissues [251,265,266,267,268]. 

DSPP mutations in humans are associated with dentinogenesis imperfecta type II (DGI-II, OMIM 125490) and DGI-III (OMIM 125500), as well as dentin dysplasia type II (DD-II, OMIM 125420) and DD-I (MIM 125400) [269,270,271,272,273]. These hereditary dentin diseases are the most common dentin genetic diseases. DGI-II is characterized by bulbous crown shape, opalescent discolored dentition, and pulpal calcification, as well as impaired odontoblast cell differentiation and delayed conversion of predentine to dentine. DGI-III was originally regarded as a Brandywine isolate [274], along with a severe form of DGI-II with multiple dental pulp exposures and shell-like teeth. In DD-I, teeth are normal in shape and form as well as consistent in deciduous and permanent dentitions. In some cases, teeth may show a slightly amber discoloration. However, the roots are short, and pulp obliteration causes a crescent-shaped pulpal remnant in the permanent dentition and a total pulpal obliteration in the deciduous dentition. Teeth in DD-II patients are similar to the deciduous dentition in DGI-II, but tooth discoloration is minimal and dental pulp chambers are thistle-tube-shaped with pulp stones in the permanent dentition.

Homozygous null mice (Dspp−/−) analyzed from 2–3 months after birth showed tooth deficiencies similar to those seen in patients suffering from DGI and DD, with enlarged pulp chambers, a wide predentine zone, thin dentin, dentin hypomineralization, and dental pulp exposure. Expression of biglycan and decorin in PNs 6, 15 and 1-year-old teeth in mutant mice was increased compared to wild-type mice [275]. 

## 4. BMP Signaling during Tooth Development

**Figure 9:**
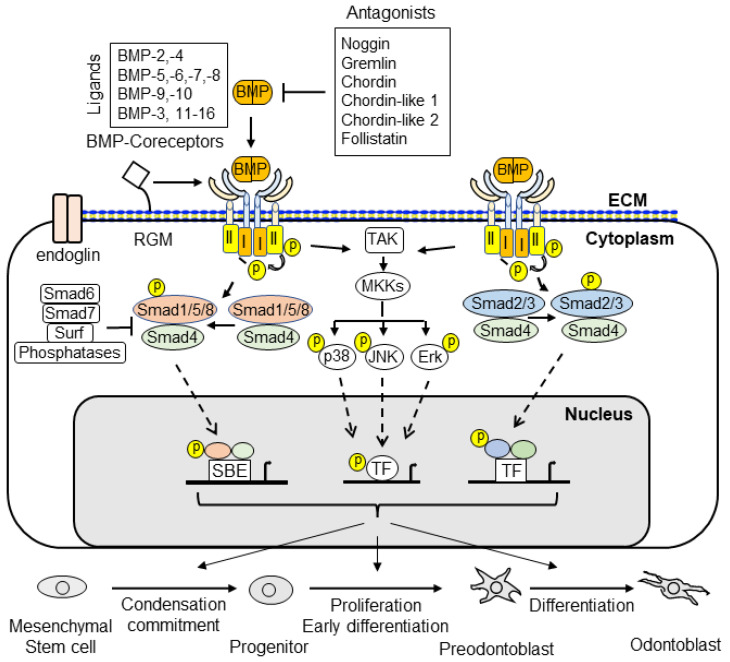
BMP ligands, receptors, and interacting receptors. BMP signal transduction involves a number of ligands, type I and type II serine/threonine kinase receptors, and coreceptors, which regulate the activation of intracellular mediators in interactions with extracellular stimuli. BMPs are classified into several subfamilies according to sequence homology. The BMP signaling pathways. Canonical Smad signaling executes its function through Smad proteins. BMPs interact with the type I and type II receptors to form a heterotetrameric complex. Complex formation and ligand binding can be potentiated by a coreceptor, that is, endoglin, betaglycan, or repulsive guidance molecule (RGM). Upon phosphorylation by the type II receptor, the type I receptor recruits and phosphorylates pathway-specific R Smads (Smad-1, Smad-5 and Smad-8, and Smad-2 and Smad-3), which can form trimers with Smad-4 and translocate to the nucleus. Smads have intrinsic DNA-binding activity and can regulate gene expression by recruitment of chromatin-remodeling machinery and integration with tissue-specific transcription factors. Non-canonical Smad signaling, through phosphorylated TAK (pTAK). Ligand binding induces the formation of the receptor complex. BmpR-I is linked to the TAK1/TAB1 complex through the X-linked inhibitor of apoptosis (XIAP) to phosphorylate and activate the downstream mitogen-activated protein kinases (MAPKs), including p38, ERK1/2, and JNK. Consequently, activation of these kinases leads to their translocation into the nucleus where they phosphorylate and activate and regulate the transcription of downstream target genes. Canonical Smad signaling is intracellularly inhibited by inhibitory Smads, that is, Smad-6 and/or Smad-7, and E3 ubiquitin ligases, such as Smurf1 or Smurf2, whose expression provides the cell with a negative feedback mechanism. The pathway can be antagonized by many mechanisms, including neutralization of ligands by secreted traps, such as noggin, follistatin, gremlin, chordin, chordin-like; secretion of latent ligands bound to their propeptides; or via titration of receptors by nonsignaling ligands, such as BMP-3. ACVR, activin receptor; ACVR2, activin type-2 receptor; ALK, activin receptor-like kinase; AMH, anti-Müllerian hormone; AMHR2, AMH receptor; BMP, bone morphogenetic protein; BmpR, bone morphogenetic protein receptor; ERK, mitogen-activated protein kinase; GDF, growth/differentiation factor; JNK, c-Jun N-terminal kinase; MAPK, mitogen-activated protein kinase; p38, p38 mitogen-activated protein kinases; RGM, repulsive guidance molecule; SBE, Smad binding element; TAK, TGF-activated kinase; TF, transcriptional factor; TGF, transforming growth factor; TGFBR, TGF-β receptor; XIAP, E3 ubiquitin-protein ligase XIAP. The figure is partially adapted with permission from Miyazone et al., 2005, ref. [279].

**Figure 10:**
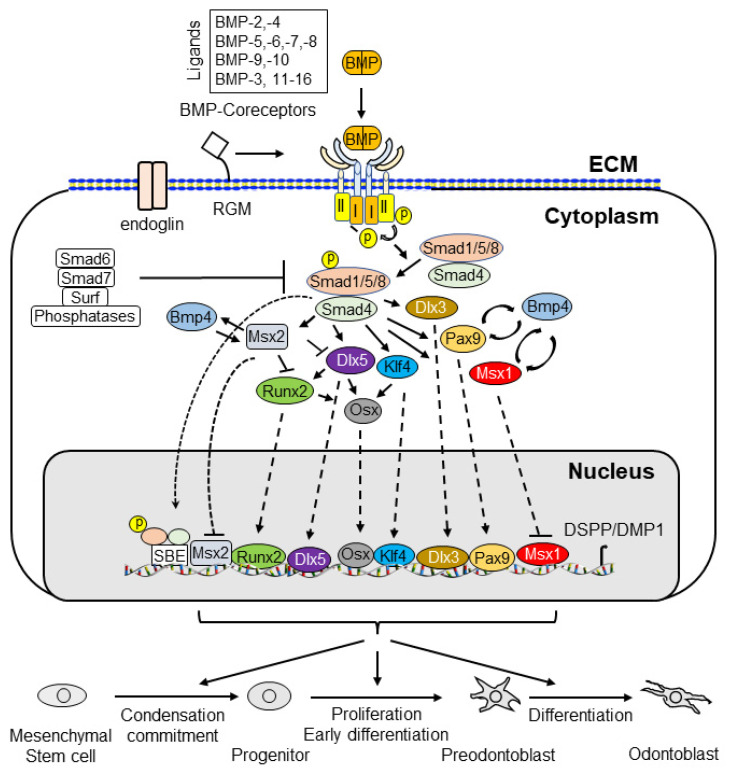
Smad-dependent signaling in dentin formation. BMPs bind to receptor type II (R-II) and receptor type I (R-I) before the signaling transduces to their Smad1/5/8. Activated Smad1/5/8 interacts with Smad4, forming a complex which translocates into the nucleus where they interact with other transcription factors to trigger target gene expression. Consequently, Smad-dependent signaling enhances dental mesenchymal cell differentiation and expression of DSPP and DMP1 genes via the action of Smads, Dlx3, Dlx5, Klf4, Msx1, Msx2, Osx, Pax9, and Runx2, and other compounds during dentinogenesis. Smad6 binds the type I BMP receptor and prevents Smad1/5/8 from being activated. Additionally, Smurf1 interacts with the Runx2 protein and induces Runx2 protein degradation. The figure is partially adapted with permission from Wu et al., 2016, ref. [291].

**Figure 11:**
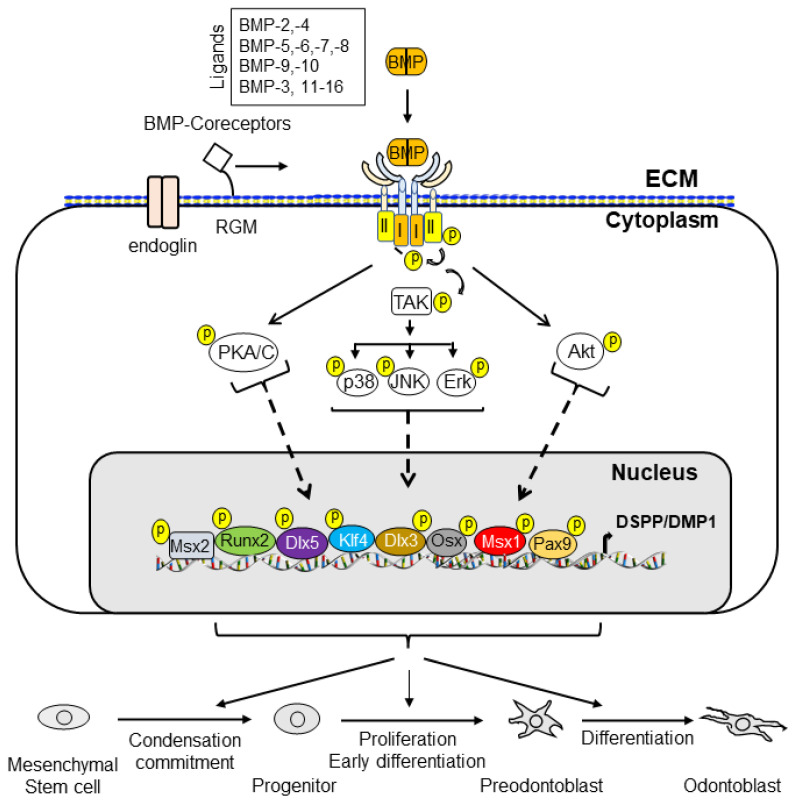
Smad-independent signaling pathways. BMP signal transduction involves a number of ligands, type I and type II serine/threonine kinase receptors and coreceptors, which regulate the activation of intracellular mediators in interactions with extracellular stimuli. Smad-independent signaling through phosphorylated TAK (pTAK), protein kinases A and C (PKA/C), and PI3K/Akt. Ligand binding induces the formation of the receptor complex. BMP receptors linked to the TAK1/TAB1 phosphorylate and activate the downstream MAPKs, including p38, ERK1/2, and JNK, respectively. Consequently, activation of these kinases leads to the phosphorylation of several transcriptional factors, activating the transcription of downstream target genes. In addition, PI3K/Akt and PKA/C are phosphorylated by BMP receptors. Then activated PI3K/Akt and PKA/C phosphorylate their downstream transcription factors. These activated transcription factors bind to their binding responsible elements in their target gene regulatory regions and upregulate the expression of DSPP and DMP1 genes and induce dental mesenchymal cell differentiation and dentin formation. Akt, (v-Akt murine thymoma viral oncogene)/PKB (protein kinase B); ERKs, extracellular signal-regulated kinases; JNKs, c-Jun NH2-terminal kinases; PI3K, phosphoinositol 3-Kinase; PKA, protein kinase A; PKC, protein kinase C. The figure is partially adapted with permission from Wu et al., 2016, ref. [291].

The BMP ligands, as dimers, bind to two distinct receptors, type I serine/threonine kinase and type II receptors, which are necessary for signal transduction [276,277,278,279]. The serine/threonine kinase domains of type II receptors are constitutively active and phosphorylate Gly-Ser (GS) domains in the type I receptors upon ligand binding, leading to the activation of type I receptor kinases (Figure 9). BMPs bind to three distinct type II receptors, for instance, BMP type II receptor (BmpR-II), activin type II receptor (ActR-IIA or AcvR-2A), and activin type II B receptors (ActR-IIB or AcvR-2B), while BMPs bind to the type I receptors, ALK-1/-2/-3/-6 [105,279,280,281,282]. The specificities of BMP binding to type receptors depend on the identities of the interacting type II receptors and cell types [283] (Figure 2). BMPs display a different affinity for BMP type I receptors: BMP-2 and BMP-4 bind to ALK-3 and ALK-6 [22,132,284] (Figure 2 and Figure 5), while BMP-6 and BMP-7 bind strongly to ALK-2 and weakly to ALK-6 [285]. BMP-14 preferentially binds to ALK-6 [286]. BMP-9 and BMP-10 bind to ALK-1 and ALK-2 [126,287] (Figure 2). Some BMP type I receptors are shared by certain members of the TGF-β family members. TGF-β binds to ALK-5 (TGFβRI) as well as ALK-1 [282]. In contrast to other BMP family members, BMP-3 and BMP-11, as well as BMP-15 and GDF-8, have been described to activate TGF-β and activin type I receptors ALK-4, ALK-5, and ALK-7, respectively (Figure 2), leading to activation of Smad-2 and Smad-3 [22,288,289] (Figure 2). Thus, BMP-3, BMP-11 and BMP-15 appear to activate signals similar to TGF-β/activins, but distinct from those induced by other BMPs [22]. BMP-3 has been shown to bind to ActR-IIB, which transduces ActR-IIB-Smad-2/-3 signaling to suppress the other BMPs [290,291]. BMP signaling regulates downstream gene expressions through either Smad-dependent or Smad-independent pathways (Figure 9). Following heterodimerization, type I receptors are phosphorylated by type II receptors and subsequently activate the receptor-regulated Smad-1/5/8 through phosphorylation [292,293]. The phosphorylated Smad-1/5/8 heterodimers form a complex with the only common mediator Smad-4 (Co-Smad-4). Following nuclear translocation of the Smad-1/5/8/Co-Smad-4 complex, BMP target gene expression is induced (Figure 10) [292]. On the other hand, BMPs also activate Smad-independent signaling pathways, such as mitogen-activated protein kinases (MAPKs), including extracellular signal-regulated kinases (ERKs), p38MAPKs, c-Jun amino-terminal kinase (JNK), phosphoinositol-3 kinase (PI3K), v-Akt murine thymoma viral oncogene (Akt)/PKB (protein kinase-B), and small GTPases (Figure 11) [294]. Thus, these Smad-independent pathways cooperate with Smad-dependent pathways to regulate various cellular responses. Expression patterns of BMP ligands and BMP receptor types I and II, including BMP-2/-3/-4/-5/-6/-7, ALK-2/-3/-5/-6, and BMPR-II, are observed in tooth tissues at the given stages during tooth development [25,26,30,31,64,75,94]. 

Ligand binding induces the formation of hetero-tetrameric receptor complexes consisting of the type I and type II receptors [24,133]. Tight regulation of signaling intensity, duration, specificity, and diversity is further achieved by the interaction of this receptor complex with co-receptors, e.g., endoglin, betaglycan, repulsive guidance molecules (RGMs, including RGMa, RGMb or DRAGON, and RGMc or Hemojuvelin), neogenin, TrkC, ROR2, MuSK, neuropilins, BAMBI, or integrins [295,296,297]. Co-receptors do not produce a signal directly, but rather promote ligand docking on the cell surface, regulating specificity and likely controlling signaling kinetics [297,298]. Recent reviews have highlighted aspects of co-receptor biology and have been linked to BMP signaling modulation [297]. 

### 4.1. Smad-Dependent Signaling in Dentin Development and Formation

BMPs, their receptors, and Smads have strong odontogenic capacity. BMP-2, -4, and -7 are involved in odontogenesis. These BMPs have a strong odontogenic capability to induce DPSC/progenitor cell differentiation into odontoblasts and stimulate tooth-related gene expression. BMPs bind to their receptors and form the complex, leading to phosphorylation of Smad1/5/8. The phosphorylated Smad1/5/8 interacts with Smad4, and the phosphorylated Smad1/5/8-Smad4 complex translocates into the nucleus from the cytoplasm and stimulates their downstream gene expression (Figure 10). Loss of Bmp-2, Bmp-4, Bmp-7, and Bmp-9 causes severe impairment of dentinogenesis, enlarged dental pulp cavity, wide predentin, thin dentin, tooth attrition, delayed root eruption, and impairment of odontoblast differentiation [33,34,36,37,38,43,44,46,47,299,300,301,302]. Using bioinformatics software, this study showed that the Bmp-2 signal regulates cell differentiation, transcriptional regulation, and developmentally relevant signaling pathways [303]. Teeth in mice lacking Bmp-2, Bmp-4, and Bmp-9 showed reduced tooth-related gene expression, including phosphorylated Smad1/5/8, Alp, Collα1, Dlx3, Dmp1, Dspp, Osx, and Runx2 [32,36,37,38,39,46,47,302]. Both DSPP and DMP1 are important markers for dentin development and formation. Mutations of DSPP and DMP1 genes result in dentin defects [225,258,269,270,271,275]. Teeth of Alk-3 cKO mice displayed dentin deficiency and reduced expression of phosphorylated Samd1/5/8, Klf4, Osx, and Dspp [92,246]. The molecular mechanisms of BMP–Smad signaling pathways in dentinogenesis via several transcription factors have been shown (Figure 10). These include Dlx3, Klf4, Msx1, Msx2, Osx, Pax9, Runx2, and others. Kruppel-like factor 4 (Klf4) is one of the Klf family members, which are evolutionarily conserved zinc finger-containing transcription factors. The expression of Klf4 is specifically detected in odontoblasts and ameloblasts during mouse tooth development and formation [304]. Klf4 cKO mice (Wnt1-Cre;Klf4fx/fx) showed dentin defects and impairment of odontoblast differentiation as well as reduced expression of Osx and Dmp1 gene expression in odontoblasts [180]. Klf4 upregulated expression of Dmp1, Dspp, and Osx genes via binding to Klf binding elements in mouse Dmp1, Dspp, and Osx promoters, therefore controlling dentinogenesis [175,180]. In addition to BMP–Smad signaling pathways, other signaling pathways also regulate odontoblastic differentiation and dentin formation, as well as the expression of Dmp1 and Dspp genes [246,305,306,307,308,309]. 

### 4.2. Smad-Independent Signaling in Dentin Development and Formation 

The Smad-dependent signaling pathway has well been established. However, Smad-independent signaling pathway during tooth development and formation also plays an important role in dentinogenesis. MAPKs, including major kinases, ERK, JNK, and p38, as well as PI3K/Akt and protein kinase A and C pathways, are involved in BMP–Smad-independent signaling in odontogenesis (Figure 11). BMP-mediated MAPKs phosphorylate transcription factors of Dlx3, Klf4, Osx, Pax9, and Runx2, leading to phosphorylated protein stabilities, nuclear translocation, increases in protein–DNA binding affinity and upregulation of Dspp and Dmp1 gene expression and odontogenesis [172,310,311,312,313]. Additionally, MAPKs induce Msx2 protein phosphorylation and control downstream gene expression [228]. Deletion of p38α MAPK (K14-Cre;p38αfx/fx) caused tooth defects and decreased Dspp gene expression [314]. On the other hand, PI3K/Akt is responsible for BMP signal induction and phosphorylates proteins of Dlx3, Klf4, Osx, and Runx2 and stabilize those proteins, activating tooth-related gene expression, dental mesenchymal cell differentiation, and mineralization [315,316,317,318,319]. Furthermore, PKA and PKC kinases phosphorylated Dlx3, Klf4, Msx1, Msx2, Osx, and Runx2 and trigger their downstream gene expression [320,321,322,323,324,325,326]. BMP–MAPKs, –PI3K/Akt, –PKA, –PKC, and –Smad signaling pathways act synergistically, controlling dentin development. Besides Smad-dependent and Smad-independent pathways, the coordinated activities of TGF-β, transcriptional factors, and other factors are also essential for dentin development and formation [5,226,326,327,328,329,330,331,332,333]. 

## 5. Negative Regulation of BMP Signaling in Odontoblast Differentiation and Dentinogenesis

**Figure 12:**
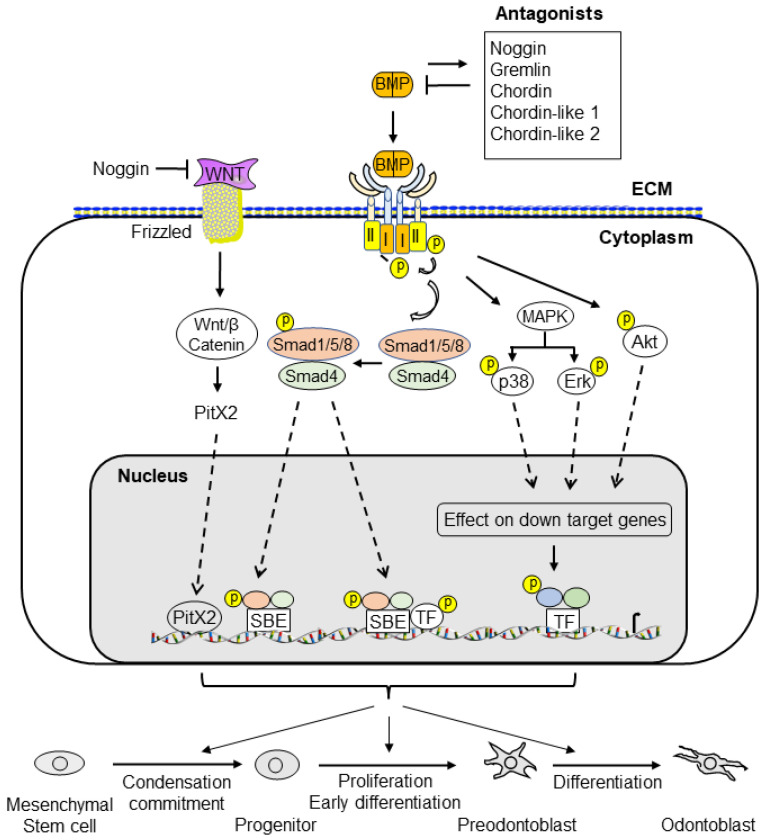
BMP signaling is inhibited by BMP antagonists during tooth development. The antagonists include noggin, gremlin, chordin, chordin-like 1, chordin-like 2, and others. Noggin interacts with BMPs or Wnt ligands and prevents the binding of BMPs to their receptors and Wnt-Frizzled receptors, consequently blocking their signaling and impairing dentin development and formation. Gremlin and chordin, chordin-like 1, and chordin-like 2 also bind to BMPs and interfere with dentinogenesis via canonical Smad and non-Smad signaling pathways. →, activation; ┤, inhibition. Wnt, wingless type MMTV integration site family; SBE, Smad binding element; BMP, bone morphogenetic protein; MAPK, mitogen-activated protein kinase; p38, p38 mitogen-activated protein kinases; ERK, mitogen-activated protein kinase; TF, transcriptional factor; Akt, v-Akt murine thymoma viral oncogene/PKB (protein kinase B). Figure is partially adapted with permission from Katagiri et al., 2016, ref. [22].

BMP signaling is under elaborate regulation to maintain accurate dentin and tissue development and formation. BMP signaling is negatively controlled by several mechanisms, including ligand antagonists, transcriptional repressors, intracellular regulation, and epigenetic factors. Common antagonists targeting BMPs, such as chordin [334], follistatin [335], gremlin [336], and noggin [337,338], have been reported (Figure 12). Noggin, a BMP antagonist, competitively binds BMPs to prevent their binding to receptors, and negatively regulates BMP activities during odontogenesis and osteogenesis. Mutations of noggin in humans are associated with stapes ankylosis, with broad thumbs and toes, hyperopia, and skeletal anomalies, as well as mandibular micrognathia (OMIM 186500) [339,340,341,342]. With noggin deletion, mice died at birth with severe malformed skeletons [343,344]. Skeletal noggin overexpression in mice resulted in osteopenia and retarded bone formation [345,346]. Transgenic mice with noggin gene overexpression showed dentin defects, an increase in dental pulp chamber, a decrease in dentin volume, impaired odontoblastic differentiation, and reduced tooth-related gene expression, including pSmad1/5/8 and Dspp [47,337,338]. Yuan et al. reported that noggin impaired the non-canonical Smad signal of p38 and Erk kinases and Wnt/β-catenin of Pitx2 expression, inhibiting dental cell proliferation and fate as well as tooth development [347]. In addition, Chen et al. found that BMP-2 activated Dspp gene transcription in mouse odontoblastic cells through the NY-F transcriptional factor, but noggin was able to block this activity [5]. Noggin suppressed BMP-2-mediated phosphorylated Smad1/5/8 expression and BMP-2-mediated Akt signaling, inhibiting the expression of ALP, BSP, OCN, OPN, Osx, and Runx2 genes in dental cells [348]. Furthermore, noggin gene expression in osteoblastic cells was induced by BMP-2, -4, and -6 proteins and acted as a negative feedback loop to regulate BMP activity [349]. 

Gremlin is another BMP antagonist for preventing BMP signals. It is required for normal limb patterns and morphology [336]. In gremlin null mice, the expression of Msx1 and Msx2 was high, suggesting that gremlin plays an essential role in BMP signaling during limb development [336]. GREMLIN 2 (GREM2) mutations in humans are associated with dental anomalies, showing isolated tooth agenesis, microdontia, short tooth roots, and taurodontism [350]. Grem2 null mice displayed incisor wear and reduced incisors, as well as dentin layers that were narrow and irregular in appearance and did not completely encircle the dental pulp [351]. Overexpression of the gremlin gene in mice caused developmental defects in teeth. Teeth in the gremlin transgenic mice exhibited tooth fragility and attrition of the tips of the incisors as a result of periodic trimming in an effort to prevent malocclusion and malnutrition, as well as an enlarged pulp chamber with ectopic calcification and thinner dentin and enamel compared with the wild-type controls. In addition, the transgenic mice of gremlin displayed enlarged dental chambers and lacked odontoblast polarization and columnar shape as well as wide pre-dentin and thin dentin, indicating the retarded maturation of odontoblasts and dentin mineralization [352]. Overexpression of GREM1 and GREM2 inhibited bone/tooth-related gene expression of BSP, DMP1, DSPP, OCN, and OPN, as well as transcription factors Osx and Runx2, repressing cell differentiation and biomineralization. By contrast, the knock-down of GREM1 and GREM2 promoted the above gene expression, enhancing ALP activity and mineralization. Furthermore, GREM1 repressed mRNA expression levels of BMP-2, BMP-6, and BMP-7 [353,354]. Guan et al. reported that Grem1 is related to the progress of periodontitis and enhanced activity of NF-κB and interleukin-1β via MAPK signaling and Smad3–β-catenin signaling pathways [355]. GREM2 expression was strong in the bone specimens of osteoporosis patients compared to people without osteoporosis, and human bone marrow stem cells (hBMSCs) mixed with Matrigel containing siRNA GREM2 were transplanted into defect areas of femoral bones in nude mice. siRNA GREM2 robustly promoted new bone formation compared to control groups [353]. 

Chordin (CHD) belongs to a family of proteins that share a cysteine-rich pro-collagen repeat (or chordin-like cysteine-rich repeat (CR)), which is also found in various extracellular matrix proteins [356,357]. Without exception, the homology between CHD family members, including chordin-like 1 (CHDL1) and chordin-like 2 (CHL2), lies within their CRs. The CRs within CHD and CHDLs are responsible for BMP binding, specifically and tightly to BMP-4 [358,359,360,361]. As inhibitors, the interaction of CHD or CHDLs with BMPs prevents BMPs acting on their signaling pathways. Chd, Chdl1, and Chdl2 are expressed in mesenchymal cells [361,362,363]. Overexpression of Chd delayed chondrocyte differentiation and maturation in developing skeletal and cartilage elements [361,363]. Expression of Chd, Chdl1 and Chdl2 genes was detected in mesenchymal cells in the pharynx during early mandibular outgrowth and later in the mandibular process [360,361,362]. Some homozygous mutant Chd mice survived, while others died of cardio-respiratory failure at birth, showing pharyngeal malformations similar to DiGeorge/velocardiofacial (DGS/VCFS) syndromes in humans characterized by cleft palate, facial dysmorphism with low setting of the external ear, small jaw, deafness, and cardiac defects [364,365]. Mice mutant for Chd exhibited mild mandibular defects and craniofacial anomalies characterized by a reduced distance between eye and pinna [362]. CHDL1 mutations in humans are associated with X-linked megalocornea characterized by an increased corneal diameter and deep anterior chamber evident at birth with later onset of mosaic corneal degeneration, arcus juvenilis, and presenile cataracts (MIM 300350) [366,367]. Additionally, Exotic BMP-4 represses Fgf8 and Pax9 transcription and induces Chd expression in mandibular explants in vitro [362]. Chd inhibited BMP-4 expression and osteoblast-like cell (MC3T3-E1) differentiation and mineralization via downregulation of the expression of phosphorylated Smad1/5/8, Alp, Bsp, and Ocn genes [368]. Conversely, Liu et al. reported that knockdown of CHDL1 did not affect hBMSC proliferation but inhibited BMP-4-dependent osteogenic differentiation, showing decreased mRNA expression levels of osteogenic markers, such as ALP, Collα1, OPN, pSmad1/5/8, and Runx2, and reduced mineralization in hBMSCs. Moreover, hBMSCs containing overexpressing pLVX-CHDL1 were mixed with Matrigel and transplanted into defect regions of femoral bones in nude mice. The pLVX-CHDL1 significantly induced new bone formation compared to the controls and Si-CHDL1 mice [369]. These results support the hypothesis that CHD and CHL1 play different biological functions in osteogenesis and that these functions may be dependent on different cell types. 

## 6. Therapeutic Implications of BMPs for Endodontic Regeneration

Tooth decay is the most prevalent non-communicable disease [370]. The World Health Organization (WHO) has defined early childhood caries as a worldwide problem, with a prevalence between 60% and 90%. In addition, more than 90% of all adults have experienced this disease [371,372,373,374,375]. When left untreated, dental caries can lead to disease of the dental pulp with sequent pulpal infection, necrosis, and loss of tooth vitality and possibly function, as well as eventual tooth loss. However, approximately 50% of cases require revision within 5–10 years after restorative treatment [376]. Additionally, any traditional artificial restorative materials might fail due to inappropriate physical, biocompatible, and mechanical properties [372,377,378]. The material pulls away from the cavity wall and microleakage can occur between the cavity and dental material. Microleakage may cause bacterial invasion, resulting in recurrent caries [377,378]. Therefore, despite several advances in dental restorative materials, it is required for novel therapeutic restorative approaches in dentistry to maintain a healthy dentition. Regenerative endodontic treatment (RET) is defined as ‘biologically based procedures designed to replace damaged tooth structures, including dentin and root structures as well as the pulp–dentine complex’ damaged by infection, trauma, or developmental anomalies with respect to immature permanent teeth with necrotic pulp [379]. In general, RET involves three principles of bioengineering: stem cells (SCs), biomaterials, and growth factors [380]. Dental pulp stem cells (DPSCs) and stem cells from human exfoliated deciduous teeth (SHEDs) and from the apical papilla (SCAPs) were isolated and these SCs were able to proliferate and differentiate into odontoblast-like cells, forming dentin structures in the given niches [381,382,383]. Recent preclinical and clinical studies have demonstrated that exogenous DPSCs were transplanted into injured teeth in patients, and these SCs regenerated dentin formation and provided evidence of neuro-vascularization [384,385]. SC proliferation and differentiation are regulated by intrinsic and extrinsic signaling pathways. Growth factors and advanced biomaterials provide a physicochemical and biological three-dimensional niche for cell attachment, proliferation, differentiation, and neo-tissue genesis. 

BMP-2 has been shown to be odontogenic and osteogenic both in vitro and in vivo [32,386,387,388,389,390,391,392]. BMP-2 has many functions: it stimulates the differentiation of DPSCs, SHED, and human tooth germ SCs into odontoblast-like cells, forming dentin [32,386,389,393]. Recombinant human BMP-2 (rhBMP-2) was approved for use for accelerating bone fusion in slow-healing fractures [394]. In addition, rhBMP-2 was used to treat periodontal wound healing and new bone formation in the maxilla and mandibles of animals and humans [395,396,397,398,399,400,401,402,403]. 

Previously, Nakashima used rhBMP-2 and rhBMP-4 capped with an inactive dentin matrix, respectively, and the mixed compounds were transplanted into amputated pulps in dogs. At two months, tubular dentin was generated in the lower part and osteo-dentin in the upper part of the amputated pulps in the rhBMP-2 or -4, with the inactive dentin matrix groups compared to the inactive dentin matrix groups alone, indicating that rhBMP-2 and rhBMP-4 are able to induce the differentiation of adult dental pulp cells into odontoblastic cells, forming dentin structures [386,404,405]. Human SCAPs with nanofibrous microspheres (NF-MSs) as carriers in combination with controlled release of rhBMP-2 were implanted into subcutaneous regions in nude mice. rhBMP-2 induced differentiation of SCAPs into odontoblast-like cells and DSPP gene expression and osteodentin formation [393]. BMP-2 promotes dental mesenchymal cell differentiation and dentin formation through DSPP signaling [5,157]. DSP and domains of DSP peptides are able to induce dental mesenchymal cells into odontoblasts and mineralization as well as vascularization [406,407,408,409]. However, recently, side effects of BMP-2 usage have been reported with increasing frequency, causing safety concerns, including concerns about ectopic bone formation, osteoclast-mediated bone resorption, postoperative inflammation, and even tumorigenesis [410]. Thus, BMP-2 and other BMPs in the RET for precision medicine need to be further investigated in the future.

## 7. Conclusions

In this review, we have summarized recent achievements in research into BMP signaling pathways during odontogenesis. Despite new insights gained during the past decades, many questions still need to be answered regarding genetic, cellular, and molecular understanding of the biological functions of BMPs. Downstream signaling pathways of BMPs and BMP receptors are much more complex in tooth development and formation, especially odontogenesis. Little is known about the target genes of divergent BMP signaling. Genetic analyses in humans and knockout mouse models obviously demonstrate that BMPs and their target genes play critical roles in communication between ameloblasts and odontoblasts and spatial–temporal dental mesenchymal cell proliferation and differentiation, as well as biomineralization during dentin development and formation, besides other signaling pathways. Further analyses of these cell and tissue morphologies should be conducted and the molecular signals should be explored further for a better understanding of the mechanisms underlying each step in the cellular processes that occur during dentinogenesis.

BMP signaling is essential to and has diverse roles in odontogenesis. Intensive studies in human genetics reveal that mutation of BMPs, BMP receptors, antagonists, and their downstream genes, such as transcription factors, Runx2, Osx, Dlx3, MSX1, MSX2, and PAX9, as well as collagen and non-collagen proteins, including DSPP and DMP1, lead to dentin anomalies. Conditional gene knockout of BMPs and their signaling molecules in mice results in their developing similar phenotypes to those observed in human beings. In vitro and in vivo studies further dissect the atlas of the BMP signaling pathways of physiology and pathogenesis during dentin development and formation. Knowledge obtained from the analyses of BMP signaling in dentinogenesis should also shed light on understanding the molecular mechanisms of these human diseases and provide potential clues for treating dentin defects.

## Figures and Tables

**Table 1:** Expression of BMPs during tooth development.

Genes	Initiate		Bud		Cap		Bell		Dif. & Sec.		Root	
	DE	DM	DE	DM	DE	DM	DE	DM	DE	DM	DE	DM
BMP−2	+/−	−	+	+	+	+/−	+	+	+	+	+/−	+
BMP−3	n/a	n/a	+/−	n/a	+/−	+	n/a	+	n/a	+	n/a	+
BMP−4	+/−	+	+/−	+	+	+	+/−	+	+/−	+	+/−	+
BMP−5	−	−	−	−	−	−	+/−	−	+	+/−	+	−
BMP−6	−	−	+	+	+	+	−	+	−	+	+	+
BMP−7	+	+/−	+	−	+	−	+	+	+	+	+	+
Alk−1	n/a	n/a	+	+	n/a	n/a	n/a	n/a	n/a	n/a	n/a	n/a
Alk−2	n/a	−	+	+	n/a	n/a	n/a	n/a	+	+	+	+
Alk−3	n/a	n/a	+	n/a	+	+	+	+	+	+	+	+
Alk−5	n/a	n/a	+	+	+	+	+	+	+	+	n/a	n/a
Alk−6	n/a	n/a	+	+	+	−	+	+	+	+	+	+
Bmpr−II	n/a	n/a	+	+	+	+	+	+	+	+	+	+

+, yes; −, no; n/a, unknown; DE, dental epithelium; DM, dental mesenchyme; Dif., differentiation; Sec., secretory. The gene expression patterns of Bmp2 from Table 1 is partially from Website: https://bite-it.helsinki.fi/ (accessed on 17 May 2022).

## References

[1] Mitsiadis T.A., Orsini G., Jimenez-Rojo L. (2015). Stem cell-based approaches in dentistry. Eur. Cell Mater..

[2] Nanci A. (2012). Ten Cate’s Oral Histology: Development, Structure, and Function.

[3] Zhang Y.D., Chen Z., Song Y.Q., Liu C., Chen Y.P. (2005). Making a tooth: Growth factors, transcription factors, and stem cells. Cell Res..

[4] Thesleff I. (2003). Epithelial-mesenchymal signaling regulating tooth morphogenesis. J. Cell Sci..

[5] Chen S., Gluhak-Heinrich J., Martinez M., Li T., Wu Y., Chuang H.H., Chen L., Dong J., Gay I., MacDougall M. (2008). Bone morphogenetic protein 2 mediates dentin sialophosphoprotein expression and odontoblast differentiation via NF-Y signaling. J. Biol. Chem..

[6] Liu M., Li W., Xia X., Wang F., MacDougall M., Chen S. (2021). Dentine sialophosphoprotein signal in dentineogenesis and dentine regeneration. Eur. Cells Mater..

[7] Linde A., Goldberg M. (1993). Dentinogenesis (review). Crit. Rev. Oral. Biol. Med..

[8] MacDougall M., Simmons D., Luan X., Nydegger J., Feng J., Gu T.T. (1997). Dentin phosphoprotein and dentin sialoprotein are cleavage products expressed from a single transcript coded by a gene on human chromosome 4. Dentin phosphoprotein DNA sequence determination. J. Biol. Chem..

[9] Lopez-Cazaux S., Bluteau G., Magne D., Lieubeau B., Guicheux J., Alliot-Licht B. (2006). Culture medium modulates the behavior of human dental pulpderived cells: Technical note. Eur. Cell Mater..

[10] Orban B.J. (1980). Orban’s Oral Histology and Embryology.

[11] Li J., Parada C., Chai Y. (2017). Cellular and molecular mechanisms of tooth root development. Development.

[12] Tucker A.S., Sharpe P. (2004). The cutting-edge of mammalian development; how the embryo makes teeth. Nat. Rev. Genet..

[13] Chai Y., Maxson R.E. (2006). Recent advances in craniofacial morphogenesis. Dev. Dyn..

[14] Urist M.R. (1965). Bone: Formation by autoinduction. Science.

[15] Wang E.A., Rosen V., Cordes P., Hewick R.M., Kriz M.J., Luxenberg D.P., Sibley B.S., Wozney J.M. (1988). Purification and characterization of other distinct bone-inducing factors. Proc. Natl. Acad. Sci. USA.

[16] Wozney J.M., Rosen V., Celeste A.J., Mitsock L.M., Whitters M.J., Kriz R.W., Hewick R.M., Wang E.A. (1988). Novel regulators of bone formation: Molecular clones and activities. Science.

[17] Celeste A.J., Iannazzi J.A., Taylor R.C., Hewick R.M., Rosen V., Wang E.A., Wozney J.M. (1990). Identification of transforming growth factor beta family members present in bone-inductive protein purified from bovine bone. Proc. Natl. Acad. Sci. USA.

[18] Ozkaynak E., Rueger D.C., Drier E.A., Corbett C., Ridge R.J., Sampath T.K., Oppermann H. (1990). OP-1 cDNA encodes an osteogenic protein in the TGF-beta family. EMBO J..

[19] Sampath T.K., Coughlin J.E., Whetstone R.M., Banach D., Corbett C., Ridge R.J., Ozkaynak E., Oppermann H., Rueger D.C. (1990). Bovine osteogenic protein is composed of dimers of OP-1 and BMP-2A, two members of the transforming growth factor-beta superfamily. J. Biol. Chem..

[20] Sampath T.K., Reddi A.H. (1983). Homology of bone-inductive proteins from human, monkey, bovine, and rat extracellular matrix. Proc. Natl. Acad. Sci. USA.

[21] Carreira A., Lojudice F., Halcsik E., Navarro R., Sogayar M., Granjeiro J. (2014). Bone morphogenetic proteins: Facts, challenges, and future perspectives. J. Dent. Res..

[22] Katagiri T., Watabe T. (2016). Bone morphogenetic proteins. Cold Spring Harb. Perspect. Biol..

[23] Salazar V.S., Gamer L.W., Rosen V. (2016). BMP signalling in skeletal development, disease and repair. Nat. Rev. Endocrinol..

[24] Madaleno C.D.S., Jatzlau J., Knaus P. (2020). BMP signalling in a mechanical context—Implications for bone biology. Bone.

[25] Berg T., Wozney J., Thesleff I. (1997). Expression patterns of bone morphogenetic proteins (bmps) in the developing mouse tooth suggest poles in morphogenesis and cell differentiation. Dev. Dyn..

[26] Nadiri A., Kuchler-Bopp S., Haikel Y., Lesot H. (2004). Immunolocalization of BMP-2/-4, FGF-4, and WNT10b in the developing mouse first lower molar. J. Histochem. Cytochem..

[27] Heikinheimo K., Bègue-Kirn C., Ritvos O., Tuuri T., Ruch J.V. (1998). Activin and bone morphogenetic protein (BMP) signalling during tooth development. Eur. J. Oral Sci..

[28] Dong X., Shen B., Ruan N., Guan Z., Zhang Y., Chen Y., Hu X. (2014). Expression patterns of genes critical for BMP signaling pathway in developing human primary tooth germs. Histochem. Cell Biol..

[29] Gao Z., Wang L., Wang F., Zhang C., Wang J., He J., Wang S. (2018). Expression of BMP2/4/7 during the odontogenesis of deciduous molars in miniature pig embryos. Histochem. J..

[30] Yamashiro T., Tummers M., Thesleff I. (2003). Expression of bone morphogenetic proteins and MSX genes during root formation. J. Dent. Res..

[31] Kémoun P., Laurencin-Dalicieux S., Rue J., Vaysse F., Roméas A., Arzate H., Conte-Auriol F., Farges J., Salles J., Brunel G. (2007). Localization of STRO-1, BMP-2/-3/-7, BMP receptors and phosphorylated Smad-1 during the formation of mouse periodontium. Tissue Cell.

[32] Casagrande L., Demarco F., Zhang Z., Araujo F., Shi S., Nör J. (2010). Dentin-derived BMP-2 and odontoblast differentiation. J. Dent. Res..

[33] Lu Y., Qian Y., Zhang J., Gong M., Wang Y., Gu N., Ma L., Xu M., Ma J., Zhang W. (2016). Genetic variants of BMP2 and their association with the risk of non-syndromic tooth agenesis. PLoS ONE.

[34] Wang H., Liu Y., Liu H.C., Han D., Feng H.L. (2019). Detection and functional analysis of BMP2 gene mutation in patients with tooth agenesis. Beijing Da Xue Xue Bao Yi Xue Ban.

[35] Zhang H., Bradley A. (1996). Mice deficient for BMP2 are nonviable and have defects in amnion/chorion and cardiac development. Development.

[36] Yang W., Harris M., Cui Y., Mishina Y., Harris S., Gluhak-Heinrich J. (2011). Bmp2 is required for odontoblast differentiation and pulp vasculogenesis. J. Dent. Res..

[37] Yang G., Yuan G., MacDougall M., Zhi C., Chen S. (2017). BMP-2 induced Dspp transcription is mediated by Dlx3/Osx signaling pathway in odontoblasts. Sci. Rep..

[38] Malik Z., Alexiou M., Hallgrimsson B., Economides A., Luder H., Graf D. (2018). Bone morphogenetic protein 2 coordinates early tooth mineralization. J. Dent. Res..

[39] Wu L., Wang F., Donly K.J., Wan C., Luo D., Harris S.E., MacDougall M., Chen S. (2015). Establishment of immortalized mouse BMP2 knock-out dental papilla mesenchymal cells necessary for study of odontoblastic differentiation and odontogenesis. J. Cell. Physiol..

[40] Takahashi H., Ikeda T. (1996). Transcripts for two members of the transforming growth factor-beta superfamily BMP-3 and BMP-7 are expressed in developing rat embryos. Dev. Dyn..

[41] Thomadakis G., Ramoshebi L.N., Crooks J., Rueger D.C., Ripamonti U. (1999). Immunolocalization of bone morphogenetic protein-2 and -3 and osteogenic protein-1 during murine tooth root morphogenesis and in other craniofacial structures. Eur. J. Oral Sci..

[42] Gao Y., Zhang M., Tian X., Wang M., Zhang F. (2018). Experimental animal study on BMP-3 expression in periodontal tissues in the process of orthodontic tooth movement. Exp. Ther. Med..

[43] Huang Y., Lu Y., Mues G., Wang S., Bonds J., D’Souza R. (2013). Functional evaluation of a novel tooth agenesis-associated bone morphogenetic protein 4 prodomain mutation. Eur. J. Oral Sci..

[44] Yu M., Wang H., Fan Z., Xie C., Liu H., Liu Y., Han D., Wong S.-W., Feng H. (2019). BMP4 mutations in tooth agenesis and low bone mass. Arch. Oral Biol..

[45] Winnier G., Blessing M., Labosky P.A., Hogan B.L. (1995). Bone morphogenetic protein-4 is required for mesoderm formation and patterning in the mouse. Genes Dev..

[46] Gluhak-Heinrich J., Guo D., Yang W., Harris M., Lichtler A., Kream B., Zhang J., Feng J., Smith L., Dechow P. (2010). New roles and mechanism of action of BMP4 in postnatal tooth cytodifferentiation. Bone.

[47] Jani P., Liu C., Zhang H., Younes K., Benson M.D., Qin C. (2018). The role of bone morphogenetic proteins 2 and 4 in mouse dentinogenesis. Arch. Oral Biol..

[48] Pathi S., Rutenberg J.B., Johnson R.L., Vortkamp A. (1999). Interaction of Ihh and BMP/Noggin signaling during cartilage differentiation. Dev. Biol..

[49] Kettunen P., Nie X., Kvinnsland I.H., Luukko K. (2006). Histological development and dynamic expression of Bmp2–6 mRNAs in the embryonic and postnatal mousecranial base. Anat. Rec. Part A Discov. Mol. Cell. Evol. Biol..

[50] Mailhot G., Yang M., Mason-Savas A., MacKay C.A., Leav I., Odgren P.R. (2007). BMP-5 expression increases during chondrocyte differentiation in vivo and in vitro and promotes proliferation and cartilage matrix synthesis in primary chondrocyte cultures. J. Cell. Physiol..

[51] Kingsley D.M., Bland A.E., Grubber J.M., Marker P.C., Russell L.B., Copeland N.G., Jenkins N.A. (1992). The mouse short ear skeletal morphogenesis locus is associated with defects in a bone morphogenetic member of the TGF beta superfamily. Cell.

[52] Liu W., Wang Q., Guo Y., Lin L., Yang Q., Jiang H. (2021). Whole-genome sequencing identifies two novel rare mutations in BMP5 and BMP2 in monozygotic twins with microtia. J. Craniofacial Surg..

[53] Wilkins J.M., Southam L., Mustafa Z., Chapman K., Loughlin J. (2009). Association of a functional microsatellite within intron 1 of the BMP5 gene with susceptibility to osteoarthritis. BMC Med. Genet..

[54] Liang W., Gao B., Xu G., Weng D., Xie M., Qian Y. (2014). Association between single nucleotide polymorphisms of asporin (ASPN) and BMP5 with the risk of knee osteoarthritis in a Chinese Han population. Cell Biophys..

[55] Shih H.-Y., Hsu S.-Y., Ouyang P., Lin S.-J., Chou T.-Y., Chiang M.-C., Cheng Y.-C. (2016). Bmp5 regulates neural crest cell survival and proliferation via two different signaling pathways. Stem Cells.

[56] Heikinheimo K. (1994). Stage-specific expression of decapentaplegic-vg-related genes 2, 4, and 6 (bone morphogenetic proteins 2, 4, and 6) during human tooth morphogenesis. J. Dent. Res..

[57] Oralová V., Chlastáková I., Radlanski R.J., Matalová E. (2014). Distribution of BMP6 in the alveolar bone during mouse mandibular molar eruption. Connect. Tissue Res..

[58] Meynard D., Kautz L., Darnaud V., Canonne-Hergaux F., Coppin H., Roth M.-P. (2009). Lack of the bone morphogenetic protein BMP6 induces massive iron overload. Nat. Genet..

[59] Daher R., Kannengiesser C., Houamel D., Lefebvre T., Bardou-Jacquet E., Ducrot N., de Kerguenec C., Jouanolle A.-M., Robreau A.-M., Oudin C. (2016). Heterozygous mutations in BMP6 pro-peptide lead to inappropriate hepcidin synthesis and moderate iron overload in humans. Gastroenterology.

[60] Piubelli C., Castagna A., Marchi G., Rizzi M., Busti F., Badar S., Marchetti M., De Gobbi M., Roetto A., Xumerle L. (2017). Identification of new BMP6 pro-peptide mutations in patients with iron overload. Am. J. Hematol..

[61] Solloway M.J., Dudley A.T., Bikoff E.K., Lyons K.M., Hogan B.L., Robertson E.J. (1998). Mice lacking Bmp6 function. Dev. Genet..

[62] Perry M.J., McDougall K.E., Hou S.-C., Tobias J. (2008). Impaired growth plate function in bmp-6 null mice. Bone.

[63] Cleves P.A., Hart J.C., Agoglia R.M., Jimenez M.T., Erickson P.A., Gai L., Miller C.T. (2018). An intronic enhancer of Bmp6 underlies evolved tooth gain in sticklebacks. PLoS Genet..

[64] Wang Y.-H., Rutherford B., Upholt W.B., Mina M. (1999). Effects of BMP-7 on mouse tooth mesenchyme and chick mandibular mesenchyme. Dev. Dyn..

[65] Helder M., Karg H., Bervoets T., Vukicevic S., Burger E., D’Souza R., Wöltgens J., Karsenty G., Bronckers A. (1998). Bone morphogenetic protein-7 (osteogenic protein-1, OP-1) and tooth development. J. Dent. Res..

[66] Godin R.E., Takaesu N.T., Robertson E.J., Dudley A.T. (1998). Regulation of BMP7 expression during kidney development. Development.

[67] Morgan E.A., Nguyen S.B., Scott V., Stadler H.S. (2003). Loss of Bmp7 and Fgf8 signaling in Hoxa13-mutant mice causes hypospadia. Development.

[68] Chen N., Zhao S., Jolly A., Wang L., Pan H., Yuan J., Chen S., Koch A., Ma C., Tian W. (2021). Perturbations of genes essential for Müllerian duct and Wölffian duct development in Mayer-Rokitansky-Küster-Hauser syndrome. Am. J. Hum. Genet..

[69] Jeremias F., Pierri R.A., Souza J.F., Fragelli C.M.B., Restrepo M., Finoti L.S., Bussaneli D.G., Cordeiro R.C., Secolin R., Maurer-Morelli C.V. (2016). Family-based genetic association for molar-incisor hypomineralization. Caries Res..

[70] Dudley A.T., Lyons K.M., Robertson E.J. (1995). A requirement for bone morphogenetic protein-7 during development of the mammalian kidney and eye. Genes Dev..

[71] Luo G., Hofmann C., Bronckers A.L., Sohocki M., Bradley A., Karsenty G. (1995). BMP-7 is an inducer of nephrogenesis, and is also required for eye development and skeletal patterning. Genes Dev..

[72] Saito K., Takahashi K., Asahara M., Kiso H., Togo Y., Tsukamoto H., Huang B., Sugai M., Shimizu A., Motokawa M. (2016). Effects of Usag-1 and Bmp7 deficiencies on murine tooth morphogenesis. BMC Dev. Biol..

[73] Zurowski C., Jamniczky H., Graf D., Theodor J. (2018). Deletion/loss of bone morphogenetic protein 7 changes tooth morphology and function in Mus musculus: Implications for dental evolution in mammals. R. Soc. Open Sci..

[74] Malik Z., Roth D.M., Eaton F., Theodor J.M., Graf D. (2020). Mesenchymal Bmp7 controls onset of tooth mineralization: A novel way to regulate molar cusp shape. Front. Physiol..

[75] Verschueren K., Dewulf N., Goumans M.J., Lonnoy O., Feijen A., Grimsby S., Vande Spiegle K., ten Dijke P., Morén A., Vanscheeuwijck P. (1995). Expression of type I and type IB receptors for activin in midgestation mouse embryos suggests distinct functions in organogenesis. Mech. Dev..

[76] Zhang X., Liu Q., Zhao H., Hu Y., Liu C., Yan G., Li D., Mishina Y., Shi C., Sun H. (2018). ACVR1 is essential for periodontium development and promotes alveolar bone formation. Arch. Oral Biol..

[77] Gu K., Smoke R.H., Rutherford R. (1996). Expression of genes for bone morphogenetic proteins and receptors in human dental pulp. Arch. Oral Biol..

[78] Toyono T., Nakashima M., Kuhara S., Akamine A. (1997). Temporal changes in expression of transforming growth factor-β superfamily members and their receptors during bovine preodontoblast differentiation in vitro. Arch. Oral Biol..

[79] Toyono T., Nakashima M., Kuhara S., Akamine A. (1997). Expression of TGF-beta superfamily receptors in dental pulp. J. Dent. Res..

[80] Cheifetz S. (1999). BMP receptors in limb and tooth formation. Crit. Rev. Oral Biol. Med..

[81] Chien H.-H., Lin W.-L., Cho M.-I. (1999). Expression of TGF-beta isoforms and their receptors during mineralized nodule formation by rat periodontal ligament cells in vitro. J. Periodontal Res..

[82] McDonald J.E., Miller F.J., Hallam S.E., Nelson L., Marchuk D.A., Ward K.J. (2000). Clinical manifestations in a large hereditary hemorrhagic telangiectasia (HHT) type 2 kindred. Am. J. Med. Genet..

[83] Wehner L.-E., Folz B.J., Argyriou L., Twelkemeyer S., Teske U., Geisthoff U.W., Werner J.A., Engel W., Nayernia K. (2006). Mutation analysis in hereditary haemorrhagic telangiectasia in Germany reveals 11 novel ENG and 12 novel ACVRL1/ALK1 mutations. Clin. Genet..

[84] Park H., Furtado J., Poulet M., Chung M., Yun S., Lee S., Sessa W.C., Franco C.A., Schwartz M.A., Eichmann A. (2021). Defective flow-migration coupling causes arteriovenous malformations in hereditary hemorrhagic telangiectasia. Circulation.

[85] Zhang X., Shi C., Zhao H., Zhou Y., Hu Y., Yan G., Liu C., Li D., Hao X., Mishina Y. (2018). Distinctive role of ACVR1 in dentin formation: Requirement for dentin thickness in molars and prevention of osteodentin formation in incisors of mice. Histochem. J..

[86] Fiori J.L., Billings P.C., De La Peña L.S., Kaplan F.S., Shore E.M. (2006). Dysregulation of the BMP-p38 MAPK signaling pathway in cells from patients with Fibrodysplasia Ossificans Progressiva (FOP). J. Bone Miner. Res..

[87] Kaplan F.S., Kobori J.A., Orellana C., Calvo I., Rosello M., Martinez F., Lopez B., Xu M., Pignolo R.J., Shore E.M. (2015). Multi-system involvement in a severe variant of fibrodysplasia ossificans progressiva (ACVR1 c.772G > A.; R258G): A report of two patients. Am. J. Med. Genet. A.

[88] Schoenmaker T., Mokry M., Micha D., Netelenbos C., Bravenboer N., Gilijamse M., Eekhoff E., de Vries T. (2021). Activin-A induces early differential gene expression exclusively in periodontal ligament fibroblasts from *Fibrodysplasia Ossificans Progressiva* Patients. Biomedicines.

[89] Mishina Y., Suzuki A., Ueno N., Behringer R.R. (1995). Bmpr encodes a type I bone morphogenetic protein receptor that is essential for gastrulation during mouse embryogenesis. Genes Dev..

[90] Andl T., Ahn K., Kairo A., Chu E.Y., Wine-Lee L., Reddy S.T., Croft N.J., Cebra-Thomas J.A., Metzger D., Chambon P. (2004). Epithelial *Bmpr1a* regulates differentiation and proliferation in postnatal hair follicles and is essential for tooth development. Development.

[91] Li L., Lin M., Wang Y., Cserjesi P., Chen Z., Chen Y. (2011). BmprIa is required in mesenchymal tissue and has limited redundant function with BmprIb in tooth and palate development. Dev. Biol..

[92] Omi M., Kulkarni A.K., Raichur A., Fox M., Uptergrove A., Zhang H., Mishina Y. (2019). BMP-Smad signaling regulates postnatal crown Dentinogenesis in mouse molar. JBMR Plus.

[93] Li L., Wang Y., Lin M., Yuan G., Yang G., Zheng Y., Chen Y. (2013). Augmented BMPRIA-mediated BMP signaling in cranial neural crest lineage leads to cleft palate formation and delayed tooth differentiation. PLoS ONE.

[94] Iseki S., Osumi-Yamashita N., Miyazono K., Franzén P., Ichijo H., Ohtani H., Hayashi Y., Eto K. (1995). Localization of transforming growth factor-β type I and type II receptors in mouse development. Exp. Cell Res..

[95] Zhang H., Zhan Y., Zhang Y., Yuan G., Yang G. (2020). Dual roles of TGF-β signaling in the regulation of dental epithelial cell proliferation. Histochem. J..

[96] Guo W., Fan Z., Wang S., Du J. (2019). ALK5 is essential for tooth germ differentiation during tooth development. Biotech. Histochem..

[97] Gao Y., Li D., Han T., Sun Y., Zhang J. (2009). TGF-beta1 and TGFBR1 are expressed in ameloblasts and promote MMP20 expression. Anat. Rec..

[98] Sloan A., Matthews J., Smith A. (1999). TGF-β receptor expression in human odontoblasts and pulpal cells. Histochem. J..

[99] Sloan A., Couble M.-L., Bleicher F., Magloire H., Smith A., Farges J.-C. (2001). Expression of TGF-β receptors I and II in the human dental pulp by in situ hybridization. Adv. Dent. Res..

[100] Hosoya A., Kim J.-Y., Cho S.-W., Jung H.-S. (2008). BMP4 signaling regulates formation of Hertwig’s epithelial root sheath during tooth root development. Cell Tissue Res..

[101] Yi S., Daluiski A., Pederson R., Rosen V., Lyons K. (2000). The type I BMP receptor BMPRIB is required for chondrogenesis in the mouse limb. Development.

[102] Shi C., Iura A., Terajima M., Liu F., Lyons K., Pan H., Zhang H., Yamauchi M., Mishina Y., Sun H. (2016). Deletion of BMP receptor type IB decreased bone mass in association with compromised osteoblastic differentiation of bone marrow mesenchymal progenitors. Sci. Rep..

[103] Lee H.-K., Park J.-T., Cho Y.-S., Bae H.-S., Cho M.-I., Park J.-C. (2011). Odontogenic ameloblasts-associated protein (ODAM), via phosphorylation by bone morphogenetic protein receptor type IB (BMPR-IB), is implicated in ameloblast differentiation. J. Cell. Biochem..

[104] Hinck A.P., Mueller T.D., Springer T.A. (2016). Structural biology and evolution of the TGF-β family. Cold Spring Harb. Perspect. Biol..

[105] Yadin D., Knaus P., Mueller T.D. (2016). Structural insights into BMP receptors: Specificity, activation and inhibition. Cytokine Growth Factor Rev..

[106] Sengle G., Charbonneau N.L., Ono R.N., Sasaki T., Alvarez J., Keene D.R., Bächinger H.P., Sakai L.Y. (2008). Targeting of bone morphogenetic protein growth factor complexes to fibrillin. J. Biol. Chem..

[107] Mueller T.D., Nickel J. (2012). Promiscuity and specificity in BMP receptor activation. FEBS Lett..

[108] Shi M., Zhu J., Wang R., Chen X., Mi L., Walz T., Springer T.A. (2011). Latent TGF-β structure and activation. Nature.

[109] Nelsen S.M., Christian J.L. (2009). Site-specific cleavage of BMP4 by Furin, PC6, and PC7. J. Biol. Chem..

[110] Bragdon B., Moseychuk O., Saldanha S., King D., Julian J., Nohe A. (2011). Bone morphogenetic proteins: A critical review. Cell. Signal..

[111] Saremba S., Nickel J., Seher A., Kotzsch A., Sebald W., Mueller T.D. (2007). Type I receptor binding of bone morphogenetic protein 6 is dependent on N-glycosylation of the ligand. FEBS J..

[112] Garrigue-Antar L., Hartigan N., Kadler K.E. (2002). Post-translational modification of bone morphogenetic protein-1 is required for secretion and stability of the protein. J. Biol. Chem..

[113] Hashimoto O., Moore R.K., Shimasaki S. (2005). Posttranslational processing of mouse and human BMP-15: Potential implication in the determination of ovulation quota. Proc. Natl. Acad. Sci. USA.

[114] Little S.C., Mullins M.C. (2009). Bone morphogenetic protein heterodimers assemble heteromeric type I receptor complexes to pattern the dorsoventral axis. Nat. Cell Biol..

[115] Sieber C., Kopf J., Hiepen C., Knaus P. (2009). Recent advances in BMP receptor signaling. Cytokine Growth Factor Rev..

[116] Brazil D.P., Church R.H., Surae S., Godson C., Martin F. (2015). BMP signalling: Agony and antagony in the family. Trends Cell Biol..

[117] Ulloa L., Doody J., Massagué J. (1999). Inhibition of transforming growth factor-β/SMAD signalling by the interferon-gamma/STAT pathway. Nature.

[118] Liu Y., Ren W., Warburton R., Toksoz D., Fanburg B.L. (2009). Serotonin induces Rho/ROCKdependent activation of Smads 1/5/8 in pulmonary artery smooth muscle cells. FASEB J..

[119] Moreno-Miralles I., Schisler J.C., Patterson C. (2009). New insights into bone morphogenetic protein signaling: Focus on angiogenesis. Curr. Opin. Hematol..

[120] Israel D.I., Nove J., Kerns K.M., Kaufman R.J., Rosen V., Cox K.A., Wozney J.M. (1996). Heterodimeric bone morphogenetic proteins show enhanced activity in vitro and in vivo. Growth Factors.

[121] Guo J., Wu G. (2012). The signaling and functions of heterodimeric bone morphogenetic proteins. Cytokine Growth Factor Rev..

[122] Suzuki A., Kaneko E., Maeda J., Ueno N. (1997). Mesoderm induction by BMP-4 and -7 heterodimers. Biochem. Biophys. Res. Commun..

[123] Yuan S., Pan Q., Liu W., Wu B., Han X., Bi Z. (2011). Recombinant BMP 4/7 fusion protein induces differentiation of bone marrow stem cells. J. Cell Biochem..

[124] Griffith D.L., Keck P.C., Sampath T.K., Rueger D.C., Carlson W.D. (1996). Three-dimensional structure of recombinant human osteogenic protein 1: Structural paradigm for the transforming growth factor b superfamily. Proc. Natl. Acad. Sci. USA.

[125] Scheufler C., Sebald W., Hülsmeyer M. (1999). Crystal structure of human bone morphogenetic protein-2 at 2.7 Å resolution. J. Mol. Biol..

[126] Brown M.A., Zhao Q., Baker K.A., Naik C., Chen C., Pukac L., Singh M., Tsareva T., Parice Y., Mahoney A. (2005). Crystal structure of BMP-9 and functional interactions with pro-region and receptors. J. Biol. Chem..

[127] Schreuder H., Liesum A., Pohl J., Kruse M., Koyama M. (2005). Crystal structure of recombinant human growth and differentiation factor 5: Evidence for interaction of the type I and type II receptor-binding sites. Biochem. Biophys. Res. Commun..

[128] Allendorph G.P., Isaacs M.J., Kawakami Y., Belmonte J.C.I., Choe S. (2007). BMP-3 and BMP-6 structures illuminate the nature of binding specificity with receptors. Biochemistry.

[129] Kirsch T., Sebald W., Dreyer M.K. (2000). Crystal structure of the BMP-2–BRIA ectodomain complex. Nat. Genet..

[130] Groppe J., Greenwald J., Wiater E., Rodriguez-Leon J.M., Economides A.N., Kwiatkowski W., Affolter M., Vale W.W., Belmonte J.C.I., Choe S. (2002). Structural basis of BMP signalling inhibition by the cystine knot protein Noggin. Nature.

[131] Greenwald J., Groppe J., Gray P., Wiater E., Kwiatkowski W., Vale W., Choe S. (2003). The BMP7/ActRII extracellular domain complex provides new insights into the cooperative nature of receptor assembly. Mol. Cell..

[132] Keller S.L., Nickel J., Zhang J.-L., Sebald W., Mueller T.D. (2004). Molecular recognition of BMP-2 and BMP receptor IA. Nat. Struct. Mol. Biol..

[133] Allendorph G.P., Vale W.W., Choe S. (2006). Structure of the ternary signaling complex of a TGF-beta superfamily member. Proc. Natl. Acad. Sci. USA.

[134] Healey E.G., Bishop B., Elegheert J., Bell C.H., Padilla-Parra S., Siebold C. (2015). Repulsive guidance molecule is a structural bridge between neogenin and bone morphogenetic protein. Nat. Struct. Mol. Biol..

[135] Ducy P., Zhang R., Geoffroy V., Ridall A.L., Karsenty G. (1997). Osf2/Cbfa1: A transcriptional activator of osteoblast differentiation. Cell.

[136] Komori T., Yagi H., Nomura S., Yamaguchi A., Sasaki K., Deguchi K., Shimizu Y., Bronson R.T., Gao Y.H., Inada M. (1997). Targeted disruption of Cbfa1 results in a complete lack of bone formation owing to maturational arrest of osteoblasts. Cell.

[137] Lee B., Thirunavukkarasu K., Zhou L., Pastore L., Baldini A., Hecht J., Geoffroy V., Ducy P., Karsenty G. (1997). Missense mutations abolishing DNA binding of the osteoblast-specific transcription factor OSF2/CBFA1 in cleidocranial dysplasia. Nat. Genet..

[138] Mundlos S., Otto F., Mundlos C., Mulliken J., Aylsworth A., Albright S., Lindhout D., Cole W., Henn W., Knoll J. (1997). Mutations involving the transcription factor CBFA1 cause cleidocranial dysplasia. Cell.

[139] Otto F., Thornell A.P., Crompton T., Denzel A., Gilmour K.C., Rosewell I.R., Stamp G.W., Beddington R.S., Mundlos S., Olsen B.R. (1997). Cbfa1, a candidate gene for cleidocranial dysplasia syndrome, is essential for osteoblast differentiation and bone development. Cell.

[140] Quack I., Vonderstrass B., Stock M., Aylsworth A., Becker A., Brueton L., Lee P., Majewski F., Mulliken J., Suri M. (1999). Mutation analysis of core binding factor A1 in patients with cleidocranial dysplasia. Am. J. Hum. Genet..

[141] Zhou G., Chen Y., Zhou L., Thirunavukkarasu K., Hecht J., Chitayat D., Gelb B.D., Pirinen S., Berry S.A., Greenberg C.R. (1999). CBFA1 mutation analysis and functional correlation with phenotypic variability in cleidocranial dysplasia. Hum. Mol. Genet..

[142] D’Souza R., Aberg T., Gaikwad J., Cavender A., Owen M., Karsenty G., Thesleff I. (1999). Cbfa1 is required for epithelial-mesenchymal interactions regulating tooth development in mice. Development.

[143] Chen S., Gluhak-Heinrich J., Wang Y.H., Wu Y.M., Chuang H.H., Chen L., Yuan G.H., Dong J., Gay I., MacDougall M. (2009). Runx2, OSX, and DSPP in tooth development. J. Dent. Res..

[144] Camilleri S., McDonald F. (2006). Runx2 and dental development. Eur. J. Oral Sci..

[145] Jiang H., Sodek J., Karsenty G., Thomas H., Ranly D., Chen J. (1999). Expression of core binding factor Osf2/Cbfa-1 and bone sialoprotein in tooth development. Mech. Dev..

[146] Hirata A., Sugahara T., Nakamura H. (2008). Localization of runx2, osterix, and osteopontin in tooth root formation in rat molars. J. Histochem. Cytochem..

[147] Han J., He H. (2015). Expression and function of osteogenic genes runt-related transcription factor 2 and osterix in orthodontic tooth movement in rats. Int. J. Clin. Exp. Pathol..

[148] Chen S., Rani S., Wu Y., Unterbrink A., Gu T.T., Gluhak-Heinrich J., Chuang H.-H., MacDougall M. (2005). Differential regulation of dentin sialophosphoprotein expression by runx2 during odontoblast cytodifferentiation. J. Biol. Chem..

[149] James M.J., Järvinen E., Wang X.-P., Thesleff I. (2006). Different roles of runx2 during early neural crest-derived bone and tooth development. J. Bone Miner. Res..

[150] Bufalino A., Paranaíba L., Gouvêa A., Gueiros L.A., Martelli-Júnior H., Junior J., Lopes M., Graner E., De Almeida O., Vargas P. (2011). Cleidocranial dysplasia: Oral features and genetic analysis of 11 patients. Oral Dis..

[151] Gaikwad J., Hoffmann M., Cavender A., Bronckers A., D’Souza R. (2001). Molecular insights into the lineage-specific determination of odontoblasts: The role of cbfa1. Adv. Dent. Res..

[152] Miyazaki T., Kanatani N., Rokutanda S., Yoshida C., Toyosawa S., Nakamura R., Takada S., Komori T. (2008). Inhibition of the terminal differentiation of odontoblasts and their transdifferentiation into osteoblasts in Runx2 transgenic mice. Arch. Histol. Cytol..

[153] Lee M.-H., Kim Y.-J., Yoon W.-J., Kim J.-I., Kim B.-G., Hwang Y.-S., Wozney J.M., Chi X.-Z., Bae S.-C., Choi K.-Y. (2005). Dlx5 Specifically regulates runx2 type II expression by binding to homeodomain-response elements in the runx2 distal promoter. J. Biol. Chem..

[154] Javed A., Bae J.-S., Afzal F., Gutierrez S., Pratap J., Zaidi S.K., Lou Y., van Wijnen A.J., Stein J.L., Stein G.S. (2008). Structural coupling of Smad and runx2 for execution of the BMP2 osteogenic signal. J. Biol. Chem..

[155] Shen R., Chen M., Wang Y.-J., Kaneki H., Xing L., O’Keefe R.J., Chen D. (2006). Smad6 interacts with runx2 and mediates smad ubiquitin regulatory factor 1-induced runx2 degradation. J. Biol. Chem..

[156] Jani P., Zhang H., Benson M.D., Qin C. (2019). Noggin inhibition of mouse dentinogenesis. J. Oral Biosci..

[157] Cho Y.-D., Yoon W.-J., Woo K.-M., Baek J.-H., Park J.-C., Ryoo H.-M. (2010). The canonical BMP signaling pathway plays a crucial part in stimulation of dentin sialophosphoprotein expression by BMP-2. J. Biol. Chem..

[158] Li Z., Wang W., Xu H., Ning Y., Fang W., Liao W., Zou J., Yang Y., Shao N. (2017). Effects of altered CXCL12/CXCR4 axis on BMP2/Smad/Runx2/Osterix axis and osteogenic gene expressions during osteogenic differentiation of MSCs. Am. J. Transl. Res..

[159] Xiao M., Yao B., Zhang B.-D., Bai Y., Sui W., Wang W., Yu Q. (2019). Stromal-derived Factor-1α signaling is involved in bone morphogenetic protein-2-induced odontogenic differentiation of stem cells from apical papilla via the Smad and Erk signaling pathways. Exp. Cell Res..

[160] Wang X., Liao X., Zhang Y., Wei L., Pang Y. (2020). Schisandrin B regulates MC3T3-E1 subclone 14 cells proliferation and differentiation through BMP2-SMADs-RUNX2-SP7 signaling axis. Sci. Rep..

[161] Nakashima K., Zhou X., Kunkel G., Zhang Z., Deng J.M., Behringer R.R., de Crombrugghe B. (2002). The novel zinc finger-containing transcription factor osterix is required for osteoblast differentiation and bone formation. Cell.

[162] Tomazelli K.B., Modolo F., Trentin A.G., Garcez R.C., Biz M.T. (2015). Temporo-spatial analysis of Osterix, HNK1 and Sox10 during odontogenesis and maxillaries osteogenesis. Tissue Cell.

[163] Takahashi A., Ono N., Ono W. (2017). The fate of Osterix-expressing mesenchymal cells in dental root formation and maintenance. Orthod. Craniofacial Res..

[164] Lee D.S., Roh S.Y., Park J.-C. (2018). The Nfic-osterix pathway regulates ameloblast differentiation and enamel formation. Cell Tissue Res..

[165] Hosoya A., Yukita A., Ninomiya T., Hiraga T., Yoshiba K., Yoshiba N., Kasahara E., Nakamura H. (2013). Localization of SUMOylation factors and Osterix in odontoblast lineage cells during dentin formation and regeneration. Histochem. Cell Biol..

[166] Kim J.-Y., Kim B.-I., Jue S.-S., Park J.H., Shin J.-W. (2011). Localization of osteopontin and osterix in periodontal tissue during orthodontic tooth movement in rats. Angle Orthod..

[167] Kim T., Bae C., Lee J., Kim J., Yang X., de Crombrugghe B., Cho E. (2015). Osterix regulates tooth root formation in a site-specific manner. J. Dent. Res..

[168] Zhang H., Jiang Y., Qin C., Liu Y., Ho S.P., Feng J.Q. (2014). Essential role of Osterix for tooth root but not crown dentin formation. J. Bone Miner. Res..

[169] He Y.D., Sui B.D., Li M., Huang J., Chen S., Wu L.A. (2015). Site-specific function and regulation of Osterix in tooth root formation. Int. Endod. J..

[170] Fiscaletti M., Biggin A., Bennetts B., Wong K., Briody J., Pacey V., Birman C., Munns C.F. (2018). Novel variant in Sp7/Osx associated with recessive osteogenesis imperfecta with bone fragility and hearing impairment. Bone.

[171] Lapunzina P., Aglan M., Temtamy S., Caparros-Martin J.A., Valencia M., Letón R., Martínez-Glez V., Elhossini R., Amr K., Vilaboa N. (2010). Identification of a frameshift mutation in Osterix in a patient with recessive osteogenesis imperfecta. Am. J. Hum. Genet..

[172] Yang G., Li X., Yuan G., Liu P., Fan M. (2014). The effects of Osterix on the proliferation and odontoblastic differentiation of human dental papilla cells. J. Endod..

[173] Bae J.-M., Clarke J.C., Rashid H., Adhami M.D., McCullough K., Scott J.S., Chen H., Sinha K.M., De Crombrugghe B., Javed A. (2018). Specificity protein 7 is required for proliferation and differentiation of ameloblasts and odontoblasts. J. Bone Miner. Res..

[174] Yu M., Jiang Z., Wang Y., Xi Y., Yang G. (2019). Molecular mechanisms for short root anomaly. Oral Dis..

[175] Liu H., Lin H., Zhang L., Sun Q., Yuan G., Zhang L., Chen S., Chen Z. (2013). miR-145 and miR-143 regulate odontoblast differentiation through targeting Klf4 and Osx genes in a feedback loop. J. Biol. Chem..

[176] Celil A.B., Hollinger J.O., Campbell P.G. (2005). Osx transcriptional regulation is mediated by additional pathways to BMP2/Smad signaling. J. Cell. Biochem..

[177] Mandal C.C., Drissi H., Choudhury G.G., Ghosh-Choudhury N. (2010). Integration of phosphatidylinositol 3-kinase, akt kinase, and Smad signaling pathway in BMP-2-induced Osterix expression. Calcif. Tissue Res..

[178] Lee M.-H., Kwon T.-G., Park H.-S., Wozney J.M., Ryoo H.-M. (2003). BMP-2-induced Osterix expression is mediated by Dlx5 but is independent of Runx2. Biochem. Biophys. Res. Commun..

[179] Matsubara T., Kida K., Yamaguchi A., Hata K., Ichida F., Meguro H., Aburatani H., Nishimura R., Yoneda T. (2008). BMP2 Regulates Osterix through Msx2 and Runx2 during Osteoblast differentiation. J. Biol. Chem..

[180] Tao H., Lin H., Sun Z., Pei F., Zhang J., Chen S., Liu H., Chen Z. (2019). *Klf4* Promotes Dentinogenesis and odontoblastic differentiation via modulation of TGF-β signaling pathway and interaction with histone acetylation. J. Bone Miner. Res..

[181] Lee D.-S., Park J.-T., Kim H.-M., Ko J.S., Son H.-H., Gronostajski R.M., Cho M.-I., Choung P.-H., Park J.-C. (2009). Nuclear factor I-C is essential for odontogenic cell proliferation and odontoblast differentiation during tooth root development. J. Biol. Chem..

[182] Morasso M.I., Grinberg A., Robinson G., Sargent T.D., Mahon K.A. (1999). Placental failure in mice lacking the homeobox gene Dlx3. Proc. Natl. Acad. Sci. USA.

[183] Merlo G.R., Zerega B., Paleari L., Trombino S., Mantero S., Levi G. (2000). Multiple functions of Dlx genes. Int. J. Dev. Biol..

[184] Qiu M., Bulfone A., Ghattas I., Meneses J.J., Christensen L., Sharpe P.T., Presley R., Pedersen R.A., Rubenstein J.L.R. (1997). Role of the Dlx homeobox genes in proximodistal patterning of the branchial arches: Mutations of Dlx-1, Dlx-2, and Dlx-1 andDlx-2 alter morphogenesis of proximal skeletal and soft tissue structures derived from the first and second arches. Dev. Biol..

[185] Zhao Z., Stock D.W., Buchanan A.V., Weiss K.M. (2000). Expression of Dlx genes during the development of the murine dentition. Dev. Genes Evol..

[186] Robinson G.W., Mahon K.A. (1994). Differential and overlapping expression domains of Dlx-2 and Dlx-3 suggest distinct roles for Distal-less homeobox genes in craniofacial development. Mech. Dev..

[187] Weiss K.M., Bollekens J., Ruddle F.H., Takashita K. (1994). Distal-less and other homeobox genes in the development of the dentition. J. Exp. Zool..

[188] Lézot F., Thomas B., Greene S.R., Hotton D., Yuan Z., Castaneda B., Bolaños A., Depew M., Sharpe P., Gibson C.W. (2008). Physiological implications of DLX homeoproteins in enamel formation. J. Cell. Physiol..

[189] Ghoul-Mazgar S., Hotton D., Lézot F., Blin-Wakkach C., Asselin A., Sautier J.-M., Berdal A. (2005). Expression pattern of Dlx3 during cell differentiation in mineralized tissues. Bone.

[190] Dong J., Amor D., Aldred M.J., Gu T., Escamilla M., MacDougall M. (2005). DLX3 mutation associated with autosomal dominant amelogenesis imperfecta with taurodontism. Am. J. Med. Genet. Part A.

[191] Lee S.K., Lee Z.H., Lee S.J., Ahn B.D., Kim Y.J., Lee S.H., Kim J.W. (2008). DLX3 mutation in a new family and its phenotypic variations. J. Dent. Res..

[192] Wright J.T., Hong S.P., Simmons D., Daly B., Uebelhart D., Luder H.U. (2008). DLX3 c.561_562delCT mutation causes attenuated phenotype of tricho-dento-osseous syndrome. Am. J. Med. Genet. Part A.

[193] Price J.A., Wright J.T., Kula K., Bowden D.W., Hart T.C. (1998). A common DLX3 gene mutation is responsible for tricho-dento-osseous syndrome in Virginia and North Carolina families. J. Med. Genet..

[194] Nieminen P., Lukinmaa P.-L., Alapulli H., Methuen M., Suojärvi T., Kivirikko S., Peltola J., Asikainen M., Alaluusua S. (2011). DLX3 homeodomain mutations cause Tricho-Dento-osseous syndrome with novel phenotypes. Cells Tissues Organs.

[195] Li Y., Han D., Zhang H., Liu H., Wong S., Zhao N., Feng H. (2015). Morphological analyses and a novel de novo DLX3 mutation associated with tricho-dento-osseous syndrome in a Chinese family. Eur. J. Oral Sci..

[196] Choi S., Song I., Feng J., Gao T., Haruyama N., Gautam P., Robey P., Hart T.C. (2010). Mutant DLX 3 disrupts odontoblast polarization and dentin formation. Dev. Biol..

[197] Duverger O., Zah A., Isaac J., Sun H.-W., Bartels A.K., Lian J.B., Berdal A., Hwang J., Morasso M.I. (2012). Neural crest deletion of Dlx3 leads to major dentin defects through down-regulation of Dspp. J. Biol. Chem..

[198] Zheng H., Fu J., Chen Z., Yang G., Yuan G. (2022). Dlx3 ubiquitination by nuclear Mdm2 is essential for dentinogenesis in mice. J. Dent. Res..

[199] Viale-Bouroncle S., Felthaus O., Schmalz G., Brockhoff G., Reichert T.E., Morsczeck C. (2012). The transcription factor DLX3 regulates the osteogenic differentiation of human dental follicle precursor cells. Stem Cells Dev..

[200] Mackenzie A., Leeming G., Jowett A., Ferguson M., Sharpe P. (1991). The homeobox gene Hox 7.1 has specific regional and temporal expression patterns during early murine craniofacial embryogenesis, especially tooth development in vivo and in vitro. Development.

[201] Keränen S.V.E., Åberg T., Kettunen P., Thesleff I., Jernvall J. (1998). Association of developmental regulatory genes with the development of different molar tooth shapes in two species of rodents. Dev. Genes Evol..

[202] Tucker A.S., Al Khamis A., Sharpe P.T. (1998). Interactions between Bmp-4 and Msx-1 act to restrict gene expression to odontogenic mesenchyme. Dev. Dyn..

[203] van den Boogaard M.J., Dorland M., Beemer F.A., van Amstel H.K. (2000). MSX1 mutation is associated with orofacial clefting and tooth agenesis in humans. Nat. Genet..

[204] Lidral A., Reising B. (2002). The role of MSX1 in human tooth agenesis. J. Dent. Res..

[205] Satokata I., Maas R.L. (1994). Msx1 deficient mice exhibit cleft palate and abnormalities of craniofacial and tooth development. Nat. Genet..

[206] Vastardis H., Karimbux N., Guthua S.W., Seidman J., Seidman C.E. (1996). A human MSX1 homeodomain missense mutation causes selective tooth agenesis. Nat. Genet..

[207] Wang Y., Kong H., Mues G., D’Souza R. (2011). Msx1 mutations: How do they cause tooth agenesis?. J. Dent. Res..

[208] Bei M., Maas R. (1998). FGFs and BMP4 induce both Msx1-independent and Msx1-dependent signaling pathways in early tooth development. Development.

[209] Xin T., Zhang T., Li Q., Yu T., Zhu Y., Yang R., Zhou Y. (2018). A novel mutation of MSX1 in oligodontia inhibits odontogenesis of dental pulp stem cells via the ERK pathway. Stem Cell Res. Ther..

[210] Feng X.-Y., Zhao Y.-M., Wang W.-J., Ge L.-H. (2013). *Msx1* regulates proliferation and differentiation of mouse dental mesenchymal cells in culture. Eur. J. Oral Sci..

[211] Feng X.-Y., Wu X.-S., Wang J.-S., Zhang C.-M., Wang S.-L. (2017). Homeobox protein MSX-1 inhibits expression of bone morphogenetic protein 2, bone morphogenetic protein 4, and lymphoid enhancer-binding factor 1 via Wnt/β-catenin signaling to prevent differentiation of dental mesenchymal cells during the late bell stage. Eur. J. Oral Sci..

[212] Yang G., Yuan G., Ye W., Cho K.W.Y., Chen Y. (2014). An atypical canonical bone morphogenetic protein (BMP) signaling pathway regulates Msh homeobox 1 (Msx1) expression during odontogenesis. J. Biol. Chem..

[213] Hu X., Lin C., Ruan N., Huang Z., Zhang Y., Hu X. (2022). Operation of the atypical canonical bone morphogenetic protein signaling pathway during early human odontogenesis. Front. Physiol..

[214] MacKenzie A., Ferguson M., Sharpe P. (1992). Expression patterns of the homeobox gene, Hox-8, in the mouse embryo suggest a role in specifying tooth initiation and shape. Development.

[215] Jowett A., Vainio S., Ferguson M., Sharpe P., Thesleff I. (1993). Epithelial-mesenchymal interactions are required for msx 1 and msx 2 gene expression in the developing murine molar tooth. Development.

[216] Keränen S.V.E., Kettunen P., Åberg T., Thesleff I., Jernvall J. (1999). Gene expression patterns associated with suppression of odontogenesis in mouse and vole diastema regions. Dev. Genes Evol..

[217] Babajko S., de La Dure-Molla M., Jedeon K., Berdal A. (2015). MSX2 in ameloblast cell fate and activity. Front. Physiol..

[218] Bidder M., Latifi T., Towler D.A. (1998). Reciprocal temporospatial patterns of Msx2 and osteocalcin gene expression during murine odontogenesis. J. Bone Miner. Res..

[219] Yamamoto H., Cho S.-W., Kim E.-J., Kim J.-Y., Fujiwara N., Jung H.-S. (2004). Developmental properties of the hertwig’s epithelial root sheath in mice. J. Dent. Res..

[220] Molla M., Descroix V., Aïoub M., Simon S., Castañeda B., Hotton D., Bolaños A., Simon Y., Lezot F., Goubin G. (2010). Enamel protein regulation and dental and periodontal physiopathology in Msx2 mutant mice. Am. J. Pathol..

[221] Jabs E.W., Müller U., Li X., Ma L., Luo W., Haworth I.S., Klisak I., Sparkes R., Warman M.L., Mulliken J.B. (1993). A mutation in the homeodomain of the human MSX2 gene in a family affected with autosomal dominant craniosynostosis. Cell.

[222] Ma L., Golden S., Wu L., Maxson R. (1996). The molecular basis of Boston-type craniosynostosis: The Pro148-->His mutation in the N-terminal arm of the MSX2 homeodomain stabilizes DNA binding without altering nucleotide sequence preferences. Hum. Mol. Genet..

[223] Wilkie A.O., Tang Z., Elanko N., Walsh S., Twigg S., Hurst J.A., Wall S.A., Chrzanowska K., Maxson R.E. (2000). Functional haploinsufficiency of the human homeobox gene MSX2 causes defects in skull ossification. Nat. Genet..

[224] Amri N., Djolé S.X., Petit S., Babajko S., Coudert A.E., Castaneda B., Simon S., Berdal A. (2016). Distorted patterns of dentinogenesis and eruption in Msx2 null mutants: Involvement of sost/sclerostin. Am. J. Pathol..

[225] Aïoub M., Lézot F., Molla M., Castaneda B., Robert B., Goubin G., Néfussi J., Berdal A. (2007). Msx2^−/−^ transgenic mice develop compound amelogenesis imperfecta, dentinogenesis imperfecta and periodental osteopetrosis. Bone.

[226] Hur S.-W., Oh S.-H., Jeong B.-C., Choi H., Kim J.-W., Lee K.-N., Hwang Y.-C., Ryu J.-H., Kim S.-H., Koh J.-T. (2015). COUP-TFII Stimulates dentin sialophosphoprotein expression and mineralization in odontoblasts. J. Dent. Res..

[227] Zhou Y.L., Lei Y., Snead M.L. (2000). Functional antagonism between Msx2 and CCAAT/enhancer-binding protein α in regulating the mouse amelogenin gene expression is mediated by protein-protein interaction. J. Biol. Chem..

[228] Hamada S., Satoh K., Hirota M., Kimura K., Kanno A., Masamune A., Shimosegawa T. (2007). Bone morphogenetic protein 4 induces epithelial-mesenchymal transition through MSX2 induction on pancreatic cancer cell line. J. Cell. Physiol..

[229] Richter A., Valdimarsdottir L., Hrafnkelsdottir H.E., Runarsson J.F., Omarsdottir A.R., Oostwaard D.W.-V., Mummery C., Valdimarsdottir G. (2013). BMP4 Promotes EMT and mesodermal commitment in human embryonic stem cells via SLUG and MSX2. Stem Cells.

[230] Hussein S.M., Duff E.K., Sirard C. (2003). Smad4 and β-catenin co-activators functionally interact with lymphoid-enhancing factor to regulate graded expression of Msx2. J. Biol. Chem..

[231] Rath B., Nam J., Deschner J., Schaumburger J., Tingart M., Grässel S., Grifka J., Agarwal S. (2011). Biomechanical forces exert anabolic effects on osteoblasts by activation of SMAD 1/5/8 through type 1 BMP receptor. Biorheology.

[232] Rodríguez-Carballo E., Ulsamer A., Susperregui A.R., Manzanares-Céspedes C., Sánchez-García E., Bartrons R., Rosa J.L., Ventura F. (2010). Conserved regulatory motifs in osteogenic gene promoters integrate cooperative effects of canonical Wnt and BMP pathways. J. Bone Miner. Res..

[233] Neubüser A., Peters H., Balling R., Martin G.R. (1997). Antagonistic interactions between FGF and BMP signaling pathways: A mechanism for positioning the sites of tooth formation. Cell.

[234] Peters H., Neubüser A., Kratochwil K., Balling R. (1998). *Pax9*-deficient mice lack pharyngeal pouch derivatives and teeth and exhibit craniofacial and limb abnormalities. Genes Dev..

[235] Lin D., Huang Y., He F., Gu S., Zhang G., Chen Y., Zhang Y. (2007). Expression survey of genes critical for tooth development in the human embryonic tooth germ. Dev. Dyn..

[236] Stockton D.W., Das P., Goldenberg M., D’Souza R., Patel P. (2000). Mutation of PAX9 is associated with oligodontia. Nat. Genet..

[237] Bonczek O., Balcar V., Šerý O. (2017). *PAX9* gene mutations and tooth agenesis: A review. Clin. Genet..

[238] Wong S.-W., Han D., Zhang H., Liu Y., Zhang X., Miao M., Wang Y., Zhao N., Zeng L., Bai B. (2017). Nine novel *PAX9* mutations and a distinct tooth agenesis genotype-phenotype. J. Dent. Res..

[239] Blake J.A., Ziman M. (2014). Pax genes: Regulators of lineage specification and progenitor cell maintenance. Development.

[240] Yin W., Bian Z. (2015). The gene network underlying hypodontia. J. Dent. Res..

[241] Kapadia H., Mues G., D’Souza R. (2007). Genes affecting tooth morphogenesis. Orthod. Craniofacial Res..

[242] Kong H., Wang Y., Patel M., Mues G., D’Souza R.N. (2011). Regulation of Bmp4 expression in odontogenic mesenchyme: From simple to complex. Cells Tissues Organs.

[243] Zhou J., Gao Y., Lan Y., Jia S., Jiang R. (2013). Pax9 regulates a molecular network involving Bmp4, Fgf10, Shh signaling and the Osr2 transcription factor to control palate morphogenesis. Development.

[244] Li R., Chen Z., Yu Q., Weng M., Chen Z. (2019). The function and regulatory network of Pax9 gene in palate development. J. Dent. Res..

[245] Seki D., Takeshita N., Oyanagi T., Sasaki S., Takano I., Hasegawa M., Takano-Yamamoto T. (2015). Differentiation of odontoblast-like cells from mouse induced pluripotent stem cells by Pax9 and Bmp4 transfection. STEM CELLS Transl. Med..

[246] Feng J., Jing J., Li J., Zhao H., Punj V., Zhang T., Xu J., Chai Y. (2017). BMP signaling orchestrates a transcriptional network to control the fate of mesenchymal stem cells in mice. Development.

[247] George A., Sabsay B., Simonian P.A., Veis A. (1993). Characterization of a novel dentin matrix acidic phosphoprotein. Implications for induction of biomineralization. J. Biol. Chem..

[248] MacDougall M., Gu T.T., Luan X., Simmons D., Chen J. (1998). Identification of a novel isoform of mouse dentin matrix protein 1: Spatial expression in mineralized tissues. J. Bone Miner. Res..

[249] D’Souza R.N., Cavender A., Sunavala G., Alvarez J., Ohshima T., Kulkarni A.B., MacDougall M. (1997). Gene expression patterns of murine dentin matrix protein 1 (Dmp1) and dentin sialophosphoprotein (DSPP) suggest distinct developmental functions in vivo. J. Bone Miner. Res..

[250] Feng J., Huang H., Lu Y., Ye L., Xie Y., Tsutsui T., Kunieda T., Castranio T., Scott G., Bonewald L. (2003). The Dentin matrix protein 1 (Dmp1) is specifically expressed in mineralized, but not soft, tissues during development. J. Dent. Res..

[251] Baba O., Qin C., Brunn J.C., Wygant J.N., McIntyre B.W., Butler W.T. (2004). Colocalization of dentin matrix protein 1 and dentin sialoprotein at late stages of rat molar development. Matrix Biol..

[252] Hao J., Zou B., Narayanan K., George A. (2004). Differential expression patterns of the dentin matrix proteins during mineralized tissue formation. Bone.

[253] Martinez E.F., da Silva L.A.H., Furuse C., de Araújo N.S., de Araújo V.C. (2009). Dentin matrix protein 1 (DMP1) expression in developing human teeth. Braz. Dent. J..

[254] Toyosawa S., Okabayashi K., Komori T., Ijuhin N. (2004). mRNA expression and protein localization of dentin matrix protein 1 during dental root formation. Bone.

[255] Ye L., MacDougall M., Zhang S., Xie Y., Zhang J., Li Z., Lu Y., Mishina Y., Feng J.Q. (2004). Deletion of dentin matrix protein-1 leads to a partial failure of maturation of predentin into dentin, hypomineralization, and expanded cavities of pulp and root canal during postnatal tooth development. J. Biol. Chem..

[256] Feng J.Q., Ward L.M., Liu S., Lu Y., Xie Y., Yuan B., Yu X., Rauch F., Davis S.I., Zhang S. (2006). Loss of DMP1 causes rickets and osteomalacia and identifies a role for osteocytes in mineral metabolism. Nat. Genet..

[257] Lorenz-Depiereux B., Bastepe M., Benet-Pagès A., Amyere M., Wagenstaller J., Müller-Barth U., Badenhoop K., Kaiser S.M., Rittmaster R.S., Shlossberg A.H. (2006). DMP1 mutations in autosomal recessive hypophosphatemia implicate a bone matrix protein in the regulation of phosphate homeostasis. Nat. Genet..

[258] Turan S., Aydin C., Bereket A., Akcay T., Güran T., Yaralioglu B.A., Bastepe M., Jüppner H. (2010). Identification of a novel dentin matrix protein-1 (DMP-1) mutation and dental anomalies in a kindred with autosomal recessive hypophosphatemia. Bone.

[259] Liu T., Wang J., Xie X., Wang K., Sui T., Liu D., Lai L., Zhao H., Li Z., Feng J.Q. (2019). *DMP1* Ablation in the rabbit results in mineralization defects and abnormalities in haversian canal/osteon microarchitecture. J. Bone Miner. Res..

[260] Yamamoto R., Oida S., Yamakoshi Y. (2015). Dentin Sialophosphoprotein-derived proteins in the dental pulp. J. Dent. Res..

[261] Ritchie H.H., Berry J.E., Somerman M.J., Hanks C.T., Bronckers A.L.J.J., Hotton D., Papagerakis P., Berdal A., Butler W.T. (1997). Dentin sialoprotein (DSP) transcripts: Developmentally-sustained expression in odontoblasts and transient expression in pre-ameloblasts. Eur. J. Oral Sci..

[262] Hou C., Liu Z.X., Tang K.L., Wang M.G., Sun J., Wang J., Li S. (2011). Developmental changes and regional localization of Dspp, Mepe, Mimecan and Versican in postnatal developing mouse teeth. Histochem. J..

[263] Bleicher F., Couble M., Farges J., Couble P., Magloire H. (1999). Sequential expression of matrix protein genes in developing rat teeth. Matrix Biol..

[264] Vijaykumar A., Ghassem-Zadeh S., Vidovic-Zdrilic I., Komitas K., Adameyko I., Krivanek J., Fu Y., Maye P., Mina M. (2019). Generation and characterization of DSPP-Cerulean/DMP1-Cherry reporter mice. Genesis.

[265] Qin C., Brunn J., Cadena E., Ridall A., Tsujigiwa H., Nagatsuka H., Nagai N., Butler W. (2002). The expression of dentin sialophosphoprotein gene in bone. J. Dent. Res..

[266] Chaplet M., Waltregny D., Detry C., Fisher L.W., Castronovo V., Bellahcène A. (2006). Expression of dentin sialophosphoprotein in human prostate cancer and its correlation with tumor aggressiveness. Int. J. Cancer..

[267] Yuan G., Wang Y., Gluhak-Heinrich J., Yang G., Chen L., Li T., Wu L.-A., Chen Z., MacDougall M., Chen S. (2009). Tissue-specific expression of dentin sialophosphoprotein (DSPP) and its polymorphisms in mouse tissues. Cell Biol. Int..

[268] Ogbureke K., Abdelsayed R.A., Kushner H., Li L., Fisher L.W. (2010). Two members of the SIBLING family of proteins, DSPP and BSP, may predict the transition of oral epithelial dysplasia to oral squamous cell carcinoma. Cancer.

[269] Xiao S., Yu C., Chou X., Yuan W., Wang Y., Bu L., Fu G., Qian M., Yang J., Shi Y. (2001). Dentinogenesis imperfecta 1 with or without progressive hearing loss is associated with distinct mutations in DSPP. Nat. Genet..

[270] Zhang X., Zhao J., Li C., Gao S., Qiu C., Liu P., Wu G., Qiang B., Lo W.H., Shen Y. (2001). DSPP mutation in dentinogenesis imperfecta Shields type II. Nat. Genet..

[271] Rajpar M.H., Koch M.J., Davies R.M., Mellody K.T., Kielty C.M., Dixon M.J. (2002). Mutation of the signal peptide region of the bicistronic gene DSPP affects translocation to the endoplasmic reticulum and results in defective dentine biomineralization. Hum. Mol. Genet..

[272] Kim J.-W., Simmer J.P. (2007). Hereditary dentin defects. J. Dent. Res..

[273] Simmer J.P., Zhang H., Moon S.J.H., Donnelly L.A.-J., Lee Y.-L., Seymen F., Koruyucu M., Chan H.-C., Lee K.Y., Wu S. (2022). The modified shields classification and 12 families with defined *DSPP* mutations. Genes.

[274] Hursey R.J., Witkop C.J., Miklashek D., Sackett L.M. (1956). Dentinogenesis imperfecta in a racial isolate with multiple hereditary defects. Oral Surg. Oral Med. Oral Pathol..

[275] Sreenath T., Thyagarajan T., Hall B., Longenecker G., D’Souza R., Hong S., Wright J.T., MacDougall M., Sauk J., Kulkarni A.B. (2003). Dentin sialophosphoprotein knockout mouse teeth display widened predentin zone and develop defective dentin mineralization similar to human dentinogenesis imperfecta type III. J. Biol. Chem..

[276] Heldin C.H., Miyazono K., ten Dijke P. (1997). TGF-b signaling from cell membrane to nucleus through SMAD proteins. Nature.

[277] Shi Y., Massague J. (2003). Mechanisms of TGF-beta signaling from cell membrane to the nucleus. Cell.

[278] Chen D., Zhao M., Mundy G.R. (2004). Bone morphogenetic proteins. Growth Factors.

[279] Miyazono K., Maeda S., Imamura T. (2005). BMP receptor signaling: Transcriptional targets, regulation of signals, and signaling cross-talk. Cytokine Growth Factor Rev..

[280] Sánchez-Duffhues G., García de Vinuesa A., ten Dijke P. (2012). Bone Morphogenetic Proteins Signal Transduction in Vascular Diseases.

[281] Sánchez-Duffhues G., Hiepen C., Knaus P., Dijke P.T. (2015). Bone morphogenetic protein signaling in bone homeostasis. Bone.

[282] Goumans M.-J., Zwijsen A., Ten Dijke P., Bailly S. (2018). Bone morphogenetic proteins in vascular homeostasis and disease. Cold Spring Harb. Perspect. Biol..

[283] Yu P.B., Beppu H., Kawai N., Li E., Bloch K.D. (2005). BMP type II receptor deletion reveals BMP ligand-specific gain of signaling in pulmonary artery smooth muscle cells. J. Biol. Chem..

[284] ten Dijke P., Yamashita H., Sampath T.K., Reddi A.H., Estevez M., Riddle D.L., Ichijo H., Heldin C.H., Miyazono K. (1994). Identification of type I receptors for osteogenic protein-1 and bone morphogenetic protein-4. J. Biol. Chem..

[285] Ebisawa T., Tada K., Kitajima I., Tojo K., Sampath T., Kawabata M., Miyazono K., Imamura T. (1999). Characterization of bone morphogenetic protein-6 signaling pathways in osteoblast differentiation. J. Cell Sci..

[286] Nishitoh H., Ichijo H., Kimura M., Matsumoto T., Makishima F., Yamaguchi A., Yamashita H., Enomoto S., Miyazono K. (1996). Identification of type I and type II serine/threonine kinase receptors for growth/differentiation factor-5. J. Biol. Chem..

[287] David L., Mallet C., Mazerbourg S., Feige J.-J., Bailly S. (2006). Identification of BMP9 and BMP10 as functional activators of the orphan activin receptor-like kinase 1 (ALK1) in endothelial cells. Blood.

[288] Daluiski A., Engstrand T., Bahamonde M.E., Gamer L.W., Agius E., Stevenson S.L., Cox K., Rosen V., Lyons K.M. (2001). Bone morphogenetic protein-3 is a negative regulator of bone density. Nat. Genet..

[289] Mazerbourg S., Klein C., Roh J., Kaivo-Oja N., Mottershead D.G., Korchynskyi O., Ritvos O., Hsueh A.J.W. (2004). Growth differentiation factor-9 signaling is mediated by the type I receptor, activin receptor-like kinase 5. Mol. Endocrinol..

[290] Kokabu S., Gamer L., Cox K., Lowery J., Tsuji K., Raz R., Economides A., Katagiri T., Rosen V. (2012). BMP3 suppresses osteoblast differentiation of bone marrow stromal cells via interaction with Acvr2b. Mol. Endocrinol..

[291] Wu M., Chen G., Li Y.-P. (2016). TGF-β and BMP signaling in osteoblast, skeletal development, and bone formation, homeostasis and disease. Bone Res..

[292] Bhatt S., Diaz R., Trainor P. (2013). Signals and switches in mammalian neural crest cell differentiation. Cold Spring Harb. Perspect. Biol..

[293] Graf D., Malik Z., Hayano S., Mishina Y. (2015). Common mechanisms in development and disease: BMP signaling in craniofacial development. Cytokine Growth Factor Rev..

[294] Derynck R., Zhang Y.E. (2003). Smad-dependent and Smadindependent pathways in TGF-b family signalling. Nature.

[295] Sammar M., Stricker S., Schwabe G.C., Sieber C., Hartung A., Hanke M., Oishi I., Pohl J., Minami Y., Sebald W. (2004). Modulation of GDF5/BRI-b signalling through interaction with the tyrosine kinase receptor Ror2. Genes Cells.

[296] Hagihara M., Endo M., Hata K., Higuchi C., Takaoka K., Yoshikawa H., Yamashita T. (2011). Neogenin, a receptor for bone morphogenetic proteins. J. Biol. Chem..

[297] Nickel J., Dijke P.T., Mueller T.D. (2018). TGF-β family co-receptor function and signaling. Acta Biochim. Biophys. Sin..

[298] Gipson G.R., Goebel E.J., Hart K.N., Kappes E.C., Kattamuri C., McCoy J.C., Thompson T.B. (2020). Structural perspective of BMP ligands and signaling. Bone.

[299] Zouvelou V., Luder H.-U., Mitsiadis T.A., Graf D. (2009). Deletion of BMP7 affects the development of bones, teeth, and other ectodermal appendages of the orofacial complex. J. Exp. Zool. Part B Mol. Dev. Evol..

[300] Feng J., Yang G., Yuan G., Gluhak-Heinrich J., Yang W., Wang L., Chen Z., McDaniel J.S., Donly K.J., Harris S.E. (2011). Abnormalities in the enamel in bmp2-deficient mice. Cells Tissues Organs.

[301] Guo F., Feng J., Wang F., Li W., Gao Q., Chen Z., Shoff L., Donly K.J., Gluhak-Heinrich J., Chun Y.H.P. (2014). Bmp2 deletion causes an amelogenesis imperfecta phenotype via regulating enamel gene expression. J. Cell. Physiol..

[302] Huang X., Wang F., Zhao C., Yang S., Cheng Q., Tang Y., Zhang F., Zhang Y., Luo W., Wang C. (2019). Dentinogenesis and tooth-alveolar bone complex defects in *BMP9/GDF2* knockout mice. Stem Cells Dev..

[303] Wang F., Tao R., Zhao L., Hao X.-H., Zou Y., Lin Q., Liu M.M., Goldman G., Luo D., Chen S. (2021). Differential lncRNA/mRNA expression profiling and functional network analyses in Bmp2 deletion of mouse dental papilla cells. Front. Genet..

[304] Chen Z., Couble M.-L., Mouterfi N., Magloire H., Chen Z., Bleicher F. (2009). Spatial and temporal expression of KLF4 and KLF5 during murine tooth development. Arch. Oral Biol..

[305] Lee H.-K., Lee D.-S., Park S.-J., Cho K.-H., Bae H.-S., Park J.-C. (2014). Nuclear Factor I-C (NFIC) regulates Dentin Sialophosphoprotein (DSPP) and E-cadherin via control of Krüppel-like Factor 4 (KLF4) during dentinogenesis. J. Biol. Chem..

[306] Xu M., Horrell J., Snitow M., Cui J., Gochnauer H., Syrett C.M., Kallish S., Seykora J.T., Liu F., Gaillard D. (2017). WNT10A mutation causes ectodermal dysplasia by impairing progenitor cell proliferation and KLF4-mediated differentiation. Nat. Commun..

[307] Li S., Lin C., Zhang J., Tao H., Liu H., Yuan G., Chen Z. (2018). Quaking promotes the odontoblastic differentiation of human dental pulp stem cells. J. Cell. Physiol..

[308] Huang Z., Yang R., Li R., Zuo Y., Gu F., He M., Bian Z. (2022). Mesenchymal Mycn participates in odontoblastic lineage commitment by regulating Krüppel-like Factor 4 (Klf4) in mice. Stem. Cell Res. Ther..

[309] Lee Y.S., Park Y., Seo Y., Lee H., Park J. (2022). Tubular dentin formation by TGF-β/BMP signaling in dental epithelial cells. Oral Dis..

[310] Celil A.B., Campbell P.G. (2005). BMP-2 and insulin-like growth factor-I mediate Osterix (Osx) expression in human mesenchymal stem cells via the MAPK and protein kinase D signaling pathways. J. Biol. Chem..

[311] Ge X., Li Z., Jing S., Wang Y., Li N., Lu J., Yu J. (2019). Parathyroid hormone enhances the osteo/odontogenic differentiation of dental pulp stem cells via ERK and P38 MAPK pathways. J. Cell. Physiol..

[312] Peng L., Dong G., Xu P., Ren L., Wang C., Aragon M., Zhou X., Ye L. (2010). Expression of Wnt5a in tooth germs and the related signal transduction analysis. Arch. Oral Biol..

[313] Zhang R., Han M., Zheng B., Li Y.-J., Shu Y.-N., Wen J. (2010). Krüppel-like factor 4 interacts with p300 to activate mitofusin 2 gene expression induced by all-trans retinoic acid in VSMCs. Acta Pharmacol. Sin..

[314] Greenblatt M.B., Kim J.-M., Oh H., Park K.H., Choo M.-K., Sano Y., Tye C., Skobe Z., Davis R.J., Park J.M. (2015). p38α MAPK is required for tooth morphogenesis and enamel secretion. J. Biol. Chem..

[315] Choi Y.H., Jeong H.M., Jin Y.-H., Li H., Yeo C.-Y., Lee K.-Y. (2011). Akt phosphorylates and regulates the osteogenic activity of Osterix. Biochem. Biophys. Res. Commun..

[316] Choi Y.H., Choi H.-J., Lee K.-Y., Oh J.-W. (2012). Akt1 regulates phosphorylation and osteogenic activity of Dlx3. Biochem. Biophys. Res. Commun..

[317] Pande S., Browne G., Padmanabhan S., Zaidi S.K., Lian J.B., van Wijnen A.J., Stein J.L., Stein G.S. (2013). Oncogenic cooperation between PI3K/Akt signaling and transcription factor Runx2 promotes the invasive properties of metastatic breast cancer cells. J. Cell. Physiol..

[318] Sun Y., Zheng B., Zhang X.-H., He M., Guo Z.-W., Wen J.-K. (2013). PPAR-γ agonist stabilizes KLF4 protein via activating Akt signaling and reducing KLF4 ubiquitination. Biochem. Biophys. Res. Commun..

[319] Kim B.-G., Kim H.-J., Park H.-J., Kim Y.-J., Yoon W.-J., Lee S.-J., Ryoo H.-M., Cho J.-Y. (2006). Runx2 phosphorylation induced by fibroblast growth factor-2/protein kinase C pathways. Proteomics.

[320] Chew Y.C., Adhikary G., Wilson G.M., Reece E.A., Eckert R.L. (2011). Protein Kinase C (PKC) δ suppresses keratinocyte proliferation by increasing p21Cip1 level by a KLF4 transcription factor-dependent mechanism. J. Biol. Chem..

[321] Jeong H.M., Jin Y.-H., Choi Y.H., Yum J., Choi J.-K., Yeo C.-Y., Lee K.-Y. (2012). PKC signaling inhibits osteogenic differentiation through the regulation of Msx2 function. Biochim. Biophys. Acta.

[322] He S., Choi Y.H., Choi J.-K., Yeo C.-Y., Chun C., Lee K.Y. (2014). Protein kinase a regulates the osteogenic activity of Osterix. J. Cell. Biochem..

[323] Li H., Jeong H.M., Choi Y.H., Kim J.H., Choi J.-K., Yeo C.-Y., Jeong H.G., Jeong T.C., Chun C., Lee K.Y. (2014). Protein kinase A phosphorylates Dlx3 and regulates the function of Dlx3 during osteoblast differentiation. J. Cell. Biochem..

[324] Palazzo E., Kellett M.D., Cataisson C., Bible P.W., Bhattacharya S., Sun H.-W., Gormley A.C., Yuspa S.H., Morasso M.I. (2017). A novel DLX3–PKC integrated signaling network drives keratinocyte differentiation. Cell Death Differ..

[325] Zhu X., Li M., Jia X., Hou W., Yang J., Zhao H., Wang G., Wang J. (2019). The homeoprotein Msx1 cooperates with Pkn1 to prevent terminal differentiation in myogenic precursor cells. Biochimie.

[326] Chen S., Unterbrink A., Kadapakkam S., Dong J., Gu T.T., Dickson J., Chuang H.-H., MacDougall M. (2004). Regulation of the cell type-specific dentin sialophosphoprotein gene expression in mouse odontoblasts by a novel transcription repressor and an activator CCAAT-binding factor. J. Biol. Chem..

[327] He W.-X., Niu Z.-Y., Zhao S.-L., Jin W.-L., Gao J., Smith A.J. (2004). TGF-β activated Smad signalling leads to a Smad3-mediated down-regulation of DSPP in an odontoblast cell line. Arch. Oral Biol..

[328] Narayanan K., Ramachandran A., Peterson M.C., Hao J., Kolstø A.-B., Friedman A.D., George A. (2004). The CCAAT enhancer-binding protein (C/EBP)β and Nrf1 interact to regulate dentin sialophosphoprotein (DSPP) gene expression during odontoblast differentiation. J. Biol. Chem..

[329] Kim J., Choi H., Jeong B., Oh S., Hur S., Lee B., Kim S., Nör J., Koh J., Hwang Y. (2014). Transcriptional factor ATF6 is involved in odontoblastic differentiation. J. Dent. Res..

[330] Zhou C., Yang G., Chen M., Wang C., He L., Xiang L., Chen D., Ling J., Mao J.J. (2015). Lhx8 mediated Wnt and TGFβ pathways in tooth development and regeneration. Biomaterials.

[331] Chen Z., Zhang Q., Wang H., Li W., Wang F., Wan C., Deng S., Chen H., Yin Y., Li X. (2017). Klf5 mediates odontoblastic differentiation through regulating dentin-specific extracellular matrix gene expression during mouse tooth development. Sci. Rep..

[332] Martín-González J., Pérez-Pérez A., Cabanillas-Balsera D., Vilariño-García T., Sánchez-Margalet V., Segura-Egea J.J. (2018). Leptin stimulates DMP-1 and DSPP expression in human dental pulp via MAPK 1/3 and PI3K signaling pathways. Arch. Oral Biol..

[333] Deng Z., Yan W., Dai X., Chen M., Qu Q., Wu B., Zhao W. (2021). N-cadherin regulates the odontogenic differentiation of dental pulp stem cells via β-catenin activity. Front. Cell Dev. Biol..

[334] Harland R.M. (2008). A protein scaffold plays matchmaker for chordin. Cell.

[335] Gajos-Michniewicz A., Piastowska A.W., Russell J.A., Ochedalski T. (2010). Follistatin as a potent regulator of bone metabolism. Biomarkers.

[336] Khokha M.K., Hsu D., Brunet L.J., Dionne M., Harland R.M. (2003). Gremlin is the BMP antagonist required for maintenance of SHH and FGF signals during limb patterning. Nat. Genet..

[337] Guha U., Gomes W.A., Kobayashic T., Pestell R.G., Kessler J.A. (2002). In vivo evidence that BMP signaling is necessary for apoptosis in the mouse limb. Dev. Biol..

[338] Wang Y., Li L., Zheng Y., Yuan G., Yang G., He F., Chen Y. (2012). BMP activity is required for tooth development from the lamina to bud stage. J. Dent. Res..

[339] Gong Y., Krakow D., Marcelino J.M., Wilkin D.J., Chitayat D., Babul-Hirji R., Hudgins L., Cremers C.W., Cremers F.P., Brunner H.G. (1999). Heterozygous mutations in the gene encoding noggin affect human joint morphogenesis. Nat. Genet..

[340] Brown D.J., Kim T.B., Petty E.M., Downs C.A., Martin D.M., Strouse P.J., Moroi S.E., Milunsky J.M., Lesperance M.M. (2002). Autosomal dominant stapes ankylosis with broad thumbs and toes, hyperopia, and skeletal anomalies is caused by heterozygous nonsense and frameshift mutations in NOG, the gene encoding noggin. Am. J. Hum. Genet..

[341] Moffett S.P., Dillon K.A., Yerges L.M., Goodrich L.J., Nestlerode C., Bunker C.H., Wheeler V.W., Patrick A.L., Zmuda J.M. (2009). Identification and association analysis of single nucleotide polymorphisms in the human noggin (NOG) gene and osteoporosis phenotypes. Bone.

[342] Gutiérrez-Prieto S.J., Torres-López D.M., García-Robayo D.A., Rey-Cubillos J.A., Gómez-Rodríguez M. (2021). Clinical and molecular study of the NOG gene in families with mandibular micrognathism. Eur. J. Dent..

[343] Warren S.M., Brunet L.J., Harland R.M., Economides A., Longaker M.T. (2003). The BMP antagonist noggin regulates cranial suture fusion. Nature.

[344] Wijgerde M., Karp S., McMahon J., McMahon A.P. (2005). Noggin antagonism of BMP4 signaling controls development of the axial skeleton in the mouse. Dev. Biol..

[345] Devlin R.D., Du Z., Pereira R.C., Kimble R.B., Economides A.N., Jorgetti V., Canalis E. (2003). Skeletal overexpression of noggin results in osteopenia and reduced bone formation. Endocrinology.

[346] Wu X.-B., Li Y., Schneider A., Yu W., Rajendren G., Iqbal J., Yamamoto M., Alam M., Brunet L.J., Blair H.C. (2003). Impaired osteoblastic differentiation, reduced bone formation, and severe osteoporosis in noggin-overexpressing mice. J. Clin. Investig..

[347] Yuan G., Yang G., Zheng Y., Zhu X., Chen Z., Zhang Z., Chen Y. (2015). The non-canonical BMP and Wnt/β-catenin signaling pathways orchestrate early tooth development. Development.

[348] Lee S.-Y., Auh Q.-S., Kang S.-K., Kim H.-J., Lee J.-W., Noh K., Jang J.-H., Kim E.-C. (2014). Combined effects of dentin sialoprotein and bone morphogenetic protein-2 on differentiation in human cementoblasts. Cell Tissue Res..

[349] Gazzerro E., Gangji V., Canalis E. (1998). Bone morphogenetic proteins induce the expression of noggin, which limits their activity in cultured rat osteoblasts. J. Clin. Investig..

[350] Kantaputra P., Kaewgahya M., Hatsadaloi A., Vogel P., Kawasaki K., Ohazama A., Cairns J.K. (2015). *GREMLIN 2* mutations and dental anomalies. J. Dent. Res..

[351] Vogel P., Liu J., Platt K.A., Read R.W., Thiel M., Vance R.B., Brommage R. (2014). Malformation of incisor teeth in *Grem2^−/−^* mice. Veter. Pathol..

[352] Nagatomo K.J., Tompkins K.A., Fong H., Zhang H., Foster B.L., Chu E.Y., Murakami A., Stadmeyer L., Canalis E., Somerman M.J. (2008). Transgenic overexpression of gremlin results in developmental defects in enamel and dentin in mice. Connect. Tissue Res..

[353] Wang C.-L., Xiao F., Wang C.-D., Zhu J.-F., Shen C., Zuo B., Wang H., Li D., Wang X.-Y., Feng W.-J. (2016). Gremlin2 suppression increases the BMP-2-induced osteogenesis of human bone marrow-derived mesenchymal stem cells via the BMP-2/Smad/Runx2 signaling pathway. J. Cell. Biochem..

[354] Liu H., Han X., Yang H., Cao Y., Zhang C., Du J., Diao S., Fan Z. (2020). GREM1 inhibits osteogenic differentiation, senescence and BMP transcription of adipose-derived stem cells. Connect. Tissue Res..

[355] Guan X., He Y., Li Y., Shi C., Wei Z., Zhao R., Han Y., Pan L., Yang J., Hou T.Z. (2022). Gremlin aggravates periodontitis via activating the NF-κB signaling pathway. J. Periodontol..

[356] Abreu J.G., Coffinier C., Larraín J., Oelgeschläger M., De Robertis E. (2002). Chordin-like CR domains and the regulation of evolutionarily conserved extracellular signaling systems. Gene.

[357] Itoh N., Ohta H. (2010). Secreted bone morphogenetic protein antagonists of the chordin family. Biomol. Concepts.

[358] Piccolo S., Sasai Y., Lu B., De Robertis E.M. (1996). Dorsoventral patterning in xenopus: Inhibition of ventral signals by direct binding of chordin to BMP-4. Cell.

[359] Larrain J., Bachiller D., Lu B., Agius E., Piccolo S., De Robertis E. (2000). BMP-binding modules in chordin: A model for signalling regulation in the extracellular space. Development.

[360] Nakayama N., Han C.-Y.E., Scully S., Nishinakamura R., He C., Zeni L., Yamane H., Chang D., Yu D., Yokota T. (2001). A novel chordin-like protein inhibitor for bone morphogenetic proteins expressed preferentially in mesenchymal cell lineages. Dev. Biol..

[361] Nakayama N., Han C.-Y.E., Cam L., Lee J.I., Pretorius J., Fisher S., Rosenfeld R., Scully S., Nishinakamura R., Duryea D. (2004). A novel chordin-like BMP inhibitor, CHL2, expressed preferentially in chondrocytes of developing cartilage and osteoarthritic joint cartilage. Development.

[362] Stottmann R.W., Anderson R.M., Klingensmith J. (2001). The BMP antagonists chordin and noggin have essential but redundant roles in mouse mandibular outgrowth. Dev. Biol..

[363] Zhang N., Ferguson C.M., O’Keefe R.J., Puzas J.E., Rosier R.N., Reynolds P.R. (2002). A role for the BMP antagonist chordin in endochondral ossification. J. Bone Miner. Res..

[364] Ryan A.K., Goodship J.A., Wilson D.I., Philip N., Levy A., Seidel H., Schuffenhauer S., Oechsler H., Belohradsky B., Prieur M. (1997). Spectrum of clinical features associated with interstitial chromosome 22q11 deletions: A European collaborative study. J. Med. Genet..

[365] Bachiller D., Klingensmith J., Shneyder N., Tran U., Anderson R., Rossant J., De Robertis E.M. (2003). The role of chordin/Bmp signals in mammalian pharyngeal development and DiGeorge syndrome. Development.

[366] Webb T.R., Matarin M., Gardner J.C., Kelberman D., Hassan H., Ang W., Michaelides M., Ruddle J.B., Pennell C.E., Yazar S. (2012). X-linked megalocornea caused by mutations in CHRDL1 identifies an essential role for ventroptin in anterior segment development. Am. J. Hum. Genet..

[367] Chen D., Liu Y., Shu G., Chen C., Sullivan D.A., Kam W.R., Hann S., Fowler M., Warman M.L. (2020). Ocular manifestations of chordin-like 1 knockout mice. Cornea.

[368] Petryk A., Shimmi O., Jia X., Carlson A.E., Tervonen L., Jarcho M.P., O’Connor M.B., Gopalakrishnan R. (2005). Twisted gastrulation and chordin inhibit differentiation and mineralization in MC3T3-E1 osteoblast-like cells. Bone.

[369] Liu T., Li B., Zheng X.-F., Jiang S.-D., Zhou Z.-Z., Xu W.-N., Zheng H.-L., Wang C.-D., Zhang X.-L., Jiang L.-S. (2019). Chordin-like 1 improves osteogenesis of bone marrow mesenchymal stem cells through enhancing BMP4-SMAD pathway. Front. Endocrinol..

[370] World Health Organization (2003). Dental Diseases and Oral Health. https://www.who.int/oral_health/publications/en/orh_fact_sheet.pdf.

[371] World Health Organization (2017). Oral Health. https://www.who.int/publications/i/item/WHO-NMH-NHD-17.12.

[372] Medina-Fernandez I., Celiz A.D. (2018). Acellular biomaterial strategies for endodontic regeneration. Biomater. Sci..

[373] Bernabe E., Marcenes W., Hernandez C.R., Bailey J., Abreu L.G., Alipour V., Amini S., Arabloo J., Arefi Z., GBD 2017 Oral Disorders Collaborators (2020). Global, regional, and national levels and trends in burden of oral conditions from 1990 to 2017: A systematic analysis for the global burden of disease 2017 study. J. Dent. Res..

[374] Kazeminia M., Abdi A., Shohaimi S., Jalali R., Vaisi-Raygani A., Salari N., Mohammadi M. (2020). Dental caries in primary and permanent teeth in children’s worldwide, 1995 to 2019: A systematic review and meta-analysis. Head Face Med..

[375] Soares D.G., Bordini E.A.F., Swanson W.B., Costa C.A.D.S., Bottino M.C. (2021). Platform technologies for regenerative endodontics from multifunctional biomaterials to tooth-on-a-chip strategies. Clin. Oral Investig..

[376] Burke F.J.T., Lucarotti P.S.K. (2008). How long do direct restorations placed within the general dental services in England and Wales survive?. Br. Dent. J..

[377] Tziafas D., Smith A., Lesot H. (1999). Designing new treatment strategies in vital pulp therapy. J. Dent..

[378] Goldberg M., Smith A.J. (2004). Cells and extracellular matrices of dentin and pulp: A biological basis for repair and tissue engineering. Crit. Rev. Oral Biol. Med..

[379] Murray P.E., Garcia-Godoy F., Hargreaves K.M. (2007). Regenerative endodontics: A review of current status and a call for action. J. Endod..

[380] Langer R., Vacanti J.P. (1993). Tissue engineering. Science.

[381] Gronthos S., Mankani M., Brahim J., Robey P.G., Shi S. (2000). Postnatal human dental pulp stem cells (DPSCs) in vitro and in vivo. Proc. Natl. Acad. Sci. USA.

[382] Miura M., Gronthos S., Zhao M., Lu B., Fisher L.W., Robey P.G., Shi S. (2003). SHED: Stem cells from human exfoliated deciduous teeth. Proc. Natl. Acad. Sci. USA.

[383] Sonoyama W., Liu Y., Yamaza T., Tuan R.S., Wang S., Shi S., Huang G.T. (2008). Characterization of the apical papilla and its residing stem cells from human immature permanent teeth: A pilot study. J. Endod..

[384] Nakashima M., Iohara K., Murakami M., Nakamura H., Sato Y., Ariji Y., Matsushita K. (2017). Pulp regeneration by transplantation of dental pulp stem cells in pulpitis: A pilot clinical study. Stem Cell Res. Ther..

[385] Xuan K., Li B., Guo H., Sun W., Kou X., He X., Zhang Y., Sun J., Liu A., Liao L. (2018). Deciduous autologous tooth stem cells regenerate dental pulp after implantation into injured teeth. Sci. Transl. Med..

[386] Nakashima M. (1994). Induction of dentin formation on canine amputated pulp by recombinant human bone morphogenetic proteins (BMP)-2 and -4. J. Dent. Res..

[387] Kirker-Head C. (2000). Potential applications and delivery strategies for bone morphogenetic proteins. Adv. Drug Deliv. Rev..

[388] Goldberg M., Six N., Decup F., Buch D., Majd E.S., Lasfargues J.-J., Salih E., Stanislawski L. (2001). Application of bioactive molecules in pulp-capping situations. Adv. Dent. Res..

[389] Iohara K., Nakashima M., Ito M., Ishikawa M., Nakasima A., Akamine A. (2004). Dentin regeneration by dental pulp stem cell therapy with recombinant human bone morphogenetic protein 2. J. Dent. Res..

[390] Saito T., Ogawa M., Hata Y., Bessho K. (2004). Acceleration effect of human recombinant bone morphogenetic protein-2 on differentiation of human pulp cells into odontoblasts. J. Endod..

[391] Chakka L.R.J., Vislisel J., Vidal C.D.M.P., Biz M.T., Salem A.K., Cavalcanti B.N. (2020). Application of BMP-2/FGF-2 gene–activated scaffolds for dental pulp capping. Clin. Oral Investig..

[392] Um I.-W., Ku J.-K., Kim Y.-K., Lee B.-K., Leem D.H. (2020). Histological review of demineralized dentin matrix as a carrier of rhBMP-2. Tissue Eng. Part B Rev..

[393] Wang W., Dang M., Zhang Z., Hu J., Eyster T.W., Ni L., Ma P.X. (2016). Dentin regeneration by stem cells of apical papilla on injectable nanofibrous microspheres and stimulated by controlled BMP-2 release. Acta Biomater..

[394] U.S. Food and Drug Administration (2007). Summary of Safety and Effectiveness Data.

[395] King G.N., Hughes F.J. (2001). Bone morphogenetic protein-2 stimulates cell recruitment and cementogenesis during early wound healing. J. Clin. Periodontol..

[396] King G.N., Cochrant D.L. (2002). Factors that modulate the effects of bone morphogenetic protein-induced periodontal regeneration: A critical review. J. Periodontol..

[397] Jones A.A., Buser D., Schenk R.K., Wozney J., Cochran D.L. (2006). The effect of rhBMP-2 around endosseous implants with and without membranes in the canine model. J. Periodontol..

[398] Jung R.E., Weber F.E., Thoma D.S., Ehrbar M., Cochran D.L., Hämmerle C.H.F. (2008). Bone morphogenetic protein-2 enhances bone formation when delivered by a synthetic matrix containing hydroxyapatite/tricalciumphosphate. Clin. Oral Implant. Res..

[399] De Freitas R.M., Spin-Neto R., Junior E.M., Pereira L.A.V.D., Wikesjo U.M., Susin C. (2013). Alveolar ridge and maxillary sinus augmentation using rhBMP-2: A systematic review. Clin. Implant Dent. Relat. Res..

[400] Talley A.D., Kalpakci K.N., Shimko D.A., Zienkiewicz K.J., Cochran D.L., Guelcher S.A. (2016). Effects of recombinant human bone morphogenetic protein-2 dose and ceramic composition on new bone formation and space maintenance in a canine mandibular ridge saddle defect model. Tissue Eng. Part A.

[401] Batool F., Strub M., Petit C., Bugueno I.M., Bornert F., Clauss F., Huck O., Kuchler-Bopp S., Benkirane-Jessel N. (2018). Periodontal tissues, maxillary jaw bone, and tooth regeneration approaches: From animal models analyses to clinical applications. Nanomaterials.

[402] Boda S.K., Almoshari Y., Wang H., Wang X., Reinhardt R.A., Duan B., Wang D., Xie J. (2018). Mineralized nanofiber segments coupled with calcium-binding BMP-2 peptides for alveolar bone regeneration. Acta Biomater..

[403] Le B., Too J., Tan T., Smith R., Nurcombe V., Cool S., Yu N. (2021). Application of a BMP2-binding heparan sulphate to promote periodontal regeneration. Eur. Cells Mater..

[404] Nakashima M., Reddi A.H. (2003). The application of bone morphogenetic proteins to dental tissue engineering. Nat. Biotechnol..

[405] Nakashima M. (2005). Bone morphogenetic proteins in dentin regeneration for potential use in endodontic therapy. Cytokine Growth Factor Rev..

[406] Lee S.-Y., Kim S.-Y., Park S.-H., Kim J.-J., Jang J.-H., Kim E.-C. (2012). Effects of recombinant dentin sialoprotein in dental pulp cells. J. Dent. Res..

[407] Ozer A., Yuan G.H., Yang G.B., Wang F., Li W., Yang Y., Guo F., Gao Q.P., Shoff L., Chen Z. (2013). Domain of dentine sialoprotein mediates proliferation and differentiation of human periodontal ligament stem cells. PLoS ONE.

[408] Wan C., Yuan G., Luo D., Zhang L., Lin H., Liu H., Chen L., Yang G., Chen S., Chen Z. (2016). The Dentin Sialoprotein (DSP) domain regulates dental mesenchymal cell differentiation through a novel surface receptor. Sci. Rep..

[409] Li W., Chen L., Chen Z., Wu L., Feng J., Wang F., Shoff L., Li X., Donly K.J., MacDougall M. (2017). Dentin sialoprotein facilitates dental mesenchymal cell differentiation and dentin formation. Sci. Rep..

[410] James A.W., Lachaud G., Shen J., Asatrian G., Nguyen V., Zhang X., Ting K., Soo C. (2016). A review of the clinical side effects of bone morphogenetic protein-2. Tissue Eng. Part B Rev..

